# Clinical characteristics of outpatients with influenza-B-associated pneumonia and molecular evolution of influenza B virus in Beijing, China, during the 2021–2022 influenza season

**DOI:** 10.1007/s00705-023-05957-6

**Published:** 2024-01-18

**Authors:** Yanxin Wang, Yafen Liu, Yue Wang, Huan Mai, Yuanyuan Chen, Yifan Zhang, Ying Ji, Xu Cong, Yan Gao

**Affiliations:** 1 Department of Infectious Diseases, Peking University People’s Hospital, Beijing, China; 2 Peking University Hepatology Institute, Peking University People’s Hospital, Beijing, China

## Abstract

We analyzed the clinical characteristics of outpatients with influenza-B-associated pneumonia during the 2021–2022 influenza season and analyzed the molecular epidemiology and evolution of influenza B virus. The presence of influenza B virus was confirmed by reverse transcription polymerase chain reaction (RT-PCR). Electronic medical records were used to collect and analyze data of outpatients. The HA and NA genes were phylogenetically analyzed using ClustalW 2.10 and MEGA 11.0. Out of 1569 outpatients who tested positive for influenza B virus, 11.7% (184/1569) developed pneumonia, and of these, 19.0% (35/184) had underlying diseases. Fever, cough, and sore throat were the most common symptoms. Among the complications, acute respiratory distress syndrome (ARDS), acute kidney injury (AKI), and shock accounted for 2.7% (5/184), 4.9% (9/184), and 1.6% (3/184), respectively. Of the outpatients, 2.7% (5/184) were admitted to the hospital, and 0.5% (1/184) of them died. All of the strains from Beijing were identified as belonging to the B/Victoria lineage. The HA and NA gene sequences of 41 influenza B viruses showed high similarity to each other, and all of them belonged to clade 1A.3. Compared with the vaccine strain B/Washington/02/2019, all of the isolates contained N150K, G181E, and S194D mutations. S194D, E195K, and K200R mutations were detected in the 190 helix of the receptor binding region of HA. Co-mutations of H122Q, A127T, P144L, N150K, G181E, S194D, and K200R in HA and D53N, N59S, and G233E in NA were detected in 78.0% (32/41) of the isolates, and 56.3% (18/32) of these were from outpatients with influenza-B-associated pneumonia. Influenza outpatients with underlying diseases were more likely to develop pneumonia. No significant differences were observed in clinical symptoms or laboratory results between outpatients with and without pneumonia, so testing for influenza virus seems to be a good choice. The observed amino acid variations suggest that current vaccines might not provide effective protection.

**Supplementary Information:**

The online version contains supplementary material available at 10.1007/s00705-023-05957-6.

## Introduction

Influenza is a respiratory infectious disease caused by influenza virus, which results in 3–5 million severe cases and 290,000-650,000 deaths worldwide every year [1]. In China, about 88,100 influenza-related respiratory deaths occur annually [2]. Presently, seasonal epidemics are caused by influenza A and B viruses. Because of its pandemic potential and predominance in seasonal influenza epidemics, influenza A virus is usually the primary focus of influenza surveillance. Nevertheless, influenza B virus can cause local outbreaks and serious and fatal cases of seasonal influenza [3, 4]. Researchers from China have found that influenza-related mortality is higher during the season in which influenza B virus dominates than in the season in which influenza A virus dominates. They also discovered that more than 50% of all cases of influenza-related decease were associated with influenza B viruses [5]. A structured literature review has suggested that the impact of influenza B activity might be similar to that of influenza A during periods of high intensity [6]. Caini and colleagues conducted an investigation that included more than 1.8 million cases of influenza that occurred in 31 countries in the 21st century, describing the epidemiological characteristics of influenza B viruses. They found that influenza B viruses accounted for a significant proportion of global influenza infections, resulting in a higher burden of disease than previously thought [7]. Therefore, it is necessary to pay more attention to and strengthen research on influenza B virus.

Influenza-related pneumonia is the most important clinical manifestation of severe influenza, accounting for 20–50% of influenza-related hospitalizations [8]. The mortality rate of influenza-B-related pneumonia is similar to that of influenza-A-related pneumonia (8.5% vs. 9.4%) [9]. A prospective, observational cohort study showed that the incidence of pneumonia was 37.5% in hospitalized patients with influenza A and 28.0% with influenza B, that the mortality rate was 16.3% and 10.0%, respectively, and that these differences were not statistically significant [10]. There is, however, little information on outpatients with influenza-B-related pneumonia due to previous studies focusing more on influenza A virus and inpatients. We therefore analyzed the clinical characteristics of outpatients with influenza B virus pneumonia in order to be able to identify and diagnose the disease early and thereby reduce hospitalization and mortality.

Seasonal influenza declined strongly during the coronavirus disease 2019 (COVID-19) pandemic, primarily due to public health measures and travel restrictions. The decrease in circulating strains as well as the fact that some countries failed to maintain influenza surveillance made it difficult to select strains for influenza vaccine formulations, which led to a further increase in the vaccine mismatch rate. The 2021–2022 influenza season, the first season after there had been almost no influenza outbreaks worldwide, was dominated by influenza B viruses. In this study, the hemagglutinin (HA)

and neuraminidase (NA) genes of influenza B virus isolates were sequenced and phylogenetically analyzed in order to provide data for screening vaccine strains and to explore the relationship between clinical characteristics and genetic mutations.

## Materials and methods

### Patients and collection of specimens

During the 2021–2022 influenza season (October to the following April), 12,691 nasal swab specimens were collected from outpatients with influenza-like illness at Peking University People’s Hospital (PKUPH). Colloidal gold screening yielded 1608 positive results for influenza A and/or B viruses, of which 1569 were positive for influenza B virus. The inclusion criteria were as follows: (1) age ≥ 16 years old, (2) outpatients were diagnosed with influenza B virus infection, (3) new lung infiltrations were observed in chest CT scans, (4) individuals volunteered to participate in the study. Patients were excluded if they (1) were missing clinical data, (2) were pregnant or lactating, (3) or had a coinfection with another virus. Eventually, the study included 184 outpatients with influenza-B-related pneumonia and 180 influenza B outpatients without pneumonia as controls. Influenza-B-positive nasal swabs were immediately placed in tubes containing virus transport medium and stored at -80°C until further analysis.

### Data collection

The following data were collected through electronic medical records: demographic characteristics (age, gender), underlying conditions (hypertension, diabetes, coronary heart disease, cerebrovascular disease, chronic lung disease, chronic renal disease, chronic liver disease, autoimmune diseases, malignancies and chemotherapy, organ transplantation, pregnancy), clinical manifestation and complications (fever, sore throat, cough, expectoration, rhinorrhoea, dyspnoea, headache, muscle soreness, gastrointestinal symptoms, chest pain, confusion, time of symptom onset, complications, antiviral treatment, admission to hospital, days since a negative test, death), laboratory results (leucocyte and differential cell counts, hepatic function, blood creatinine, C-reactive protein), and thoracic CT findings.

### RNA extraction, gene amplification, and sequencing

Viral RNA was extracted from all samples using a QIAamp Viral RNA Mini Kit (Cat. No. 52904, QIAGEN, Germany) according to the instructions provided by the manufacturer. Reverse transcription polymerase chain reaction (RT-PCR) was performed using a commercial kit (Cat. No. 18080051, Invitrogen, USA) using the extracted RNA as a template. A universal primer (5’-AGCAAAAGCAGG-3’) was used for reverse transcription. Total RNA (8 µl), universal primer (4 µl), and 10 mM dNTP (1 µl) were added to a reverse transcription tube on ice, incubated at 65°C for 5 min, and then chilled on ice again for at least 1 min. Thereafter, 10× RT buffer (2 µl), 25 mM MgCl_2_ (4 µl), 0.1 MDTT (2 µl), RNase inhibitor (1 µl of 40 U/µL), and Super-Script® III reverse transcriptase (1 µl of 200 U/µL) were added to the tube, which was then incubated at 50°C for 50 min, followed by 85°C for 5 min. RNase H (1 µl) was added to each tube, and the tubes were then incubated for 20 min at 37°C after chilling on ice and brief centrifugation. The complementary DNAs (cDNAs) produced by reverse transcription were stored at -20°C until use. For identification of influenza A and B viruses by quantitative polymerase chain reaction (qPCR), we used specific primers for amplification of the matrix gene. For influenza A(H1N1)pdm09 and H3N2, we used the forward primers 5’-ACATTCGAAGCA ACTGGAAA-3’ and 5’-ACCCTCAGTGTGATGGCTTCCAAA-3’ and the reverse primers 5’-GTRTTRCAATCGTGGACTGG-3’ and 5’-TAAGGGAGGCATAATCCGGCACAT-3’). For influenza B viruses we used the forward primer 5’-AGACCAGAGGGAAACTATGCCC-3’ and the reverse primer 5’-TCCGGATGTAACAGGTCTGACTT-3’. The full-length HA and NA genes of influenza B virus were amplified by PCR using high-fidelity thermostable DNA polymerase (Cat. No. 11304011, Invitrogen, USA). The specific primers were provided by Shanghai BioGerm Medical Technology Co., LTD, and their sequences are shown in Table 1. The PCR amplification system included the cDNA template (4 µl), autoclaved distilled water (12.1 µl), 10× High Fidelity PCR Buffer (2 µl), 50 mM MgSO_4_ (0.6 µl), 10 mM dNTP mix (0.4 µl), 10 µM forward primer (0.4 µl), 10 µM reverse primer (0.4 µl), and Platinum® Taq DNA Polymerase High Fidelity (0.1 µl of 5 U/µL). The amplification conditions were 50℃ for 30 min and 94℃ for 5 min, followed by 35 cycles of 94°C for 30 s, 56℃ for 30 s, and 72°C for 1.5 min, and then 72°C for 7 min. All PCR products, markers, and the negative control were analyzed by electrophoresis in 1.5% agarose gels stained with ethidium bromide and visualized using a Molecular Imager Gel Doc XR System (Bio-Rad 170–8170, Hercules, CA, USA).

The HA and NA gene sequences of the samples from this study were determined and deposited in the NCBI database with accession numbers OR145863-145903 and OR150157-150197.

**Table 1:** Specific primers used for amplification of the HA and NA genes of influenza B virus

Primer	Sequence (5’–3’)
HA-1F	TGTAAAACGACGGCCAGTAGCAGAAGCRKWGC
HA-1R	CAGGAAACAGCTATGACCYTCRTCTTCACTGTTTATTATTCC
HA-2F	TGTAAAACGACGGCCAGTGCMATGGATGAACTCCAYAAC
HA-2R	CAGGAAACAGCTATGACCAGTAGTAACAAGAGCATTTTTC
NA-1F	TGTAAAACGACGGCCAGTAGCAGAAGCAGAGC
NA-1R	CAGGAAACAGCTATGACCCRCATGTGCATTCYTCAGTRTG
NA-2F	TGTAAAACGACGGCCAGTGTGCCTGCAAYTGCATCGG
NA-2R	CAGGAAACAGCTATGACCTGTAGTAACAAGAGCATT

### Phylogenetic analysis

For analysis of molecular evolution, nucleotide sequences of reference strains of influenza B virus were retrieved from the National Center for Biotechnology Information (NCBI) resource database. PKUPH (Beijing) isolates were selected randomly. A total of 28 HA and NA sequences from NCBI and 41 HA and NA sequences from our study were used for phylogenetic analysis. Multiple sequence alignments were made using ClustalW 2.10, and maximum-likelihood trees were constructed in MEGA 11.0 with gamma-distributed rates. Phylogenetic analysis was performed using the Hasegawa-Kishino-Yano model for the HA gene segment and the Tamura 3-parameter model for the NA gene segment, which were chosen as the best fit for the data. For both trees, 1000 replications of bootstrap analysis were performed to assess the reliability of branching.

### Statistical analysis

Categorical variables are reported as counts (percentage). Frequency comparisons were made using the χ^2^ test or Fisher’s exact test. Continuous variables are reported as the mean ± SD or median (interquartile range). Two-group comparisons of normally distributed data were performed using the independent-samples *t*-test. Non-normally distributed data were analyzed with the U-test. Results with *P* < 0.05 were considered statistically significant.

## Results

### Demographic and clinical characteristics of the study patients

A total of 1569 outpatients tested positive for influenza B virus, 11.7% (184/1569) of whom developed pneumonia. The characteristics of the 364 patients included in the study are shown in Table 2. Patients with pneumonia were slightly older than those without pneumonia (median age, 35.0 vs. 32.5). There was a slight majority of males in the two groups: 51.1% and 56.1%, respectively. In the pneumonia group, 19.0% (35/184) of patients had underlying diseases, which was significantly higher than in the non-pneumonia group (7.2%; 13/180).

Fever (100%; 184/184), cough (78.3%; 144/184), and sore throat (65.2%; 120/184) were the most common symptoms in the pneumonia group. In the non-pneumonia group, fever (100%; 180/180), cough (69.4%; 125/180), and sore throat (60.6%; 109/180) were also the most common symptoms. No significant differences were observed in clinical symptoms between the two groups. The median day on which the virus was no longer detected was 3 days (1–9 days) for the study group and 4 days (2–5 days) for the control group. However, these data were obtained from only 108 patients (72 with pneumonia and 36 without pneumonia) because virus positivity was not monitored in some patients with mild symptoms (Table 2).

In the pneumonia group, acute respiratory distress syndrome (ARDS) was reported in 2.7% (5/184) of the patients, acute kidney injury (AKI) in 4.9% (9/184), and shock in 1.6% (3/184). In the non-pneumonia group, ARDS, AKI, and shock were reported in 1.1%, 1.7%, and 0.6% of the patients, respectively. The incidence of hospitalization and mortality in the pneumonia group was higher than that in non-pneumonia group (2.7% vs. 1.1%; 0.5% vs. 0%) (Table 2).

**Table 2:** Demographic and clinical features of influenza B virus infection in patients with and without pneumonia

	With pneumonia (n = 184)	Without pneumonia (n = 180)	*P*-value
Male sex (%)	94.0 (51.1)	101.0 (56.1)	> 0.05
Age, median years (range)	35.0 (18.0–96.0)	32.5 (16.0–65.0)	> 0.05
underlying disease (%)	35.0 (19.0)	13.0 (7.2)	**< 0.05**
Onset time, hours (range)	24.0 (0.5–120.0)	24.0 (0.7–120.0)	> 0.05
Max temperature (°C)	38.4 (37.2–40.0)	38.4 (37.2–40.0)	> 0.05
Headache (%)	86.0 (46.7)	99.0 (55.0)	> 0.05
Muscle soreness (%)	88.0 (47.8)	87.0 (48.3)	> 0.05
Rhinorrhoea (%)	95.0 (51.6)	93.0 (51.7)	> 0.05
Sore throat (%)	120.0 (65.2)	109.0 (60.6)	> 0.05
Cough (%)	144.0 (78.3)	125.0 (69.4)	> 0.05
Expectoration (%)	81.0 (44.0)	77.0 (42.8)	> 0.05
Gastrointestinal symptoms (%)	45.0 (24.5)	27.0 (15.0)	> 0.05
Confusion (%)	3.0 (1.6)	1.0 (0.6)	> 0.05
Virus detection turning negative, days (range)*	3.0 (1.0–9.0)	4.0 (2.0–5.0)	> 0.05
Complications (%)			
ARDS	5.0 (2.7)	2.0 (1.1)	> 0.05
Acute kidney injury	9.0 (4.9)	3.0 (1.7)	> 0.05
Shock	3.0 (1.6)	1.0 (0.6)	> 0.05
Hospitalization (%)	5.0 (2.7)	2.0 (1.1)	> 0.05
Death (%)	1.0 (0.5)	0.0 (0.0)	> 0.05

ARDS: acute respiratory distress syndrome

*Data were available for 108 patients (72 with pneumonia; 36 without pneumonia)

### Laboratory findings

As shown in Table 3, lymphocyte counts were slightly lower than normal in both groups (median: 1.1 × 10^9^/L and 1.0 × 10^9^/L), and the median values for other laboratory results were within normal limits.

**Table 3:** Comparison of laboratory test results for influenza B virus infection in patients with and without pneumonia

	With pneumonia (n = 184)	Without pneumonia (n = 180)	*P-* value
WBC (× 10^9^/L)	5.5 (0.4-105.3)	5.4 (2.0-12.1)	> 0.05
Neutrophils (%)	69.4 (1.6–95.1)	69.5 (32.0–88.0)	> 0.05
Neutrophils (× 10^9^/L)	3.8 (0.0-23.5)	3.7 (0.9–8.8)	> 0.05
Lymphocytes (%)	19.4 (2.8–92.5)	19.0 (4.5–56.0)	> 0.05
Lymphocytes (×10^9^/L)	1.1 (0.2-4.0)	1.0 (0.2–3.5)	> 0.05
Monocytes (%)	8.9 (2.0-21.4)	10.2 (3.0-18.8)	> 0.05
Monocytes (× 10^9^/L)	0.5 (0.1–1.4)	0.5 (0.1–1.4)	> 0.05
Platelets (× 10^9^/L)	196.0 (9.0-400.0)	196.0 (51.0-334.0)	> 0.05
C-reactive protein (mg/L)	7.6 (0.4-244.5)	6.2 (0.3–67.4)	> 0.05
Alanine aminotransferase (U/L)	17.0 (7.0-125.0)	19.5 (5.0-128.0)	> 0.05
Aspartate aminotransferase (U/L)	20.0 (8.0-139.0)	20.0 (11.0-210.0)	> 0.05
Albumin (g/L)	45.8 (26.3–51.5)	46.9 (39.3–54.5)	> 0.05
Lactate dehydrogenase (U/L)	175.0 (91.0-839.0)	168.5 (105.0-289.0)	> 0.05
Creatine kinase (U/L)	80.0 (28.0-2866.0)	76.0 (18.0-215.0)	> 0.05
Blood creatinine (µmol/L)	74.0 (44.0-222.0)	71.5 (41.0-114.0)	> 0.05

### Treatment

In the pneumonia group, 98.4% (181/184) received antiviral therapy. In the non-pneumonia group, 93.4% (169/180) received antiviral therapy. In the two groups, 74.0% (134/181) and 75.7% (128/169), respectively, received antiviral treatment within 48 hours. The most frequently prescribed anti-influenza medication was a neuraminidase inhibitor (NAI). Among the 184 patients with pneumonia, 94.6% (174/184) received antibiotics. Fluoroquinolones and cephalosporins were the most frequently used antibiotics. Nearly half of the patients (47.8%, 86/180) in the non-pneumonia group received antibiotics.

### Epidemic characteristics of seasonal influenza

To examine the distribution of influenza virus in northern China from May 2021 to June 2022, we collected the weekly data from the Chinese National Influenza Center. B/Victoria (BV) was the dominant strain. A small number of influenza B virus samples were untyped. Influenza A virus and B/ Yamagata (BY) were detected occasionally. In terms of time distribution, BV-positive samples were detected every week from May 2021 to June 2022. The prevalence of BV was low from May to June 2021. The number of positive samples increased rapidly after October 2021, reached a peak in January 2022, decreased briefly in February, then rebounded again, began to decrease significantly in April, and maintained a low level in May. All of the influenza B isolates in this study were identified as lineage BV by RT-PCR.

### Phylogenetic analysis of the HA and NA genes of influenza B viruses

**Fig. 1:**
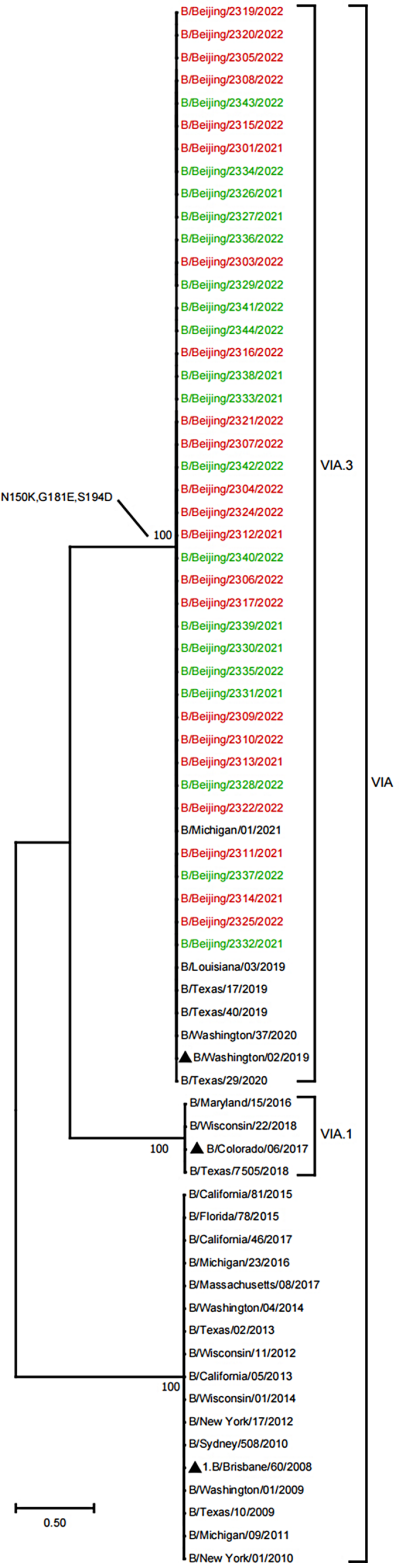
Phylogenetic tree based on HA nucleotide sequences of B/Victoria isolates from 2021 to 2022. The tree was constructed by the maximum-likelihood method with gamma-distributed rates and the Hasegawa-Kishino-Yano model, which was chosen as the best fit for our data using MEGA software version 11.0,. The reliability of the branching was assessed by bootstrap analysis with 1000 replications. The vaccine strain is indicated by “▲”. Red represents viral strains from outpatients with pneumonia, and green represents viral strains from outpatients without pneumonia

**Fig. 2:**
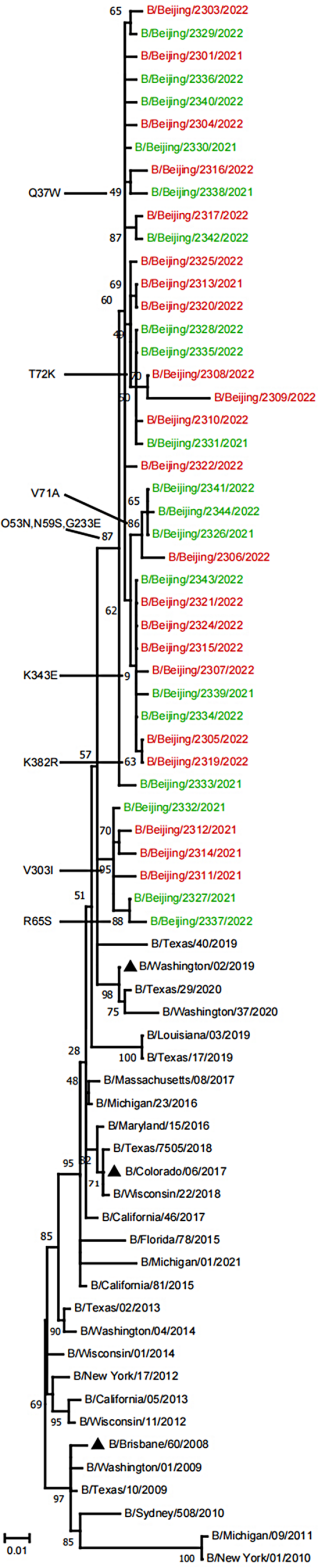
Phylogenetic tree based on NA nucleotide sequences of B/Victoria isolates from 2021 to 2022. The tree was constructed by the maximum-likelihood method with gamma-distributed rates and the Tamura 3-parameter model, which was chosen as the best fit for our data using MEGA software version 11.0. The reliability of the branching was assessed by bootstrap analysis with 1000 replications. The vaccine strain is indicated by “▲”. Red represents viral strains from outpatients with pneumonia, and green represents viral strains from outpatients without pneumonia

In this study, 22 outpatients with pneumonia and 19 outpatients without pneumonia were selected randomly for full-length amplification and sequencing of HA and NA genes. To analyze the evolutionary relationship of 41 BV viruses from PKUPH (Beijing), we compared HA and NA gene sequences with other BV sequences from the NCBI database (Figs. 1 and 2). Phylogenetic analysis based on HA sequences indicated that all of the Beijing strains belonged to clade 1A.3, the clade that includes vaccine strain B/Washington/02/2019 (Fig. 1). All 41 viruses had a triple deletion of amino acids 162–164 in HA(3DEL). When analyzing NA sequences, a similar evolutionary pattern was observed (Fig. 2). The results suggest that all of these viruses belong to the same evolutionary lineage as vaccine strain B/Washington/02/2019. The evolutionary pattern of virus strains was the same in the pneumonia group and the non-pneumonia group.

### Comparison of HA and NA gene sequences of influenza B viruses

The HA gene sequence of 1749 bp and the NA gene sequence of 1401 bp encoding 583 and 467 amino acids, respectively, were determined for 41 isolates.

During the 2021–2022 influenza season in Beijing, the nucleotide sequence identity of HA was 98.5–100%, and the amino acid sequence identity was 97.9–100% among the influenza B virus strains from PKUPH. The nucleotide sequence identity of NA was 98.1–100%, and the amino acid sequence identity was 95–100.0% among the influenza B strains from PKUPH. Compared with vaccine strain B/Colorado/06/2017, the BV isolates from this study shared 50.5–51.1% and 98.2–99.4% nucleotide sequence identity and 34.1–34.9% and 96.6–99.1% amino acid sequence identity in their HA and NA genes, respectively. Compared with vaccine strain B/Washington/02/2019, the BV isolates from this study shared 98.5–99.0% and 98.3–99.4% nucleotide sequence identity and 97.9–98.6% and 97.0-99.6% amino acid sequence identity in their HA and NA genes, respectively (Tables 4 and 5). The above results indicate that all of the isolates in this study were similar to vaccine strain B/Washington/02/2019.

No statistically significant differences were found in the HA and NA nucleotide or amino acid sequences of influenza B viruses in patients with and without pneumonia.

**Table 4:** HA and NA nucleotide sequence identity between BV isolates and vaccine strains

		Among isolates	B/Colorado/06/2017	B/Washington/02/2019
HA	With pneumonia	98.6–99.9%	50.7–50.8%	98.5–99.0%
	Without pneumonia	98.5–100%	50.5–51.1%	98.5–98.9%
NA	With pneumonia	98.1–100%	98.2–99.2%	98.3–99.3%
	Without pneumonia	98.1–100%	98.9–99.4%	99.0-99.4%

**Table 5:** HA and NA amino acid sequence identity between BV isolates and vaccine strains

		Among isolates	B/Colorado/06/2017	B/Washington/02/2019
HA	With pneumonia	97.9–100%	34.1–34.8%	97.9–98.6%
	Without pneumonia	97.9–100%	34.1–34.9%	98.1–98.6%
NA	With pneumonia	96.4–100%	96.6–99.1%	97.0-99.6%
	Without pneumonia	95.9–100%	98.3–99.1%	98.7–99.6%

### Analysis of amino acid substitutions in HA and NA

As shown in Table 6, HA exhibited 13 key substitutions when compared with vaccine strain B/Washington/02/2019. H122Q, H122N, A127T, E128K, E128G, and A130T substitutions were identified in the 120-loop. P144L, T147S, G149E, and N150K substitutions were identified in the 150-loop. K164Q and A166T substitutions were identified in the 160-loop. S194D, E195K, and K200R substitutions were identified in the 190-helix. All strains contained the N150K, G181E, and S194D mutations.

It was observed that NA exhibited 16 substitutions when compared with vaccine strain and B/Washington/02/2019 (Table 6). None of the mutations in NA were in the active site or surrounding residues.

More substitutions were found in isolates from the pneumonia group than in those from the non-pneumonia group. The co-occurring mutations H122Q, A127T, P144L, N150K, G181E, S194D, and K200R in HA and D53N, N59S, and G233E in NA were detected in 78.0% (32/41) of the isolates, including 18 from the pneumonia group and 14 from the non-pneumonia group. Over 50% (17/32) of the patients with co-occurring mutations had underlying diseases.

**Table 6:** Amino acid mutations in the HA and NA proteins

HA	Number of substitutions		NA	Number of substitutions	
	With pneumonia	Without pneumonia		With pneumonia	Without pneumonia
H122Q	19	17	D53N	19	16
H122N	1	0	N59S	19	16
A127T	19	17	R65S	0	2
E128K	1	0	V71A	2	0
E128G	0	1	T72K	6	3
A130T	1	0	T106A	1	0
P144L	19	17	T106I	1	0
T147S	1	0	A175T	1	0
G149E	0	1	G233E	19	15
N150K	22	19	I240M	1	0
K164Q	1	2	V271I	0	1
A166T	1	2	V303I	4	3
G181E	22	19	K343E	11	9
S194D	22	19	Q371N	1	1
E195K	0	1	G378E	1	0
K200R	19	15	K382R	2	0
			D463V	2	0

## Discussion

Even after decades of significant advances in medical technology, influenza causes substantial hospitalization and mortality [11, 12]. Influenza complicated with pneumonia is the primary cause of hospitalization [8], but data on outpatients with influenza-B-related pneumonia has been limited. Therefore, we conducted an analysis of the clinical characteristics of patients with influenza-B-related pneumonia during the 2021–2022 influenza season and investigated the molecular epidemiology and evolutionary patterns of influenza B virus.

The incidence of influenza-B-associated pneumonia in outpatients in this study was 11.7%. In previous studies, the incidence of pneumonia in inpatients with influenza B was reported to be 27–49% [13, 14]. The median age of patients in the pneumonia group was 35 years, and only 5.4% (10/184) of the patients were older than 65 years. In contrast, Fu et al. [9] reported that the median age of hospitalized patients with influenza-B-associated pneumonia was 62 (53–74) years. This difference might be attributable to two factors. First, patients over the age of 65 have a higher risk of severe influenza, and second, young patients had more social activities that increase the risk of influenza virus infection, especially during the COVID-19 pandemic. Previous studies have shown that influenza-B-associated pneumonia mainly affects patients with underlying conditions [9, 15], and in our study we also found the pneumonia group with underlying disease was significantly higher than the non-pneumonia group (19.0% vs. 7.2%). Dai et al. [13] analyzed the risk factors for influenza B virus − associated pneumonia in adult patients and found that chronic pulmonary diseases were an independent risk factor because of structural damage to the bronchial tubes, the use of steroids, and a lack of innate antiviral immunity.

The most common clinical symptoms in hospitalized patients with influenza-B-related pneumonia were fever and cough [9, 13, 16]. Patients with influenza B with or without pneumonia presented with fever and cough in our study, and no significant differences were observed in clinical symptoms between the two groups.

Regarding complications, outpatients in the pneumonia group had ARDS (2.7%), AKI (4.9%), and shock (1.6%). Complications of inpatients with influenza-B-related pneumonia varied in different reports. Chen et al. described complications in 386 cases as follows: ARDS, 36.3%; AKI, 3.1%; and shock, 4.4% [16]. This suggests that hospitalized patients have a higher risk of influenza-related complications than outpatients. In our study, the hospitalization rate was 2.7%, and the mortality rate was 0.5% in the pneumonia group.

Lymphocyte counts were somewhat lower than normal in these two groups, with no significant difference, which is consistent with previous literature [13].

No significant differences were observed in clinical symptoms or laboratory results between outpatients with and without pneumonia, so testing for influenza virus seems to be a good choice.

Previous reports have suggested that patients with influenza B could benefit from early NAI treatment within 48 h of disease onset [17–19]. Out of 384 patients, 350 (91.1%) received antiviral treatment, 97.7% (342/350) of which received NAI, and about 75% (262/350) of the patients received NAI within 48 h. Influenza with pneumonia is fairly common [8], and in severe cases, patients can benefit from antiviral therapy even when the time since onset has exceeded 48 h [20, 21]. The findings of a large study showed that early NAI treatment reduced the risk of death in patients with influenza-B-related pneumonia [14]. In the pneumonia group, 26.0% (47/181) of the patients received NAIs more than 48 h after disease onset, with the average time being 72 h (50–120 h). It is advised that people with influenza-like symptoms should consult a doctor in the early stage of illness.

Among adult community-acquired pneumonia (CAP) patients in China, viruses account for 15.0-34.9% of the cases, with influenza viruses being the most frequent [22]. Influenza virus infection also predisposes an individual to bacterial secondary infections [23]. Therefore, antibiotics are used frequently in patients with influenza pneumonia [9, 13, 21]. The same was true in our study, in which antibiotic use was 94.6% in the pneumonia group. Fluoroquinolones and cephalosporins were the most frequently used empiric antibiotics.

Seasonal influenza virus circulation has declined globally since the time before the COVID-19 pandemic [24, 25], but it has increased in China since 2021. Due to the various prevention and control measures used in different countries, the epidemic levels and dominant strains of influenza viruses differ from those before COVID-19. In China, the 2021–2022 influenza season was the first season following the COVID-19 pandemic and was dominated by influenza B viruses [26]. Analysis of the epidemiology and antigenic and genetic characteristics of influenza B viruses during the 2021–2022 influenza season showed that, since COVID-19, there has been a decrease in the diversity of subtypes of influenza viruses co-circulating in China, and almost all of the current strains belong to the B/Victoria lineage [27, 28]. In agreement with previous studies [26, 29], most of the influenza B viruses circulating in 2021–2022 in Beijing belonged to clade 1A.3, whose members contain 3DEL.

Positive selection of mutations in certain domains influence influenza B virus evolution, as has been seen in four epitopes of HA: the 120-loop (aa 116–137), the 150-loop (aa 141–150), the 160-loop (aa 162–167), and the 190-helix (aa 194–202) [30, 31]. One of the most frequently mutated regions of HA is the 120-loop epitope, which plays a crucial role in antigenicity [32]. H122Q and A127T were reported previously [26]. H122N, E128K, E128G, and A130T substitutions were observed when compared to the vaccine strain B/Washington/02/2019, indicating a potential change in the antigenicity of the Beijing strains. The epitope 150-loop is also important, especially for the BY lineage [31]. Four substitutions were identified in the 150 loop. The 160-loop of influenza B virus is an especially variable region of HA, since insertions, deletions, and single amino acid substitutions have all been detected in this region in field isolates. K164Q and A166T substitutions have been identified in the 160-loop [33, 34]. The G181E mutation was detected in all of the Beijing strains. This mutation might provide influenza B viruses with the ability to survive for long periods without antigenic shifts [35]. S194D, N195E, N195K, and K200R were identified in the 190-helix, which is involved in receptor binding sites [36]. In the Beijing strains, 13 mutations were located in four different epitopes, some of which might affect the efficacy of vaccines.

In the NA protein, the residues R116, D149, R150, R223, E275, R292, R374, and Y409 form the catalytic sites and E117, R154, W177, S178, D197, I221, E226, H273, E276, N294, and E428 form auxiliary sites [37, 38]. The amino acid substitutions E105K, P139S, G140R, D197N, and H273Y in the NA gene can lead to reduced sensitivity to NAI [39–41]. None of these mutations were found in any of the Beijing strains.

Events such as recombination and infection of new hosts can disrupt the balance between HA and NA and promote or inhibit the ability of the virus to replicate or adapt *in vitro* [42]. Previously, we reported that co-mutation might increase the fitness of the virus in a new environment or host [43]. Co-mutations of HA and NA were also detected in the present study, and 56.3% (18/32) of the patients with influenza-B-associated pneumonia were infected with viruses with co-mutations, and this will be a topic of further investigations.

## Conclusions

In this study, we investigated the clinical characteristics of outpatients with influenza-B-associated pneumonia and the molecular evolution of influenza B virus in Beijing during the 2021–2022 influenza season. Influenza outpatients with underlying diseases were more likely to develop pneumonia. No significant differences were observed in clinical symptoms or laboratory results between the two groups, and we conclude that influenza virus detection seems to be a good choice. The HA protein exhibited 13 amino acid substitutions when compared with the vaccine strain B/Washington/02/2019, suggesting that vaccines might not provide adequate protection against these strains and that constant monitoring of influenza virus strains is necessary. Furthermore, co-occurring mutations in the HA or NA proteins, including H122Q, A127T, P144L, N150K, G181E, S194D, and K200R in HA and D53N, N59S, and G233E in NA were detected in 56.3% (18/32) of the patients with influenza-B-associated pneumonia. Further investigations of the significance of these co-mutations are needed.

### Electronic Supplementary Material

Below is the link to the electronic supplementary material

Supplementary Material 1

Supplementary Material 2

Supplementary Material 3
