# Supplementary material for: Clinical characteristics of outpatients with influenza-B-associated pneumonia and molecular evolution of influenza B virus in Beijing, China, during the 2021–2022 influenza season
Source: Arch Virol. 2024 Jan 18;169(2):30. doi: 10.1007/s00705-023-05957-6 (PMC10794387; doi:10.1007/s00705-023-05957-6)
Supplement: Supplementary file 2 — Supplementary Material 2 [file 705_2023_5957_MOESM2_ESM.docx]

LOCUS OR145863 1749 bp cRNA linear VRL 20-JUN-2023

DEFINITION Influenza B virus (B/Beijing/2301/2022) segment 4 hemagglutinin

(HA) gene, complete cds.

ACCESSION OR145863

VERSION OR145863

KEYWORDS .

SOURCE Influenza B virus

ORGANISM Influenza B virus

Viruses; Riboviria; Orthornavirae; Negarnaviricota;

Polyploviricotina; Insthoviricetes; Articulavirales;

Orthomyxoviridae; Betainfluenzavirus; Betainfluenzavirus

influenzae.

REFERENCE 1 (bases 1 to 1749)

AUTHORS Wang,Y.

TITLE Direct Submission

JOURNAL Submitted (20-JUN-2023) Department of Infectious Diseases, Peking

University People's Hospital, Xizhimen South Street, Beijing

100044, China

COMMENT ##Assembly-Data-START##

Sequencing Technology :: Sanger dideoxy sequencing

##Assembly-Data-END##

FEATURES Location/Qualifiers

source 1..1749

/organism="Influenza B virus"

/mol_type="viral cRNA"

/strain="B/Beijing/2301/2022"

/isolate="2301"

/isolation_source="nasal swab"

/host="Homo sapiens"

/db_xref="taxon:11520"

/segment="4"

/country="China: Beijing"

/collection_date="30-Aug-2022"

gene 1..1749

/gene="HA"

CDS 1..1749

/gene="HA"

/function="receptor binding and fusion protein"

/codon_start=1

/product="hemagglutinin"

/protein_id="WJE87834"

/translation="MKAIIVLLMVVTSNADRICTGITSSNSPHVVKTATQGEVNVTGV

IPLTTTPTKSHFANLKGTETRGKLCPKCLNCTDLDVALGRPKCTGKIPSARVSILHEV

RPVTSGCFPIMHDRTKIRQLPNLLRGYEHVRLSTQNVINTEDAPGGPYEIGTSGSCLN

ITNGKGFFATMAWAVPKNKTATNPLTIEVPYICTEEEDQITVWGFHSDDETQMARLYG

DSKPQKFTSSANGVTTHYVSQIGGFPNQTEDGGLPQSGRIVVDYMVQKSGKTGTITYQ

RGILLPQKVWCASGKSKVIKGSLPLIGEADCLHEKYGGLNKSKPYYTGEHAKAIGNCP

IWVKTPLKLANGTKYRPPAKLLKERGFFGAIAGFLEGGWEGMIAGWHGYTSHGAHGVA

VAADLKSTQEAINKITKNLNSLSELEVKNLQRLSGAMDELHNEILELDEKVDDLRADT

ISSQIELAVLLSNEGIINSEDEHLLALERKLKKMLGPSAVEIGNGCFETKHKCNQTCL

DRIAAGTFDAGEFSLPTFDSLNITAASLNDDGLDNHTILLYYSTAASSLAVTLMIAIF

VVYMVSRDNVSCSICL"

ORIGIN

1 atgaaggcaa taattgtact actcatggta gtaacatcca atgcagatcg aatctgcact

61 gggataacat cgtcaaactc accacatgtc gtcaaaactg ctactcaagg ggaggtcaat

121 gtgaccggtg taataccact gacaacaaca cccaccaaat ctcattttgc aaatctcaaa

181 ggaacagaaa ccagggggaa actatgccca aaatgcctaa actgcacaga tctggatgta

241 gccttgggca gaccaaaatg cacagggaaa ataccctctg caagggtttc aatactccat

301 gaagtcagac ctgttacatc tgggtgcttt cctataatgc atgatagaac aaaaattaga

361 cagctgccta accttctccg aggatacgaa catgtcaggt tatcaactca aaacgttatc

421 aatacagaag atgcaccagg aggaccctac gaaattggaa cctcagggtc ttgcctcaac

481 attaccaatg gaaaaggatt cttcgcaaca atggcttggg ccgtcccaaa aaacaaaaca

541 gcaacaaatc cattaacaat agaagtacca tacatttgta cagaagaaga agaccaaatt

601 accgtttggg ggttccactc tgacgatgag acccaaatgg caaggctcta tggggattca

661 aagccccaga agttcacctc atctgccaac ggagtgacca cacattacgt ctcacagatt

721 ggtggcttcc caaatcaaac agaagacgga ggactaccac aaagtggcag aattgttgtt

781 gattacatgg tgcaaaaatc tggaaaaaca ggaacaatta cctatcaaag aggtatttta

841 ttgcctcaaa aggtgtggtg cgcaagtggc aagagcaagg taataaaagg atccttgccc

901 ttaattggag aagcagattg cctccatgaa aaatacggtg gattaaacaa aagcaagcct

961 tactacacag gggaacatgc aaaggccata ggaaattgcc caatatgggt gaaaacaccc

1021 ttgaagctgg ccaatggaac caaatataga cctcctgcaa aactattaaa ggaaagaggt

1081 ttcttcggag ccattgctgg tttcttagag ggaggatggg aaggaatgat tgcaggttgg

1141 cacggataca catcccatgg ggcacatgga gtagcagtgg cagctgacct taagagcact

1201 caagaggcca taaacaagat aacaaaaaat ctcaactctt tgagtgagct ggaagtaaag

1261 aatcttcaaa gactaagcgg tgccatggat gaactccaca acgaaatact agaactagat

1321 gagaaagtgg atgatctcag agctgataca ataagctcac aaatagaact cgcagtcctg

1381 ctttccaatg aaggaataat aaacagtgaa gatgaacatc tcttggcgct tgaaagaaag

1441 ctgaagaaaa tgctgggccc ctctgctgta gagataggga atggatgctt tgaaaccaaa

1501 cacaagtgca accagacctg tctcgacaga atagctgctg gtacctttga tgcaggagaa

1561 ttttctctcc ccacctttga ttcactgaat attactgctg catctttaaa tgacgatgga

1621 ttggacaatc atactatact gctttactac tcaactgctg cctccagttt ggctgtaaca

1681 ctgatgatag ctatctttgt tgtttatatg gtctccagag acaatgtttc ttgctccatt

1741 tgtctataa

//

LOCUS OR145864 1749 bp cRNA linear VRL 20-JUN-2023

DEFINITION Influenza B virus (B/Beijing/2303/2022) segment 4 hemagglutinin

(HA) gene, complete cds.

ACCESSION OR145864

VERSION OR145864

KEYWORDS .

SOURCE Influenza B virus

ORGANISM Influenza B virus

Viruses; Riboviria; Orthornavirae; Negarnaviricota;

Polyploviricotina; Insthoviricetes; Articulavirales;

Orthomyxoviridae; Betainfluenzavirus; Betainfluenzavirus

influenzae.

REFERENCE 1 (bases 1 to 1749)

AUTHORS Wang,Y.

TITLE Direct Submission

JOURNAL Submitted (20-JUN-2023) Department of Infectious Diseases, Peking

University People's Hospital, Xizhimen South Street, Beijing

100044, China

COMMENT ##Assembly-Data-START##

Sequencing Technology :: Sanger dideoxy sequencing

##Assembly-Data-END##

FEATURES Location/Qualifiers

source 1..1749

/organism="Influenza B virus"

/mol_type="viral cRNA"

/strain="B/Beijing/2303/2022"

/isolate="2303"

/isolation_source="nasal swab"

/host="Homo sapiens"

/db_xref="taxon:11520"

/segment="4"

/country="China: Beijing"

/collection_date="30-Aug-2022"

gene 1..1749

/gene="HA"

CDS 1..1749

/gene="HA"

/function="receptor binding and fusion protein"

/codon_start=1

/product="hemagglutinin"

/protein_id="WJE87835"

/translation="MKAIIVLLMVVTSNADRICTGITSSNSPHVVKTATQGEVNVTGV

IPLTTTPTKSHFANLKGTETRGKLCPKCLNCTDLDVALGRPKCTGKIPSARVSILHEV

RPVTSGCFPIMHDRTKIRQLPNLLRGYEHVRLSTQNVINTEDAPGGPYEIGTSGSCLN

ITNGKGFFATMAWAVPKNKTATNPLTIEVPYICTEEEDQITVWGFHSDDETQMARLYG

DSKPQKFTSSANGVTTHYVSQIGGFPNQTEDGGLPQSGRIVVDYMVQKSGKTGTITYQ

RGILLPQKVWCASGKSKVIKGSLPLIGEADCLHEKYGGLNKSKPYYTGEHAKAIGNCP

IWVKTPLKLANGTKYRPPAKLLKERGFFGAIAGFLEGGWEGMIAGWHGYTSHGAHGVA

VAADLKSTQEAINKITKNLNSLSELEVKNLQRLSGAMDELHNEILELDEKVDDLRADT

ISSQIELAVLLSNEGIINSEDEHLLALERKLKKMLGPSAVEIGNGCFETKHKCNQTCL

DRIAAGTFDAGEFSLPTFDSLNITAASLNDDGLDNHTILLYYSTAASSLAVTLMIAIF

VVYMVSRDNVSCSICL"

ORIGIN

1 atgaaggcaa taattgtact actcatggta gtaacatcca atgcagatcg aatctgcact

61 gggataacat cgtcaaactc accacatgtc gtcaaaactg ctactcaagg ggaggtcaat

121 gtgaccggtg taataccact gacaacaaca cccaccaaat ctcattttgc aaatctcaaa

181 ggaacagaaa ccagggggaa actatgccca aaatgcctaa actgcacaga tctggatgta

241 gccttgggca gaccaaaatg cacagggaaa ataccctctg caagggtttc aatactccat

301 gaagtcagac ctgttacatc tgggtgcttt cctataatgc atgatagaac aaaaattaga

361 cagctgccta accttctccg aggatacgaa catgtcaggt tatcaactca aaacgttatc

421 aatacagaag atgcaccagg aggaccctac gaaattggaa cctcagggtc ttgcctcaac

481 attaccaatg gaaaaggatt cttcgcaaca atggcttggg ccgtcccaaa aaacaaaaca

541 gcaacaaatc cattaacaat agaagtacca tacatttgta cagaagaaga agaccaaatt

601 accgtttggg ggttccactc tgacgacgag acccaaatgg caaggctcta tggggattca

661 aagccccaga agttcacctc atctgccaac ggagtgacca cacattacgt ctcacagatt

721 ggtggcttcc caaatcaaac agaagacgga ggactaccac aaagtggcag aattgttgtt

781 gattacatgg tgcaaaaatc tggaaaaaca ggaacaatta cctatcaaag aggtatttta

841 ttgcctcaaa aggtgtggtg cgcaagtggc aagagcaagg taataaaagg atccttgccc

901 ttaattggag aagcagattg ccttcatgaa aaatacggtg gattaaacaa aagcaagcct

961 tactacacag gggaacatgc aaaggccata ggaaattgcc caatatgggt gaaaacaccc

1021 ttgaagctgg ccaatggaac caaatataga cctcctgcaa aactattaaa ggaaagaggt

1081 ttcttcggag ccattgctgg tttcttagaa ggaggatggg aaggaatgat tgcaggttgg

1141 cacggataca catcccatgg ggcacatgga gtagcagtgg cagctgacct taagagcact

1201 caagaggcca taaacaagat aacaaaaaat ctcaactctt tgagtgagct ggaagtaaag

1261 aatcttcaaa gactaagcgg tgccatggat gaactccaca acgaaatact agaactagat

1321 gagaaagtgg atgatctcag ggctgataca ataagctcac aaatagaact cgcagtcctg

1381 ctttccaatg aaggaataat aaacagtgaa gatgaacatc tcttggcgct tgaaagaaag

1441 ctgaagaaaa tgctgggccc ctctgctgta gagataggga atggatgctt tgaaaccaaa

1501 cacaagtgca accagacctg tctcgacaga atagctgctg gtacctttga tgcaggagaa

1561 ttttctctcc ccacctttga ttcactgaat attactgctg catctttaaa tgacgatgga

1621 ttggacaatc atactatact gctttactac tcaactgctg cctccagttt ggctgtaaca

1681 ctgatgatag ctatctttgt tgtttatatg gtctccagag acaatgtttc ttgctccatt

1741 tgtctataa

//

LOCUS OR145865 1749 bp cRNA linear VRL 20-JUN-2023

DEFINITION Influenza B virus (B/Beijing/2304/2022) segment 4 hemagglutinin

(HA) gene, complete cds.

ACCESSION OR145865

VERSION OR145865

KEYWORDS .

SOURCE Influenza B virus

ORGANISM Influenza B virus

Viruses; Riboviria; Orthornavirae; Negarnaviricota;

Polyploviricotina; Insthoviricetes; Articulavirales;

Orthomyxoviridae; Betainfluenzavirus; Betainfluenzavirus

influenzae.

REFERENCE 1 (bases 1 to 1749)

AUTHORS Wang,Y.

TITLE Direct Submission

JOURNAL Submitted (20-JUN-2023) Department of Infectious Diseases, Peking

University People's Hospital, Xizhimen South Street, Beijing

100044, China

COMMENT ##Assembly-Data-START##

Sequencing Technology :: Sanger dideoxy sequencing

##Assembly-Data-END##

FEATURES Location/Qualifiers

source 1..1749

/organism="Influenza B virus"

/mol_type="viral cRNA"

/strain="B/Beijing/2304/2022"

/isolate="2304"

/isolation_source="nasal swab"

/host="Homo sapiens"

/db_xref="taxon:11520"

/segment="4"

/country="China: Beijing"

/collection_date="30-Aug-2022"

gene 1..1749

/gene="HA"

CDS 1..1749

/gene="HA"

/function="receptor binding and fusion protein"

/codon_start=1

/product="hemagglutinin"

/protein_id="WJE87836"

/translation="MKAIIVLLMVVTSNADRICTGITSSNSPHVVKTATQGEVNVTGV

IPLTTTPTKSHFANLKGTETRGKLCPKCLNCTDLDVALGRPKCTGKIPSARVSILHEV

RPVTSGCFPIMHDRTKIRQLPNLLRGYEHVRLSTQNVINTEDAPGGPYEIGTSGSCLN

ITNGKGFFATMAWAVPKNKTATNPLTIEVPYICTEEEDQITVWGFHSDDETQMARLYG

DSKPQKFTSSANGVTTHYVSQIGGFPNQTEDGGLPQSGRIVVDYMVQKSGKTGTITYQ

RGILLPQKVWCASGKSKVIKGSLPLIGEADCLHEKYGGLNKSKPYYTGEHAKAIGNCP

IWVKTPLKLANGTKYRPPAKLLKERGFFGAIAGFLEGGWEGMIAGWHGYTSHGAHGVA

VAADLKSTQEAINKITKNLNSLSELEVKNLQRLSSAMDELHNEILELDEKVDDLRADT

ISSQIELAVLLSNEGIINSEDEHLLALERKLKKMLGPSAVEIGNGCFETKHKCNQTCL

DRIAAGTFDAGEFSLPTFDSLNITAASLNDDGLDNHTILLYYSTAASSLAVTLMIAIF

VVYMVSRDNVSCSICL"

ORIGIN

1 atgaaggcaa taattgtact actcatggta gtaacatcca atgcagatcg aatctgcact

61 gggataacat cgtcaaactc accacatgtc gtcaaaactg ctactcaagg ggaggtcaat

121 gtgaccggtg taataccact gacaacaaca cccaccaaat ctcattttgc aaatctcaaa

181 ggaacagaaa ccagggggaa actatgccca aaatgcctaa actgcacaga tctggatgta

241 gccttgggca gaccaaaatg cacagggaaa ataccctctg caagggtttc aatactccat

301 gaagtcagac ctgttacatc tgggtgcttt cctataatgc atgatagaac aaaaattaga

361 cagctgccta accttctccg aggatacgaa catgtcaggt tatcaactca aaacgttatc

421 aatacagaag atgcaccagg aggaccctac gaaattggaa cctcagggtc ttgcctcaac

481 attaccaatg gaaaaggatt cttcgcaaca atggcttggg ccgtcccaaa aaacaaaaca

541 gcaacaaatc cattaacaat agaagtacca tacatttgta cagaagaaga agaccaaatt

601 accgtttggg ggttccactc tgatgacgag acccaaatgg caaggctcta tggggattca

661 aagccccaga aattcacctc atctgccaac ggagtgacca cacattacgt ctcacagatt

721 ggtggcttcc caaatcaaac agaagacgga ggactaccac aaagtggcag aattgttgtt

781 gattacatgg tgcaaaaatc tggaaaaaca ggaacaatta cctatcaaag aggtatttta

841 ttgcctcaaa aggtgtggtg cgcaagtggc aagagcaagg taataaaagg atccttgccc

901 ttaattggag aagcagattg cctccatgaa aaatacggtg gattaaacaa aagcaagcct

961 tactacacag gggaacatgc aaaggccata gggaattgcc caatatgggt gaaaacaccc

1021 ttgaagctgg ccaatggaac caaatataga cctcctgcaa aactattaaa ggaaagaggt

1081 ttcttcggag ccattgctgg tttcttagag ggaggatggg aaggaatgat tgcaggttgg

1141 cacggataca catcccatgg ggcacatgga gtagcagtgg cagctgacct taagagcact

1201 caagaggcca taaacaagat aacaaaaaat ctcaactctt tgagtgagct ggaagtaaag

1261 aatcttcaaa gactaagcag tgccatggat gaactccaca acgaaatact agaactagat

1321 gagaaagtgg atgatctcag agctgataca ataagctcac aaatagaact cgcagtcctg

1381 ctttccaatg aaggaataat aaacagtgaa gatgaacatc tattggcgct tgaaagaaag

1441 ctgaagaaaa tgctgggccc ctctgctgta gagataggga atggatgctt tgaaaccaaa

1501 cacaagtgca accagacctg tctcgacaga atagctgctg gtacctttga tgcaggagaa

1561 ttttctctcc ccacctttga ttcactgaat attactgctg catctttaaa tgacgatgga

1621 ttggacaatc atactatact gctttactac tcaactgctg cctccagttt ggctgtaaca

1681 ctgatgatag ctatctttgt tgtttatatg gtctccagag acaatgtttc ttgctccatt

1741 tgtctataa

//

LOCUS OR145866 1749 bp cRNA linear VRL 20-JUN-2023

DEFINITION Influenza B virus (B/Beijing/2305/2022) segment 4 hemagglutinin

(HA) gene, complete cds.

ACCESSION OR145866

VERSION OR145866

KEYWORDS .

SOURCE Influenza B virus

ORGANISM Influenza B virus

Viruses; Riboviria; Orthornavirae; Negarnaviricota;

Polyploviricotina; Insthoviricetes; Articulavirales;

Orthomyxoviridae; Betainfluenzavirus; Betainfluenzavirus

influenzae.

REFERENCE 1 (bases 1 to 1749)

AUTHORS Wang,Y.

TITLE Direct Submission

JOURNAL Submitted (20-JUN-2023) Department of Infectious Diseases, Peking

University People's Hospital, Xizhimen South Street, Beijing

100044, China

COMMENT ##Assembly-Data-START##

Sequencing Technology :: Sanger dideoxy sequencing

##Assembly-Data-END##

FEATURES Location/Qualifiers

source 1..1749

/organism="Influenza B virus"

/mol_type="viral cRNA"

/strain="B/Beijing/2305/2022"

/isolate="2305"

/isolation_source="nasal swab"

/host="Homo sapiens"

/db_xref="taxon:11520"

/segment="4"

/country="China: Beijing"

/collection_date="30-Aug-2022"

gene 1..1749

/gene="HA"

CDS 1..1749

/gene="HA"

/function="receptor binding and fusion protein"

/codon_start=1

/product="hemagglutinin"

/protein_id="WJE87837"

/translation="MKAIIVLLMVVTSNADRICTGITSSNSPHVVKTATQGEVNVTGV

IPLTTTPTKSHFANLKGTETRGKLCPKCLNCTDLDVALGRPKCTGKIPSARVSILHEV

RPVTSGCFPIMHDRTKIRQLPNLLRGYEHVRLSTQNVINTEDAPGGPYEIGTSGSCLN

ITNGKGFFATMAWAVPKNKTATNPLTIEVPYICTEEEDQITVWGFHSDDETQMARLYG

DSKPQKFTSSANGVTTHYVSQIGGFPNQTEDGGLPQSGRIVVDYMVQKSGKTGTITYQ

RGILLPQKVWCASGKSKVIKGSLPLIGEADCLHEKYGGLNKSKPYYTGEHAKAIGNCP

IWVKTPLKLANGTKYRPPAKLLKERGFFGAIAGFLEGGWEGMIAGWHGYTSHGAHGVA

VAADLKSTQEAINKITKNLNSLSELEVKNLQRLSGAMDELHNEILELDEKVDDLRADT

ISSQIELAVLLSNEGIINSEDEHLLALERKLKKMLGPSAVEIGNGCFETKHKCNQTCL

DRIAAGTFDAGEFSLPTFDSLNITAASLNDDGLDNHTILLYYSTAASSLAVTLMIAIF

VVYMVSRDNVSCSICL"

ORIGIN

1 atgaaggcaa taattgtact actcatggta gtaacatcca atgcagatcg aatctgcact

61 gggataacat cgtcaaactc accacatgtc gtcaaaactg ctactcaagg ggaggtcaat

121 gtgaccggtg taataccact gacaacaaca cccaccaaat ctcattttgc aaatctcaaa

181 ggaacagaaa ccagggggaa actatgccca aaatgcctaa actgcacaga tctggatgta

241 gccttgggca gaccaaaatg cacagggaaa ataccctctg caagggtttc aatactccat

301 gaagtcagac ctgttacatc tgggtgcttt cctataatgc atgatagaac aaaaattaga

361 cagctgccta accttctccg aggatacgaa catgtcaggt tatcaactca aaacgttatc

421 aatacagaag atgcaccagg aggaccctac gaaattggaa cctcagggtc ttgcctcaac

481 attaccaatg gaaaaggatt cttcgcaaca atggcttggg ccgtcccaaa aaacaaaaca

541 gcaacaaatc cattaacaat agaagtacca tacatttgta cagaagaaga agaccaaatt

601 accgtttggg ggttccactc tgacgacgag acccaaatgg caaggctcta tggggattca

661 aagccccaga agttcacctc atctgccaac ggagtgacca cacattacgt ctcacagatt

721 ggtggcttcc caaatcaaac agaagacgga ggactaccac aaagtggcag aattgttgtt

781 gattacatgg tgcaaaaatc tggaaaaaca ggaacaatta cctatcaaag aggtatttta

841 ttgcctcaaa aggtgtggtg cgcaagtggc aagagcaagg taataaaagg atccttgccc

901 ttaattggag aagcagattg cctccatgaa aaatacggtg gattaaacaa aagcaagcct

961 tactacacag gggaacatgc aaaggccata ggaaattgcc caatatgggt gaaaacaccc

1021 ttgaagctgg ccaatggaac caaatataga cctcctgcaa aactattaaa ggaaagaggt

1081 ttcttcggag ccattgctgg tttcttagag ggaggatggg aaggaatgat tgcaggttgg

1141 cacggataca catcccatgg ggcacatgga gtagcagtgg cagctgacct taagagcact

1201 caagaggcca taaacaagat aacaaaaaat ctcaactctt tgagtgagct ggaagtaaag

1261 aatcttcaaa gactaagcgg tgccatggat gaactccaca acgaaatact agaactagat

1321 gagaaagtgg atgatctcag agctgataca ataagctcac aaatagaact cgcagtcctg

1381 ctttccaatg aaggaataat aaacagtgaa gatgaacatc tcttggcgct tgaaagaaag

1441 ctgaagaaaa tgctgggccc ctctgctgta gagataggga atggatgctt tgaaaccaaa

1501 cacaagtgca accagacctg tctcgacaga atagctgctg gtacctttga tgcaggagaa

1561 ttttctctcc ccacctttga ttcactgaat attactgctg catctttaaa tgacgatgga

1621 ttggacaatc atactatact gctttactac tcaactgctg cctccagttt ggctgtaaca

1681 ctgatgatag ctatctttgt tgtttatatg gtctccagag acaatgtttc ttgctccatt

1741 tgtctataa

//

LOCUS OR145867 1749 bp cRNA linear VRL 20-JUN-2023

DEFINITION Influenza B virus (B/Beijing/2306/2022) segment 4 hemagglutinin

(HA) gene, complete cds.

ACCESSION OR145867

VERSION OR145867

KEYWORDS .

SOURCE Influenza B virus

ORGANISM Influenza B virus

Viruses; Riboviria; Orthornavirae; Negarnaviricota;

Polyploviricotina; Insthoviricetes; Articulavirales;

Orthomyxoviridae; Betainfluenzavirus; Betainfluenzavirus

influenzae.

REFERENCE 1 (bases 1 to 1749)

AUTHORS Wang,Y.

TITLE Direct Submission

JOURNAL Submitted (20-JUN-2023) Department of Infectious Diseases, Peking

University People's Hospital, Xizhimen South Street, Beijing

100044, China

COMMENT ##Assembly-Data-START##

Sequencing Technology :: Sanger dideoxy sequencing

##Assembly-Data-END##

FEATURES Location/Qualifiers

source 1..1749

/organism="Influenza B virus"

/mol_type="viral cRNA"

/strain="B/Beijing/2306/2022"

/isolate="2306"

/isolation_source="nasal swab"

/host="Homo sapiens"

/db_xref="taxon:11520"

/segment="4"

/country="China: Beijing"

/collection_date="30-Aug-2022"

gene 1..1749

/gene="HA"

CDS 1..1749

/gene="HA"

/function="receptor binding and fusion protein"

/codon_start=1

/product="hemagglutinin"

/protein_id="WJE87838"

/translation="MKAIIVLLMVVTSNADRICTGITSSNSPHVVKTATQGEVNVTGV

IPLTTTPTKSHFANLKGTETRGKLCPKCLNCTDLDVALGRPKCTGKIPSARVSILHEV

RPVTSGCFPIMHDRTKIRQLPNLLRGYEHVRLSTQNVINTEDAPGGPYEIGTSGSCLN

ITNGKGFFATMAWAVPKNKTTTNPLTIEVPYICTEEEDQITVWGFHSDDETQMARLYG

DSKPQKFTSSANGVTTHYVSQIGGFPNQTEDGGLPQSGRIVVDYMVQKSGKTGTITYQ

RGILLPQKVWCASGKSKVIKGSLPLIGEADCLHEKYGGLNKSKPYYTGEHAKAIGNCP

IWVKTPLKLANGTKYRPPAKLLKERGFFGAIAGFLEGGWEGMIAGWHGYTSHGAHGVA

VAADLKSTQEAINKITKNLNSLSELEVKNLQRLASAMDELHNEILELDEKVDDLRADT

ISSQIELAVLLSNEGIINSEDEHLLALERKLKKMLGPSAVEIGNGCFETKHKCNQTCL

DRIAAGTFDAGEFSLPTFDSLNITAASLNDDGLDNHTILLYYSTAASSLAVTLMIAIF

VVYMVSRDNVSCSICL"

ORIGIN

1 atgaaggcaa taattgtact actcatggta gtaacatcca atgcagatcg aatctgcact

61 gggataacat cgtcaaactc accacatgtc gtcaaaactg ctactcaagg ggaggtcaat

121 gtgaccggtg taataccact gacaacaaca cccaccaaat ctcattttgc aaatctcaaa

181 ggaacagaaa ccagggggaa actatgccca aaatgcctaa actgcacaga tctggatgta

241 gccttgggca gaccaaaatg cacagggaaa ataccctctg caagggtttc aatactccat

301 gaagtcagac ctgttacatc tgggtgcttt cctataatgc atgatagaac aaaaattaga

361 cagctgccta accttctccg aggatacgaa catgtcaggt tatcaactca aaacgttatc

421 aatacagaag atgcaccagg aggaccctac gaaattggaa cctcagggtc ttgcctcaac

481 attaccaatg gaaaaggatt cttcgcaaca atggcttggg ccgtcccaaa aaacaaaaca

541 acaacaaatc cattaacaat agaagtacca tacatttgta cagaagaaga agaccaaatt

601 accgtttggg ggttccactc tgacgacgag acccaaatgg caaggctcta tggagattca

661 aagccccaga agttcacctc atctgccaac ggagtgacca cacattacgt ctcacagatt

721 ggtggcttcc caaatcaaac agaagacgga ggactaccac aaagtggcag aattgttgtt

781 gattacatgg tgcaaaaatc tggaaaaaca ggaacaatta cctatcaaag aggtatttta

841 ttgcctcaaa aggtgtggtg cgcaagtggc aagagcaagg taataaaagg atccttgccc

901 ttaattggag aagcagattg ccttcatgaa aaatacggtg gattaaacaa aagcaagcct

961 tactacacag gggaacatgc aaaggccata ggaaattgcc caatatgggt gaaaacaccc

1021 ttgaagctgg ccaatggaac caaatataga cctcctgcaa aactattaaa ggaaagaggt

1081 ttcttcggag ccattgctgg tttcttagag ggaggatggg aaggaatgat tgcaggttgg

1141 cacggataca catcccatgg ggcacatgga gtagcagtgg cagctgacct taagagcact

1201 caagaggcca taaacaagat aacaaaaaat ctcaactctt tgagtgagct ggaagtaaag

1261 aatcttcaaa gactggccag tgccatggat gaactccaca acgaaatact agaactagat

1321 gagaaagtgg atgatctcag agctgataca ataagctcac aaatagaact cgcagtcctg

1381 ctttccaatg aaggaataat aaacagtgaa gatgaacatc tcttggcgct tgaaagaaag

1441 ctgaagaaaa tgctgggccc ctctgctgta gagataggga atggatgctt tgaaaccaaa

1501 cacaagtgca accagacctg tctcgacaga atagctgctg gtacctttga tgcaggagaa

1561 ttttctctcc ccacctttga ttcactgaat attactgctg catctttaaa tgacgatgga

1621 ttggacaatc atactatact gctttactac tcaactgctg cctccagttt ggctgtaaca

1681 ctgatgatag ctatctttgt tgtttatatg gtctccagag acaatgtttc ttgctccatt

1741 tgtctataa

//

LOCUS OR145868 1749 bp cRNA linear VRL 20-JUN-2023

DEFINITION Influenza B virus (B/Beijing/2307/2022) segment 4 hemagglutinin

(HA) gene, complete cds.

ACCESSION OR145868

VERSION OR145868

KEYWORDS .

SOURCE Influenza B virus

ORGANISM Influenza B virus

Viruses; Riboviria; Orthornavirae; Negarnaviricota;

Polyploviricotina; Insthoviricetes; Articulavirales;

Orthomyxoviridae; Betainfluenzavirus; Betainfluenzavirus

influenzae.

REFERENCE 1 (bases 1 to 1749)

AUTHORS Wang,Y.

TITLE Direct Submission

JOURNAL Submitted (20-JUN-2023) Department of Infectious Diseases, Peking

University People's Hospital, Xizhimen South Street, Beijing

100044, China

COMMENT ##Assembly-Data-START##

Sequencing Technology :: Sanger dideoxy sequencing

##Assembly-Data-END##

FEATURES Location/Qualifiers

source 1..1749

/organism="Influenza B virus"

/mol_type="viral cRNA"

/strain="B/Beijing/2307/2022"

/isolate="2307"

/isolation_source="nasal swab"

/host="Homo sapiens"

/db_xref="taxon:11520"

/segment="4"

/country="China: Beijing"

/collection_date="30-Aug-2022"

gene 1..1749

/gene="HA"

CDS 1..1749

/gene="HA"

/function="receptor binding and fusion protein"

/codon_start=1

/product="hemagglutinin"

/protein_id="WJE87839"

/translation="MKAIIVLLMVVTSNADRICTGITSSNSPHVVKTATQGEVNVTGV

IPLTITPTKSHFANLKGTETRGKLCPKCLNCTDLDVALGRPKCTGKIPSARVSILHEV

RPVTSGCFPIMHDRTKIRQLPNLLRGYEHVRLSTQNVINTEDTPGGPYEIGTSGSCLN

ITNGKGFFATMAWAVPKNKTATNPLTIEVPYICTEEEDQITVWGFHSDDETQMARLYG

DSKPQKFTSSANGVTTHYVSQIGGFPNQTEDGGLPQSGRIVVDYMVQKSGKTGTITYQ

RGILLPQKVWCASGKSKVIKGSLPLIGEADCLHEKYGGLNKSKPYYTGEHAKAIGNCP

IWVKTPLKLANGTKYRPPAKLLKERGFFGAIAGFLEGGWEGMIAGWHGYTSHGAHGVA

VAADLKSTQEAINKITKNLNSLSELEVKNLQRLHGAMDELHNEILELDEKVDDLRADT

ISSQIELAVLLSNEGIINSEDEHLLALERKLKKMLGPSAVEIGNGCFETKHKCNQTCL

DRIAAGTFDAGEFSLPTFDSLNITAASLNDDGLDNHTILLYYSTAASSLAVTLMIAIF

VVYMVSRDNVSCSICL"

ORIGIN

1 atgaaggcaa taattgtact actcatggta gtaacatcca atgcagatcg aatctgcact

61 gggataacat cgtcaaactc accacatgtc gtcaaaactg ctactcaagg ggaggtcaat

121 gtgaccggtg taataccact gacaataaca cccaccaaat ctcattttgc aaatctcaaa

181 ggaacagaaa ccagggggaa actatgccca aaatgcctaa actgcacaga tctggatgta

241 gccttgggca gaccaaaatg cacagggaaa ataccctctg caagggtttc aatactccat

301 gaagtcagac ctgttacatc tgggtgcttt cctataatgc atgatagaac aaaaattaga

361 cagctgccta accttctccg aggatacgaa catgtcaggt tatcaactca aaacgttatc

421 aatacagaag atacaccagg aggaccctac gaaattggaa cctcagggtc ttgcctcaac

481 attaccaatg gaaaaggatt cttcgcaaca atggcttggg ccgtcccaaa aaacaaaaca

541 gcaacaaatc cattaacaat agaagtacca tacatttgta cagaagaaga agaccaaatt

601 accgtttggg ggttccactc tgacgacgag acccaaatgg caaggctcta tggggattca

661 aagccccaga agttcacctc atctgccaac ggagtgacca cacattacgt ctcacagatt

721 ggtggcttcc caaatcaaac agaagacgga ggactaccac aaagtggcag aattgttgtt

781 gattacatgg tgcaaaaatc tggaaaaaca ggaacaatta cctatcaaag aggtatttta

841 ttgcctcaaa aggtgtggtg cgcaagtggc aagagcaagg taataaaagg atccttgccc

901 ttaattggag aagcagattg cctccatgaa aaatacggtg gattaaacaa aagcaagcct

961 tactacacag gggaacatgc aaaggccata ggaaattgcc caatatgggt gaaaacaccc

1021 ttgaagctgg ccaatggaac caaatataga cctcctgcaa aactattaaa ggaaagaggt

1081 ttcttcggag ccattgctgg tttcttagag ggaggatggg aaggaatgat tgcaggttgg

1141 cacggataca catcccatgg ggcacatgga gtagcagtgg cagctgacct taagagcact

1201 caagaggcca taaacaagat aacaaaaaat ctcaactctt tgagtgagct ggaagtaaag

1261 aatcttcaaa gactacacgg tgccatggat gaactccaca acgaaatact agaactagat

1321 gagaaagtgg atgatctcag agctgataca ataagctcac aaatagaact cgcagtcctg

1381 ctttccaatg aaggaataat aaacagtgaa gatgaacatc tcttggcgct tgaaagaaag

1441 ctgaagaaaa tgctgggccc ctctgctgta gagataggga atggatgctt tgaaaccaaa

1501 cacaagtgca accagacctg tctcgacaga atagctgctg gtacctttga tgcaggagaa

1561 ttttctctcc ccacctttga ttcactgaat attactgctg catctttaaa tgacgatgga

1621 ttggacaatc atactatact gctttactac tcaactgctg cctccagttt ggctgtaaca

1681 ctgatgatag ctatctttgt tgtttatatg gtctccagag acaatgtttc ttgctccatt

1741 tgtctataa

//

LOCUS OR145869 1749 bp cRNA linear VRL 20-JUN-2023

DEFINITION Influenza B virus (B/Beijing/2308/2022) segment 4 hemagglutinin

(HA) gene, complete cds.

ACCESSION OR145869

VERSION OR145869

KEYWORDS .

SOURCE Influenza B virus

ORGANISM Influenza B virus

Viruses; Riboviria; Orthornavirae; Negarnaviricota;

Polyploviricotina; Insthoviricetes; Articulavirales;

Orthomyxoviridae; Betainfluenzavirus; Betainfluenzavirus

influenzae.

REFERENCE 1 (bases 1 to 1749)

AUTHORS Wang,Y.

TITLE Direct Submission

JOURNAL Submitted (20-JUN-2023) Department of Infectious Diseases, Peking

University People's Hospital, Xizhimen South Street, Beijing

100044, China

COMMENT ##Assembly-Data-START##

Sequencing Technology :: Sanger dideoxy sequencing

##Assembly-Data-END##

FEATURES Location/Qualifiers

source 1..1749

/organism="Influenza B virus"

/mol_type="viral cRNA"

/strain="B/Beijing/2308/2022"

/isolate="2308"

/isolation_source="nasal swab"

/host="Homo sapiens"

/db_xref="taxon:11520"

/segment="4"

/country="China: Beijing"

/collection_date="30-Aug-2022"

gene 1..1749

/gene="HA"

CDS 1..1749

/gene="HA"

/function="receptor binding and fusion protein"

/codon_start=1

/product="hemagglutinin"

/protein_id="WJE87840"

/translation="MKAIIVLLMVVTSNADRICTGITSSNSPHVVKTATQGEVNVTGV

IPLTTTPTKSHFANLKGTETRGKLCPKCLNCTDLDVALGRPKCTGKIPSARVSILHEV

RPVTSGCFPIMHDRTKIRQLPNLLRGYEHVRLSTQNVINTEDAPGGPYEIGTSGSCLN

ITNGKGFFATMAWAVPKNKTATNPLTIEVPYICTEEEDQITVWGFHSDDETQMARLYG

DSKPQKFTSSANGVTTHYVSQIGGFPNQTEDGGLPQSGRIVVDYMVQKSGKTGTITYQ

RGILLPQKVWCASGKSKVIKGSLPLIGEADCLHEKYGGLNKSKPYYTGEHAKAIGNCP

IWVKTPLKLANGTKYRPPAKLLKERGFFGAIAGFLEGGWEGMIAGWHGYTSHGAHGVA

VAADLKSTQEAINKITKNLNSLSELEVKNLQRLSGAMDELHNEILELDEKVDDLRADT

ISSQIELAVLLSNEGIINSEDEHLLALERKLKKMLGPSAVEIGNGCFETKHKCNQTCL

DRIAAGTFDAGEFSLPTFDSLNITAASLNDDGLDNHTILLYYSTAASSLAVTLMIAIF

VVYMVSRDNVSCSICL"

ORIGIN

1 atgaaggcaa taattgtact actcatggta gtaacatcca atgcagatcg aatctgcact

61 gggataacat cgtcaaactc accacatgtc gtcaaaactg ctactcaagg ggaggtcaat

121 gtgaccggtg taataccact gacaacaaca cccaccaaat ctcattttgc aaatctcaaa

181 ggaacagaaa ccagggggaa actatgccca aaatgcctaa actgcacaga tctggatgta

241 gccttgggca gaccaaaatg cacagggaaa ataccctctg caagggtttc aatactccat

301 gaagtcagac ctgttacatc tgggtgcttt cctataatgc atgatagaac aaaaattaga

361 cagctgccta accttctccg aggatacgaa catgtcaggt tatcaactca aaacgttatc

421 aatacagaag atgcaccagg aggaccctac gaaattggaa cctcagggtc ttgcctcaac

481 attaccaatg gaaaaggatt cttcgcaaca atggcttggg ccgtcccaaa aaacaaaaca

541 gcaacaaatc cattaacaat agaagtacca tacatttgta cagaagaaga agaccaaatt

601 accgtttggg ggttccactc tgacgacgag acccaaatgg caagactcta tggggattca

661 aagccccaga agttcacctc atctgccaac ggagtgacca cacattacgt ctcacagatt

721 ggtggcttcc caaatcaaac agaagacgga ggactaccac aaagtggcag aattgttgtt

781 gattacatgg tgcaaaaatc tggaaaaaca ggaacaatta cctatcaaag aggtatttta

841 ttgcctcaaa aggtgtggtg cgcaagtggc aagagcaagg taataaaagg atccttgccc

901 ttaattggag aagcagattg cctccatgaa aaatacggtg gattaaacaa aagcaagcct

961 tactacacag gggaacatgc aaaggccata ggaaattgcc caatatgggt gaaaacaccc

1021 ttgaagctgg ccaatggaac caaatataga cctcctgcaa aactattaaa ggaaagaggt

1081 ttcttcggag ccattgctgg tttcttagag ggaggatggg aaggaatgat tgcaggttgg

1141 cacggataca catcccatgg ggcacatgga gtagcagtgg cagctgacct taagagcact

1201 caagaggcca taaacaagat aacaaaaaat ctcaactctt tgagtgagct ggaagtaaag

1261 aatcttcaaa gactaagcgg tgccatggat gaactccaca acgaaatact agaactagat

1321 gagaaagtgg atgatctcag agctgataca ataagctcac aaatagaact cgcagtcctg

1381 ctttccaatg aaggaataat aaacagtgaa gatgaacatc tcttggcgct tgaaagaaag

1441 ctgaagaaaa tgctgggccc ctctgctgta gagataggga atggatgctt tgaaaccaaa

1501 cacaagtgca accagacctg tctcgacaga atagctgctg gtacctttga tgcaggagaa

1561 ttttctctcc ccacctttga ttcactgaat attactgctg catctttaaa tgacgatgga

1621 ttggacaatc atactatact gctttactac tcaactgctg cctccagttt ggctgtaaca

1681 ctgatgatag ctatctttgt tgtttatatg gtctccagag acaatgtttc ttgctccatt

1741 tgtctataa

//

LOCUS OR145870 1749 bp cRNA linear VRL 20-JUN-2023

DEFINITION Influenza B virus (B/Beijing/2309/2022) segment 4 hemagglutinin

(HA) gene, complete cds.

ACCESSION OR145870

VERSION OR145870

KEYWORDS .

SOURCE Influenza B virus

ORGANISM Influenza B virus

Viruses; Riboviria; Orthornavirae; Negarnaviricota;

Polyploviricotina; Insthoviricetes; Articulavirales;

Orthomyxoviridae; Betainfluenzavirus; Betainfluenzavirus

influenzae.

REFERENCE 1 (bases 1 to 1749)

AUTHORS Wang,Y.

TITLE Direct Submission

JOURNAL Submitted (20-JUN-2023) Department of Infectious Diseases, Peking

University People's Hospital, Xizhimen South Street, Beijing

100044, China

COMMENT ##Assembly-Data-START##

Sequencing Technology :: Sanger dideoxy sequencing

##Assembly-Data-END##

FEATURES Location/Qualifiers

source 1..1749

/organism="Influenza B virus"

/mol_type="viral cRNA"

/strain="B/Beijing/2309/2022"

/isolate="2309"

/isolation_source="nasal swab"

/host="Homo sapiens"

/db_xref="taxon:11520"

/segment="4"

/country="China: Beijing"

/collection_date="30-Aug-2022"

gene 1..1749

/gene="HA"

CDS 1..1749

/gene="HA"

/function="receptor binding and fusion protein"

/codon_start=1

/product="hemagglutinin"

/protein_id="WJE87841"

/translation="MKAIIVLLMVVTSNADRICTGITSSNSPHVVKTATQGEVNVTGV

IPLTTTPTKSHFANLKGTETRGKLCPKCLNCTDLDVALGRPKCTGKIPSARVSILHEV

RPVTSGCFPIMHDRTKIRQLPNLLRGYEHVRLSTQNVINTEDAPGGPYEIGTSGSCLN

ITNGKGFFVTMAWAVPKNKTATNPLTIEVPYICTEEEDQITVWGFHSDDETQMARLYG

DSKPQKFTSSANGVTTHYVSQIGGFPNQTEDGGLPQSGRIVVDYMVQKSGKTGTITYQ

RGILLPQKVWCASGKSKVIKGSLPLIGEADCLHEKYGGLNKSKPYYTGEHAKAIGNCP

IWVKTPLKLANGTKYRPPAKLLKERGFFGAIAGFLEGGWEGMIAGWHGYTSHGAHGVA

VAADLKSTQEAINKITKNLNSLSELEVKNLQRLSGAMDELHNEILELDEKVDDLRADT

ISSQIELAVLLSNEGIINSEDEHLLALERKLKKMLGPSAVEIGNGCFETKHKCNQTCL

DRIAAGTFDAGEFSLPTFDSLNITAASLNDDGLDNHTILLYYSTAASSLAVTLMIAIF

VVYMVSRDNVSCSICL"

ORIGIN

1 atgaaggcaa taattgtact actcatggta gtaacatcca atgcagatcg aatctgcact

61 gggataacat cgtcaaactc accacatgtc gtcaaaactg ctactcaagg ggaggtcaat

121 gtgaccggtg taataccact gacaacaaca cccaccaaat ctcattttgc aaatctcaaa

181 ggaacagaaa ccagggggaa actatgccca aaatgcctaa actgcacaga tctggatgta

241 gccttgggca gaccaaaatg cacagggaaa ataccctctg caagggtttc aatactccat

301 gaagtcagac ctgttacatc tgggtgcttt cctataatgc atgatagaac aaaaattaga

361 cagctgccta accttctccg aggatacgaa catgtcaggt tatcaactca aaacgttatc

421 aatacagaag atgcaccagg aggaccctac gaaattggaa cctcagggtc ttgcctaaac

481 attaccaatg gaaaaggatt cttcgtaaca atggcttggg ccgtcccaaa aaacaaaaca

541 gcaacaaatc cattaacaat agaagtacca tacatttgta cagaagaaga agaccaaatt

601 accgtttggg ggttccactc tgacgacgag acccaaatgg caaggctcta tggggattca

661 aagccccaga agttcacctc atctgccaac ggagtgacca cacattacgt ctcacagatt

721 ggtggcttcc caaatcaaac agaagacgga ggactaccac aaagtggcag aattgttgtt

781 gattacatgg tgcaaaaatc tggaaaaaca ggaacaatta cctatcaaag aggtatttta

841 ttgcctcaaa aggtgtggtg cgcaagtggc aagagcaagg taataaaagg atccttgccc

901 ttaattggag aagcagattg cctccatgaa aaatacggtg gattaaacaa aagcaagcct

961 tactacacag gggaacatgc aaaggccata ggaaattgcc caatatgggt gaaaacaccc

1021 ttgaagctgg ccaatggaac caaatataga cctcctgcaa aattattaaa ggaaagaggt

1081 ttcttcggag ccattgctgg tttcttagag ggaggatggg aaggaatgat tgcaggttgg

1141 cacggataca catcccatgg ggcacatgga gtagcagtgg cagctgacct taagagcact

1201 caagaggcta taaacaagat aacaaaaaat ctcaactctt tgagtgagct ggaagtaaag

1261 aatcttcaaa gactaagcgg tgccatggat gaacttcaca acgaaatact agaactagat

1321 gagaaagtgg atgatctcag agctgataca ataagctcac aaatagaact cgcagtcctg

1381 ctttccaatg aaggaataat aaacagtgaa gatgaacatc tcttggcgct tgaaagaaag

1441 ctgaagaaaa tgctgggccc ctctgctgta gagataggga atggatgctt tgaaaccaaa

1501 cacaagtgca accagacctg tctcgacaga atagctgctg gtacctttga tgcaggagaa

1561 ttttctctcc ccacctttga ttcactgaat attactgctg catctttaaa tgacgatgga

1621 ttggacaatc atactatact gctttactac tcaactgctg cctccagttt ggctgtaaca

1681 ctgatgatag ctatctttgt tgtttatatg gtctccagag acaatgtttc ttgctccatt

1741 tgtctataa

//

LOCUS OR145871 1749 bp cRNA linear VRL 20-JUN-2023

DEFINITION Influenza B virus (B/Beijing/2310/2022) segment 4 hemagglutinin

(HA) gene, complete cds.

ACCESSION OR145871

VERSION OR145871

KEYWORDS .

SOURCE Influenza B virus

ORGANISM Influenza B virus

Viruses; Riboviria; Orthornavirae; Negarnaviricota;

Polyploviricotina; Insthoviricetes; Articulavirales;

Orthomyxoviridae; Betainfluenzavirus; Betainfluenzavirus

influenzae.

REFERENCE 1 (bases 1 to 1749)

AUTHORS Wang,Y.

TITLE Direct Submission

JOURNAL Submitted (20-JUN-2023) Department of Infectious Diseases, Peking

University People's Hospital, Xizhimen South Street, Beijing

100044, China

COMMENT ##Assembly-Data-START##

Sequencing Technology :: Sanger dideoxy sequencing

##Assembly-Data-END##

FEATURES Location/Qualifiers

source 1..1749

/organism="Influenza B virus"

/mol_type="viral cRNA"

/strain="B/Beijing/2310/2022"

/isolate="2310"

/isolation_source="nasal swab"

/host="Homo sapiens"

/db_xref="taxon:11520"

/segment="4"

/country="China: Beijing"

/collection_date="30-Aug-2022"

gene 1..1749

/gene="HA"

CDS 1..1749

/gene="HA"

/function="receptor binding and fusion protein"

/codon_start=1

/product="hemagglutinin"

/protein_id="WJE87842"

/translation="MKAIIVLLMVVTSNADRICTGITSSNSPHVVKTATQGEVNVTGV

IPLTTTPTKSHFANLKGTETRGKLCPKCLNCTDLDVALGRPKCTGKIPSARVSILHEV

RPVTSGCFPIMHDRTKIRQLPNLLRGYEHVRLSTQNVINTEDAPGGPYEIGTSGSCLN

ITNGKGFFVTMAWAVPKNKTATNPLTIEVPYICTEEEDQITVWGFHSDDETQMARLYG

DSKPQKFTSSANGVTTHYVSQIGGFPNQTEDGGLPQSGRIVVDYMVQKSGKTGTITYQ

RGILLPQKVWCASGKSKVIKGSLPLIGEADCLHEKYGGLNKSKPYYTGEHAKAIGNCP

IWVKTPLKLANGTKYRPPAKLLKERGFFGAIAGFLEGGWEGMIAGWHGYTSHGAHGVA

VAADLKSTQEAINKITKNLNSLSELEVKNLQRLSGAMDELHNEILELDEKVDDLRADT

ISSQIELAVLLSNEGIINSEDEHLLALERKLKKMLGPSAVEIGNGCFETKHKCNQTCL

DRIAAGTFDAGEFSLPTFDSLNITAASLNDDGLDNHTILLYYSTAASSLAVTLMIAIF

VVYMVSRDNVSCSICL"

ORIGIN

1 atgaaggcaa taattgtact actcatggta gtaacatcca atgcagatcg aatctgcact

61 gggataacat cgtcaaactc accacatgtc gtcaaaactg ctactcaagg ggaggtcaat

121 gtgaccggtg taataccact gacaacaaca cccaccaaat ctcattttgc aaatctcaaa

181 ggaacagaaa ccagggggaa actatgccca aaatgcctaa actgcacaga tctggatgta

241 gccttgggca gaccaaaatg cacagggaaa ataccctctg caagggtttc aatactccat

301 gaagtcagac ctgttacatc tgggtgcttt cctataatgc atgatagaac aaaaattaga

361 cagctgccta accttctccg aggatacgaa catgtcaggt tatcaactca aaacgttatc

421 aatacagaag atgcaccagg aggaccctac gaaattggaa cctcagggtc ttgcctcaac

481 attaccaatg gaaaaggatt cttcgtaaca atggcttggg ccgtcccaaa aaacaaaaca

541 gcaacaaatc cattaacaat agaagtacca tacatttgta cagaagaaga agaccaaatt

601 accgtttggg ggttccactc tgacgacgag acccaaatgg caaggctcta tggggattca

661 aagccccaga agttcacctc atctgccaac ggagtgacca cacattacgt ctcacagatt

721 ggtggcttcc caaatcaaac agaagacgga ggactaccac aaagtggcag aattgttgtt

781 gattacatgg tgcaaaaatc tggaaaaaca ggaacaatta cctatcaaag aggtatttta

841 ttgcctcaaa aggtgtggtg cgcaagtggc aagagcaagg taataaaagg atccttgccc

901 ttaattggag aagcagattg cctccatgaa aaatacggtg gattaaacaa aagcaagcct

961 tactacacag gggaacatgc aaaggccata ggaaattgcc caatatgggt gaaaacaccc

1021 ttgaagctgg ccaatggaac caaatataga cctcctgcaa aattattaaa ggaaagaggt

1081 ttcttcggag ccattgctgg tttcttagag ggaggatggg aaggaatgat tgcaggttgg

1141 cacggataca catcccatgg ggcacatgga gtagcagtgg cagctgacct taagagcact

1201 caagaggcta taaacaagat aacaaaaaat ctcaactctt tgagtgagct ggaagtaaag

1261 aatcttcaaa gactaagcgg tgccatggat gaacttcaca acgaaatact agaactagat

1321 gagaaagtgg atgatctcag agctgataca ataagctcac aaatagaact cgcagtcctg

1381 ctttccaatg aaggaataat aaacagtgaa gatgaacatc tcttggcgct tgaaagaaag

1441 ctgaagaaaa tgctgggccc ctctgctgta gagataggga atggatgctt tgaaaccaaa

1501 cacaagtgca accagacctg tctcgacaga atagctgctg gtacctttga tgcaggagaa

1561 ttttctctcc ccacctttga ttcactgaat attactgctg catctttaaa tgacgatgga

1621 ttggacaatc atactatact gctttactac tcaactgctg cctccagttt ggctgtaaca

1681 ctgatgatag ctatctttgt tgtttatatg gtctccagag acaatgtttc ttgctccatt

1741 tgtctataa

//

LOCUS OR145872 1749 bp cRNA linear VRL 20-JUN-2023

DEFINITION Influenza B virus (B/Beijing/2311/2022) segment 4 hemagglutinin

(HA) gene, complete cds.

ACCESSION OR145872

VERSION OR145872

KEYWORDS .

SOURCE Influenza B virus

ORGANISM Influenza B virus

Viruses; Riboviria; Orthornavirae; Negarnaviricota;

Polyploviricotina; Insthoviricetes; Articulavirales;

Orthomyxoviridae; Betainfluenzavirus; Betainfluenzavirus

influenzae.

REFERENCE 1 (bases 1 to 1749)

AUTHORS Wang,Y.

TITLE Direct Submission

JOURNAL Submitted (20-JUN-2023) Department of Infectious Diseases, Peking

University People's Hospital, Xizhimen South Street, Beijing

100044, China

COMMENT ##Assembly-Data-START##

Sequencing Technology :: Sanger dideoxy sequencing

##Assembly-Data-END##

FEATURES Location/Qualifiers

source 1..1749

/organism="Influenza B virus"

/mol_type="viral cRNA"

/strain="B/Beijing/2311/2022"

/isolate="2311"

/isolation_source="nasal swab"

/host="Homo sapiens"

/db_xref="taxon:11520"

/segment="4"

/country="China: Beijing"

/collection_date="30-Aug-2022"

gene 1..1749

/gene="HA"

CDS 1..1749

/gene="HA"

/function="receptor binding and fusion protein"

/codon_start=1

/product="hemagglutinin"

/protein_id="WJE87843"

/translation="MKAIIVLLMVVTSNADRICTGITSSNSPHVVKTATQGEVNVTGV

ISLTTTPTKSHFANLKGTETRGKLCPKCLNCTDLDVALGRPKCTGKIPSARVSILHEV

RPVTSGCFPIMHDRTKIRQLPNLLRGYEHVRLSTHNVINAEDAPGGPYEIGTSGSCPN

ITNGKGFFATMAWAVPKNQTATNPLTIEVPYICTEEEDQITVWGFHSDDETQMAKLYG

DSKPQKFTSSANGMTTHYVSQIGGFPNQTEDGGLQQSGRIVVDYMVQKSGKTGTITYQ

RGILLPQKVWCASGKSKVIKGSLPLIGEADCLHEKYGGLNKSKPYYTGEHAKAIGNCP

IWVKTPLKLANGTKYRPPAKLLKERGFFGAIAGFLEGGWEGMIAGWHGYTSHGAHGVA

VAADLKSTQEAINKITKNLNSLSELEVKNLQRLSGAMDELHNEILELDEKVDDLRADT

ISSQIELAVLLSNEGIINSEDEHLLALERKLKKMLGPSAVEIGNGCFETKHKCNQTCL

DRIAAGTFDAGEFSLPTFDSLNITAASLNDDGLDNHTILLYYSTAASSLAVTLMIAIF

VVYMVSRDNVSCSICL"

ORIGIN

1 atgaaggcaa taattgtact actcatggta gtaacatcca atgcagatcg aatctgcact

61 gggataacat cgtcaaactc accacatgtc gtcaaaactg ctactcaagg ggaggtcaat

121 gtgaccggtg taatatcact gacaacaaca cccaccaaat ctcattttgc aaatctcaaa

181 ggaacagaaa ccagggggaa actatgccca aaatgcctca actgcacaga tctggatgta

241 gccttgggca gaccaaaatg cacagggaaa ataccctctg caagggtttc aatactccat

301 gaagtcagac ctgttacatc tgggtgcttt cctataatgc atgatagaac aaaaattaga

361 cagctgccta accttctccg aggatacgaa catgtcagat tatcaaccca caacgttatc

421 aatgcagaag atgcaccagg aggaccctac gaaattggaa cctcagggtc ttgccctaac

481 attaccaatg gaaaaggatt cttcgcaaca atggcttggg ccgtcccaaa aaaccaaaca

541 gcaacaaatc cattaacaat agaagtacca tacatttgta cagaagaaga agaccaaatt

601 accgtttggg ggttccactc tgacgacgag acccaaatgg caaagctcta tggggactca

661 aagccccaga agttcacctc atctgccaac ggaatgacca cacattacgt ttcacagatt

721 ggtggcttcc caaatcaaac agaagacgga ggactacaac aaagtggcag aattgttgtt

781 gattacatgg tgcaaaaatc tggaaaaaca ggaacaatta cctatcaaag aggtatttta

841 ttgcctcaaa aggtgtggtg cgcaagtggc aagagcaagg taataaaagg atccttgccc

901 ttaattggag aagcagattg cctccatgaa aaatacggtg gattaaacaa aagcaagcct

961 tactacacag gggaacatgc aaaggccata ggaaattgcc caatatgggt gaaaacaccc

1021 ttgaagctgg ccaatggaac caaatataga cctcctgcaa aactattaaa ggaaagaggt

1081 ttcttcggag ccattgctgg tttcttagag ggaggatggg aaggaatgat tgcaggttgg

1141 cacggataca catcccatgg ggcacatgga gtagcggtgg cagctgacct taagagcact

1201 caagaggcca taaacaagat aacaaaaaat ctcaactctt tgagtgagct ggaagtaaag

1261 aatcttcaaa gactaagcgg tgccatggat gaactccaca acgaaatact agaactagat

1321 gagaaagtgg atgatctcag agctgataca ataagctcac aaatagaact cgcagtcctg

1381 ctttccaatg aaggaataat aaacagtgaa gatgaacatc tcttggcgct tgaaagaaag

1441 ctgaagaaaa tgctgggccc ctctgctgta gagataggga atggatgctt tgaaaccaaa

1501 cacaagtgca accagacctg tctcgacaga atagctgctg gtacctttga tgcaggagaa

1561 ttttctctcc ccacctttga ttcactgaat attactgctg catctttaaa tgacgatgga

1621 ttggacaatc atactatact gctttactac tcaactgctg cctccagttt ggctgtaaca

1681 ctgatgatag ctatctttgt tgtttatatg gtctccagag acaatgtttc ttgctccatt

1741 tgtctataa

//

LOCUS OR145873 1749 bp cRNA linear VRL 20-JUN-2023

DEFINITION Influenza B virus (B/Beijing/2312/2022) segment 4 hemagglutinin

(HA) gene, complete cds.

ACCESSION OR145873

VERSION OR145873

KEYWORDS .

SOURCE Influenza B virus

ORGANISM Influenza B virus

Viruses; Riboviria; Orthornavirae; Negarnaviricota;

Polyploviricotina; Insthoviricetes; Articulavirales;

Orthomyxoviridae; Betainfluenzavirus; Betainfluenzavirus

influenzae.

REFERENCE 1 (bases 1 to 1749)

AUTHORS Wang,Y.

TITLE Direct Submission

JOURNAL Submitted (20-JUN-2023) Department of Infectious Diseases, Peking

University People's Hospital, Xizhimen South Street, Beijing

100044, China

COMMENT ##Assembly-Data-START##

Sequencing Technology :: Sanger dideoxy sequencing

##Assembly-Data-END##

FEATURES Location/Qualifiers

source 1..1749

/organism="Influenza B virus"

/mol_type="viral cRNA"

/strain="B/Beijing/2312/2022"

/isolate="2312"

/isolation_source="nasal swab"

/host="Homo sapiens"

/db_xref="taxon:11520"

/segment="4"

/country="China: Beijing"

/collection_date="30-Aug-2022"

gene 1..1749

/gene="HA"

CDS 1..1749

/gene="HA"

/function="receptor binding and fusion protein"

/codon_start=1

/product="hemagglutinin"

/protein_id="WJE87844"

/translation="MKAIIVLLMVVTSNADRICTGITSSNSPHVVKTATQGEVNVTGV

IPLTTTPTKSHFANLKGTETRGKLCPKCLNCTDLDVALGRPKCTGKIPSARVSILHEV

RPVTSGCFPIMHDRTKIRQLPNLLRGYEHVRLSTQNVINTEDAPGGPYEIGTSGSCLN

ITNGKGFFATMAWAVPKNKTATNPLTIEVPYICTEEEDQITVWGFHSDDETQMARLYG

DSKPQKFTSSANGVTTHYVSQIGGFPNQTEDGGLPQSGRIVVDYMVQKSGKTGTITYQ

RGILLPQKVWCASGKSKVIKGSLPLIGEADCLHEKYGGLNKSKPYYTGEHAKAIGNCP

IWVKTPLKLANGTKYRPPAKLLKERGFFGAIAGFLEGGWEGMIAGWHGYTSHGAHGVA

VAADLKSTQEAINKITKNLNSLSELEVKNLQRLGSAMDELHNEILELDEKVDDLRADT

ISSQIELAVLLSNEGIINSEDEHLLALERKLKKMLGPSAVEIGNGCFETKHKCNQTCL

DRIAAGTFDAGEFSLPTFDSLNITAASLNDDGLDNHTILLYYSTAASSLAVTLMIAIF

VVYMVSRDNVSCSICL"

ORIGIN

1 atgaaggcaa taattgtact actcatggta gtaacatcca atgcagatcg aatctgcact

61 gggataacat cgtcaaactc accacatgtc gtcaaaactg ctactcaagg ggaggtcaat

121 gtgaccggtg taataccact gacaacaaca cccaccaaat ctcattttgc aaatctcaaa

181 ggaacagaaa ccagggggaa actatgccca aaatgcctaa actgcacaga tctggatgta

241 gccttgggca gaccaaaatg cacagggaaa ataccctctg caagggtttc aatactccat

301 gaagtcagac ctgttacatc tgggtgcttt cctataatgc atgatagaac aaaaattaga

361 cagctgccta accttctccg aggatacgaa catgtcaggt tatcaactca aaacgttatc

421 aatacagaag atgcaccagg aggaccctac gaaattggaa cctcagggtc ttgcctcaac

481 attaccaatg gaaaaggatt cttcgcaaca atggcttggg ccgtcccaaa aaacaaaaca

541 gcaacaaatc cattaacaat agaagtacca tacatttgta cagaagaaga agaccaaatt

601 accgtttggg ggttccactc tgacgacgag acccaaatgg caaggctcta tggggattca

661 aagccccaga agttcacctc atctgccaac ggagtgacca cacattacgt ctcacagatt

721 ggtggcttcc caaatcaaac agaagacgga ggactaccac aaagtggcag aattgttgtt

781 gattacatgg tgcaaaaatc tggaaaaaca ggaacaatta cctatcaaag aggtatttta

841 ttgcctcaaa aggtgtggtg cgcaagtggc aagagcaagg taataaaagg atccttgccc

901 ttaattggag aagcagattg cctccatgaa aaatacggtg gattaaacaa aagcaagcct

961 tactacacag gggaacatgc aaaggccata ggaaattgcc caatatgggt gaaaacaccc

1021 ttgaagctgg ccaatggaac caaatataga cctcctgcaa aactattaaa ggaaagaggt

1081 ttcttcggag ccattgctgg tttcttagag ggaggatggg aaggaatgat tgcaggttgg

1141 cacggataca catcccatgg ggcacatgga gtagcagtgg cagctgacct taagagcact

1201 caagaggcca taaacaagat aacaaaaaat ctcaactctt tgagtgagct agaagtaaag

1261 aatcttcaaa gactaggcag tgccatggat gaactccaca acgaaatact agaactagat

1321 gagaaagtgg atgatctcag agctgataca ataagctcac aaatagaact cgcagtcctg

1381 ctttccaatg aaggaataat aaacagtgaa gatgaacatc tcttggcgct tgaaagaaag

1441 ctgaagaaaa tgctgggccc ctctgctgta gagataggga atggatgctt tgaaaccaaa

1501 cacaagtgca accagacctg tctcgacaga atagctgctg gtacctttga tgcaggagaa

1561 ttttctctcc ccacctttga ttcactgaat attactgctg catctttaaa tgacgatgga

1621 ttggacaatc atactatact gctttactac tcaactgctg cctccagttt ggctgtaaca

1681 ctgatgatag ctatctttgt tgtttatatg gtctccagag acaatgtttc ttgctccatt

1741 tgtctataa

//

LOCUS OR145874 1749 bp cRNA linear VRL 20-JUN-2023

DEFINITION Influenza B virus (B/Beijing/2313/2022) segment 4 hemagglutinin

(HA) gene, complete cds.

ACCESSION OR145874

VERSION OR145874

KEYWORDS .

SOURCE Influenza B virus

ORGANISM Influenza B virus

Viruses; Riboviria; Orthornavirae; Negarnaviricota;

Polyploviricotina; Insthoviricetes; Articulavirales;

Orthomyxoviridae; Betainfluenzavirus; Betainfluenzavirus

influenzae.

REFERENCE 1 (bases 1 to 1749)

AUTHORS Wang,Y.

TITLE Direct Submission

JOURNAL Submitted (20-JUN-2023) Department of Infectious Diseases, Peking

University People's Hospital, Xizhimen South Street, Beijing

100044, China

COMMENT ##Assembly-Data-START##

Sequencing Technology :: Sanger dideoxy sequencing

##Assembly-Data-END##

FEATURES Location/Qualifiers

source 1..1749

/organism="Influenza B virus"

/mol_type="viral cRNA"

/strain="B/Beijing/2313/2022"

/isolate="2313"

/isolation_source="nasal swab"

/host="Homo sapiens"

/db_xref="taxon:11520"

/segment="4"

/country="China: Beijing"

/collection_date="30-Aug-2022"

gene 1..1749

/gene="HA"

CDS 1..1749

/gene="HA"

/function="receptor binding and fusion protein"

/codon_start=1

/product="hemagglutinin"

/protein_id="WJE87845"

/translation="MKAIIVLLMVVTSNADRICTGITSSNSPHVVKTATQGEVNVTGV

IPLTTTPTKSHFANLKGTETRGKLCPKCLNCTDLDVALGRPKCTGKIPSARVSILHEV

RPVTSGCFPIMHDRTKIRQLPNLLRGYEHVRLSTQNVINTEDAPGGPYEIGTSGSCLN

ITNGKGFFVTMAWAVPKNKTATNPLTIEVPYICTEEEDQITVWGFHSDDETQMARLYG

DSKPQKFTSSANGVTTHYVSQIGGFPNQTEDGGLPQSGRIVVDYMVQKSGKTGTITYQ

RGILLPQKVWCASGKSKVIKGSLPLIGEADCLHEKYGGLNKSKPYYTGEHAKAIGNCP

IWVKTPLKLANGTKYRPPAKLLKERGFFGAIAGFLEGGWEGMIAGWHGYTSHGAHGVA

VAADLKSTQEAINKITKNLNSLSELEVKNLQRLSGAMDELHNEILELDEKVDDLRADT

ISSQIELAVLLSNEGIINSEDEHLLALERKLKKMLGPSAVEIGNGCFETKHKCNQTCL

DRIAAGTFDAGEFSLPTFDSLNITAASLNDDGLDNHTILLYYSTAASSLAVTLMIAIF

VVYMVSRDNVSCSICL"

ORIGIN

1 atgaaggcaa taattgtact actcatggta gtaacatcca atgcagatcg aatctgcact

61 gggataacat cgtcaaactc accacatgtc gtcaaaactg ctactcaagg ggaggtcaat

121 gtgaccggtg taataccact gacaacaaca cccaccaaat ctcattttgc aaatctcaaa

181 ggaacagaaa ccagggggaa actatgccca aaatgcctaa actgcacaga tctggatgta

241 gccttgggca gaccaaaatg cacagggaaa ataccctctg caagggtttc aatactccat

301 gaagtcagac ctgttacatc tgggtgcttt cctataatgc atgatagaac aaaaattaga

361 cagctgccta accttctccg aggatacgaa catgtcaggt tatcaactca aaacgttatc

421 aatacagaag atgcaccagg aggaccctac gaaattggaa cctcagggtc ttgcctcaac

481 attaccaatg gaaaaggatt cttcgtaaca atggcttggg ccgtcccaaa aaacaaaaca

541 gcaacaaatc cattaacaat agaagtacca tacatttgta cagaagaaga agaccaaatt

601 accgtttggg ggttccactc tgacgacgag acccaaatgg caaggctcta tggggattca

661 aagccccaga agttcacctc atctgccaat ggagtgacca cacattacgt ctcacagatt

721 ggtggcttcc caaatcaaac agaagacgga ggactaccac aaagtggcag aattgttgtt

781 gattacatgg tgcaaaaatc tggaaaaaca ggaacaatta cctatcaaag aggtatttta

841 ttgcctcaaa aggtgtggtg cgcaagtggc aagagcaagg taataaaagg atccttgccc

901 ttaattggag aagcagattg cctccatgaa aaatacggtg gattaaacaa aagcaagcct

961 tactacacag gggaacatgc aaaggccata ggaaattgcc caatatgggt gaaaacaccc

1021 ttgaagctgg ccaatggaac caaatataga cctcctgcaa aattattaaa ggaaagaggt

1081 ttcttcggag ccattgctgg tttcttagag ggaggatggg aaggaatgat tgcaggttgg

1141 cacggataca catcccatgg ggcacatgga gtagcagtgg cagctgacct taagagcact

1201 caagaggcta taaacaagat aacaaaaaat ctcaactctt tgagtgagct ggaagtaaag

1261 aatcttcaaa gactaagcgg tgccatggat gaacttcaca acgaaatact agaactagat

1321 gagaaagtgg atgatctcag agctgataca ataagctcac aaatagaact cgcagtcctg

1381 ctttccaatg aaggaataat aaacagtgaa gatgaacatc tcttggcgct tgaaagaaag

1441 ctgaagaaaa tgctgggccc ctctgctgta gagataggga atggatgctt tgaaaccaaa

1501 cacaagtgca accagacctg tctcgacaga atagctgctg gtacctttga tgcaggagaa

1561 ttttctctcc ccacctttga ttcactgaat attactgctg catctttaaa tgacgatgga

1621 ttggacaatc atactatact gctttactac tcaactgctg cctccagttt ggctgtaaca

1681 ctgatgatag ctatctttgt tgtttatatg gtctccagag acaatgtttc ttgctccatt

1741 tgtctataa

//

LOCUS OR145875 1749 bp cRNA linear VRL 20-JUN-2023

DEFINITION Influenza B virus (B/Beijing/2314/2022) segment 4 hemagglutinin

(HA) gene, complete cds.

ACCESSION OR145875

VERSION OR145875

KEYWORDS .

SOURCE Influenza B virus

ORGANISM Influenza B virus

Viruses; Riboviria; Orthornavirae; Negarnaviricota;

Polyploviricotina; Insthoviricetes; Articulavirales;

Orthomyxoviridae; Betainfluenzavirus; Betainfluenzavirus

influenzae.

REFERENCE 1 (bases 1 to 1749)

AUTHORS Wang,Y.

TITLE Direct Submission

JOURNAL Submitted (20-JUN-2023) Department of Infectious Diseases, Peking

University People's Hospital, Xizhimen South Street, Beijing

100044, China

COMMENT ##Assembly-Data-START##

Sequencing Technology :: Sanger dideoxy sequencing

##Assembly-Data-END##

FEATURES Location/Qualifiers

source 1..1749

/organism="Influenza B virus"

/mol_type="viral cRNA"

/strain="B/Beijing/2314/2022"

/isolate="2314"

/isolation_source="nasal swab"

/host="Homo sapiens"

/db_xref="taxon:11520"

/segment="4"

/country="China: Beijing"

/collection_date="30-Aug-2022"

gene 1..1749

/gene="HA"

CDS 1..1749

/gene="HA"

/function="receptor binding and fusion protein"

/codon_start=1

/product="hemagglutinin"

/protein_id="WJE87846"

/translation="MKAIIVLLMVVTSNADRICTGITSSNSPHVVKTATQGEVNVTGV

IPLTTTPTKSHFANLKGTETRGKLCPKCLNCTDLDVALGRPKCTGKIPSAKVSILHEV

RPVTSGCFPIMHDRTKIRQLPNLLRGYEHVRLSTNNVINAEDAPGGPYEIGTSGSCPN

ITNGKGFFATMAWAVPKNKTATNPLTIEVPYICTEEEDQITVWGFHSDDETQMAKLYG

DSKPQKFTSSANGMTTHYVSQIGGFPNQTEDGGLQQSGRIVVDYMVQKSGKTGTITYQ

RGILLPQKVWCASGKSKVIKGSLPLIGEADCLHEKYGGLNKSKPYYTGEHAKAIGNCP

IWVKTPLKLANGTKYRPPAKLLKERGFFGAIAGFLEGGWEGMIAGWHGYTSHGAHGVA

VAADLKSTQEAINKITKNLNSLSELEVKNLQRLSGAMDELHNEILELDEKVDDLRADT

ISSQIELAVLLSNEGIINSEDEHLLALERKLKKMLGPSAVEIGNGCFETKHKCNQTCL

DRIAAGTFDAGEFSLPTFDSLNITAASLNDDGLDNHTILLYYSTAASSLAVTLMIAIF

VVYMVSRDNVSCSICL"

ORIGIN

1 atgaaggcaa taattgtact actcatggta gtaacatcca atgcagatcg aatctgcact

61 gggataacat cgtcaaactc accacatgtc gtcaaaactg ctactcaagg ggaggtcaat

121 gtgaccggtg taataccact gacaacaaca cccaccaaat ctcattttgc aaatctcaaa

181 ggaacagaaa ccagggggaa actatgccca aaatgcctca actgcacaga tctggatgta

241 gccttgggca gaccaaaatg cacagggaaa ataccctctg caaaggtttc aatactccat

301 gaagtcagac ctgttacatc tgggtgcttt cctataatgc atgatagaac aaaaattaga

361 cagctgccta accttctccg aggatacgaa catgtcagat tatcaactaa caacgttatc

421 aatgcagaag atgcaccagg aggaccctac gaaattggaa cctcagggtc ttgccctaac

481 attaccaatg gaaaaggatt cttcgcaaca atggcttggg ccgtcccaaa aaacaaaaca

541 gcaacaaatc cattaacaat agaagtacca tacatttgta cagaagaaga agaccaaatt

601 accgtttggg ggttccactc tgacgacgag acccaaatgg caaagctcta tggggactca

661 aagccccaga agttcacctc atctgccaac ggaatgacca cacattacgt ttcacagatt

721 ggtggcttcc caaatcaaac agaagacgga ggactacaac aaagtggcag aattgttgtt

781 gattacatgg tgcaaaaatc tggaaaaaca ggaacaatta cctatcaaag aggtatttta

841 ttgcctcaaa aggtgtggtg cgcaagtggc aagagcaagg taataaaagg atccttgccc

901 ttaattggag aagcagattg cctccatgaa aaatacggtg gattaaacaa aagcaagcct

961 tactacacag gggaacatgc aaaggccata ggaaattgcc caatatgggt gaaaacaccc

1021 ttgaagctgg ccaatggaac caaatataga cctcctgcaa aactattaaa ggaaagaggt

1081 ttcttcggag ccattgctgg tttcttagag ggaggatggg aaggaatgat tgcaggttgg

1141 cacggataca catcccatgg ggcacatgga gtagcggtgg cagctgacct taagagcact

1201 caagaggcca taaacaagat aacaaaaaat ctcaactctt tgagtgagct ggaagtaaag

1261 aatcttcaaa gactaagcgg tgccatggat gaactccata acgagatact agaactagat

1321 gaaaaagtgg atgatctcag agctgataca ataagctcac aaatagaact cgcagtcctg

1381 ctttccaatg aaggaataat aaacagtgaa gatgaacatc tcttggcgct tgaaagaaag

1441 ctgaagaaaa tgctgggccc ctctgctgta gagataggga atggatgctt tgaaaccaaa

1501 cacaagtgca accagacctg tctcgacaga atagctgctg gtacctttga tgcaggagaa

1561 ttttctctcc ccacctttga ttcactgaat attactgctg catctttaaa tgacgatgga

1621 ttggacaatc atactatact gctttactac tcaactgctg cctccagttt ggctgtaaca

1681 ctgatgatag ctatctttgt tgtttatatg gtctccagag acaatgtttc ttgctccatt

1741 tgtctataa

//

LOCUS OR145876 1749 bp cRNA linear VRL 20-JUN-2023

DEFINITION Influenza B virus (B/Beijing/2315/2022) segment 4 hemagglutinin

(HA) gene, complete cds.

ACCESSION OR145876

VERSION OR145876

KEYWORDS .

SOURCE Influenza B virus

ORGANISM Influenza B virus

Viruses; Riboviria; Orthornavirae; Negarnaviricota;

Polyploviricotina; Insthoviricetes; Articulavirales;

Orthomyxoviridae; Betainfluenzavirus; Betainfluenzavirus

influenzae.

REFERENCE 1 (bases 1 to 1749)

AUTHORS Wang,Y.

TITLE Direct Submission

JOURNAL Submitted (20-JUN-2023) Department of Infectious Diseases, Peking

University People's Hospital, Xizhimen South Street, Beijing

100044, China

COMMENT ##Assembly-Data-START##

Sequencing Technology :: Sanger dideoxy sequencing

##Assembly-Data-END##

FEATURES Location/Qualifiers

source 1..1749

/organism="Influenza B virus"

/mol_type="viral cRNA"

/strain="B/Beijing/2315/2022"

/isolate="2315"

/isolation_source="nasal swab"

/host="Homo sapiens"

/db_xref="taxon:11520"

/segment="4"

/country="China: Beijing"

/collection_date="30-Aug-2022"

gene 1..1749

/gene="HA"

CDS 1..1749

/gene="HA"

/function="receptor binding and fusion protein"

/codon_start=1

/product="hemagglutinin"

/protein_id="WJE87847"

/translation="MKAIIVLLMVVTSNADRICTGITSSNSPHVVKTATQGEVNVTGV

IPLTTTPTKSHFANLKGTETRGKLCPKCLNCTDLDVALGRPKCTGKIPSARVSILHEV

RPVTSGCFPIMHDRTKIRQLPNLLRGYEHVRLSTQNVINTEDAPGGPYEIGTSGSCLN

ITNGKGFFATMAWAVPKNKTATNPLTIEVPYICTEEEDQITVWGFHSDDETQMARLYG

DSKPQKFTSSANGVTTHYVSQIGGFPNQTEDGGLPQSGRIVVDYMVQKSGKTGTITYQ

RGILLPQKVWCASGKSKVIKGSLPLIGEADCLHEKYGGLNKSKPYYTGEHAKAIGNCP

IWVKTPLKLANGTKYRPPAKLLKERGFFGAIAGFLEGGWEGMIAGWHGYTSHGAHGVA

VAADLKSTQEAINKITKNLNSLSELEVKNLQRLSGAMDELHNEILELDEKVDDLRADT

ISSQIELAVLLSNEGIINSEDEHLLALERKLKKMLGPSAVEIGNGCFETKHKCNQTCL

DRIAAGTFDAGEFSLPTFDSLNITAASLNDDGLDNHTILLYYSTAASSLAVTLMIAIF

VVYMVSRDNVSCSICL"

ORIGIN

1 atgaaggcaa taattgtact actcatggta gtaacatcca atgcagatcg aatctgcact

61 gggataacat cgtcaaactc accacatgtc gtcaaaactg ctactcaagg ggaggtcaat

121 gtgaccggtg taataccact gacaacaaca cccaccaaat ctcattttgc aaatctcaaa

181 ggaacagaaa ccagggggaa actatgccca aaatgcctaa actgcacaga tctggatgta

241 gccttgggca gaccaaaatg cacagggaaa ataccctctg caagggtttc aatactccat

301 gaagtcagac ctgttacatc tgggtgcttt cctataatgc atgatagaac aaaaattaga

361 cagctgccta accttctccg aggatacgaa catgtcaggt tatcaactca aaacgttatc

421 aatacagaag atgcaccagg aggaccctac gaaattggaa cctcagggtc ttgcctcaac

481 attaccaatg gaaaaggatt cttcgcaaca atggcttggg ccgtcccaaa aaacaaaaca

541 gcaacaaatc cattaacaat agaagtacca tacatttgta cagaagaaga agaccaaatt

601 accgtttggg ggttccactc tgacgacgag acccaaatgg caaggctcta tggggattca

661 aagccccaga agttcacctc atctgccaac ggagtgacca cacattacgt ctcacagatt

721 ggtggcttcc caaatcaaac agaagacgga ggactaccac aaagtggcag aattgttgtt

781 gattacatgg tgcaaaaatc tggaaaaaca ggaacaatta cctatcaaag aggtatttta

841 ttgcctcaaa aggtgtggtg cgcaagtggc aagagcaagg taataaaagg atccttgccc

901 ttaattggag aagcagattg cctccatgaa aaatacggtg gattaaacaa aagcaagcct

961 tactacacag gggaacatgc aaaggccata ggaaattgcc caatatgggt gaaaacaccc

1021 ttgaagctgg ccaatggaac caaatataga cctcctgcaa aactattaaa ggaaagaggt

1081 ttcttcggag ccattgctgg tttcttagag ggaggatggg aaggaatgat tgcaggttgg

1141 cacggataca catcccatgg ggcacatgga gtagcagtgg cagctgacct taagagcact

1201 caagaggcca taaacaagat aacaaaaaat ctcaactctt tgagtgagct ggaagtaaag

1261 aatcttcaaa gactaagcgg tgccatggat gaactccaca acgaaatact agaactagat

1321 gagaaagtgg atgatctcag agctgataca ataagctcac aaatagaact cgcagtcctg

1381 ctttccaatg aaggaataat aaacagcgaa gatgaacatc tcttggcgct tgaaagaaag

1441 ctgaagaaaa tgctgggccc ctctgctgta gagataggga atggatgctt tgaaaccaaa

1501 cacaagtgca accagacctg tctcgacaga atagctgctg gtacctttga tgcaggagaa

1561 ttttctctcc ccacctttga ttcactgaat attactgctg catctttaaa tgacgatgga

1621 ttggacaatc atactatact gctttactac tcaactgctg cctccagttt ggctgtaaca

1681 ctgatgatag ctatctttgt tgtttatatg gtctccagag acaatgtttc ttgctccatt

1741 tgtctataa

//

LOCUS OR145877 1749 bp cRNA linear VRL 20-JUN-2023

DEFINITION Influenza B virus (B/Beijing/2316/2022) segment 4 hemagglutinin

(HA) gene, complete cds.

ACCESSION OR145877

VERSION OR145877

KEYWORDS .

SOURCE Influenza B virus

ORGANISM Influenza B virus

Viruses; Riboviria; Orthornavirae; Negarnaviricota;

Polyploviricotina; Insthoviricetes; Articulavirales;

Orthomyxoviridae; Betainfluenzavirus; Betainfluenzavirus

influenzae.

REFERENCE 1 (bases 1 to 1749)

AUTHORS Wang,Y.

TITLE Direct Submission

JOURNAL Submitted (20-JUN-2023) Department of Infectious Diseases, Peking

University People's Hospital, Xizhimen South Street, Beijing

100044, China

COMMENT ##Assembly-Data-START##

Sequencing Technology :: Sanger dideoxy sequencing

##Assembly-Data-END##

FEATURES Location/Qualifiers

source 1..1749

/organism="Influenza B virus"

/mol_type="viral cRNA"

/strain="B/Beijing/2316/2022"

/isolate="2316"

/isolation_source="nasal swab"

/host="Homo sapiens"

/db_xref="taxon:11520"

/segment="4"

/country="China: Beijing"

/collection_date="30-Aug-2022"

gene 1..1749

/gene="HA"

CDS 1..1749

/gene="HA"

/function="receptor binding and fusion protein"

/codon_start=1

/product="hemagglutinin"

/protein_id="WJE87848"

/translation="MKAIIVLLMVVTSNADRICTGITSSNSPHVVKTATQGEVNVTGV

IPLTTTPTKSHFANLKGTETRGKLCPKCLNCTDLDVALGRPKCTGKIPSARVSILHEV

RPVTSGCFPIMHDRTKIRQLPNLLRGYEHVRLSTQNVINTKDAPGGPYEIGTSGSCLN

ISNGKGFFATMAWAVPKNKTATNPLTIEVPYICTEEEDQITVWGFHSDDETQMARLYG

DSKPQKFTSSANGVTTHYVSQIGGFPNQTEDGGLPQSGRIVVDYMVQKSGKTGTITYQ

RGILLPQKVWCASGKSKVIKGSLPLIGEADCLHEKYGGLNKSKPYYTGEHAKAIGNCP

IWVKTPLKLANGTKYRPPAKLLKERGFFGAIAGFLEGGWEGMIAGWHGYTSHGAHGVA

VAADLKSTQEAINKITKNLNSLSELEVKNLQRLSGAMDELHNEILELDEKVDDLRADT

ISSQIELAVLLSNEGIINSEDEHLLALERKLKKMLGPSAVEIGNGCFETKHKCNQTCL

DRIAAGTFDAGEFSLPTFDSLNITAASLNDDGLDNHTILLYYSTAASSLAVTLMIAIF

VVYMVSRDNVSCSICL"

ORIGIN

1 atgaaggcaa taattgtact actcatggta gtaacatcca atgcagatcg aatctgcact

61 gggataacat cgtcaaactc accacatgtc gtcaaaactg ctactcaagg ggaggtcaat

121 gtgaccggtg taataccact gacaacaaca cccaccaaat ctcattttgc aaatctcaaa

181 ggaacagaaa ccagggggaa actatgccca aaatgcctaa actgcacaga tctggatgta

241 gccttgggca gaccaaaatg cacagggaaa ataccctctg caagggtttc aatactccat

301 gaagtcagac ctgttacatc tgggtgcttt cctataatgc atgatagaac aaaaattaga

361 cagctgccta accttctccg aggatacgaa catgtcaggt tatcaactca aaacgttatc

421 aatacaaaag atgcaccagg aggaccctac gaaattggaa cctcagggtc ttgcctcaac

481 atttccaatg gaaaaggatt cttcgcaaca atggcttggg ccgtcccaaa aaacaaaaca

541 gcaacaaatc cattaacaat agaagtacca tacatttgta cagaagaaga agaccaaatt

601 accgtttggg ggttccactc tgacgacgag acccaaatgg caaggctcta tggggattca

661 aagccccaga agttcacctc atctgccaac ggagtgacca cacattacgt ctcacagatt

721 ggtggcttcc caaatcaaac agaagacgga ggactaccac aaagtggcag aattgttgtt

781 gattacatgg tgcaaaaatc tggaaaaaca ggaacaatta cctatcaaag aggtatttta

841 ttgcctcaaa aggtgtggtg cgcaagtggc aagagcaagg taataaaagg atccttgccc

901 ttaattggag aagcagattg cctccatgaa aaatacggtg gattaaacaa aagcaagcct

961 tactacacag gggaacatgc aaaggccata ggaaattgcc caatatgggt gaaaacaccc

1021 ttgaagctgg ccaatggaac caaatataga cctcctgcaa aactattaaa ggaaagaggt

1081 ttcttcggag ccattgctgg tttcttagag ggaggatggg aaggaatgat tgcaggttgg

1141 cacggataca catcccatgg ggcacatgga gtagcagtgg cagctgacct taagagcact

1201 caagaggcca taaacaagat aacaaaaaat ctcaactctt tgagtgagct ggaagtaaag

1261 aatcttcaaa gactaagcgg tgccatggat gaactccaca acgaaatact agaactagat

1321 gagaaagtgg atgatctcag agctgataca ataagctcac aaatagaact cgcagtcctg

1381 ctttccaacg aaggaataat aaacagtgag gatgaacatc tcttggcgct tgaaagaaag

1441 ctgaagaaaa tgctgggccc ctctgctgta gagataggga atggatgctt tgaaaccaaa

1501 cacaagtgca accagacctg tctcgacaga atagctgctg gtacctttga tgcaggagaa

1561 ttttctctcc ccacctttga ttcactgaat attactgctg catctttaaa tgacgatgga

1621 ttggacaatc atactatact gctttactac tcaactgctg cctccagttt ggctgtaaca

1681 ctgatgatag ctatctttgt tgtttatatg gtctccagag acaatgtttc ttgctccatt

1741 tgtctataa

//

LOCUS OR145878 1749 bp cRNA linear VRL 20-JUN-2023

DEFINITION Influenza B virus (B/Beijing/2317/2022) segment 4 hemagglutinin

(HA) gene, complete cds.

ACCESSION OR145878

VERSION OR145878

KEYWORDS .

SOURCE Influenza B virus

ORGANISM Influenza B virus

Viruses; Riboviria; Orthornavirae; Negarnaviricota;

Polyploviricotina; Insthoviricetes; Articulavirales;

Orthomyxoviridae; Betainfluenzavirus; Betainfluenzavirus

influenzae.

REFERENCE 1 (bases 1 to 1749)

AUTHORS Wang,Y.

TITLE Direct Submission

JOURNAL Submitted (20-JUN-2023) Department of Infectious Diseases, Peking

University People's Hospital, Xizhimen South Street, Beijing

100044, China

COMMENT ##Assembly-Data-START##

Sequencing Technology :: Sanger dideoxy sequencing

##Assembly-Data-END##

FEATURES Location/Qualifiers

source 1..1749

/organism="Influenza B virus"

/mol_type="viral cRNA"

/strain="B/Beijing/2317/2022"

/isolate="2317"

/isolation_source="nasal swab"

/host="Homo sapiens"

/db_xref="taxon:11520"

/segment="4"

/country="China: Beijing"

/collection_date="30-Aug-2022"

gene 1..1749

/gene="HA"

CDS 1..1749

/gene="HA"

/function="receptor binding and fusion protein"

/codon_start=1

/product="hemagglutinin"

/protein_id="WJE87849"

/translation="MKAIIVLLMVVTSNADRICTGITSSNSPHVVKTATQGEVNVTGV

IPLTTTPNKSHFANLKGTETRGKLCPKCLNCTDLDVALGRPKCTGKIPSARVSILHEV

RPVTSGCFPIMHDRTKIRQLPNLLRGYEHVRLSTQNVINTEDAPGGPYEIGTSGSCLN

ITNGKGFFATMAWAVPKNKTATNPLTIEVPYICTEEEDQITVWGFHSDDETQMARLYG

DSKPQKFTSSANGVTTHYVSQIGGFPNQTEDGGLPQSGRIVVDYMVQKSGKTGTITYQ

RGILLPQKVWCASGKSKVIKGSLPLIGEADCLHEKYGGLNKSKPYYTGEHAKAIGNCP

IWVKTPLKLANGTKYRPPAKLLKERGFFGAIAGFLEGGWEGMIAGWHGYTSHGAHGVA

VAADLKSTQEAINKITKNLNSLSELEVKNLQRLASAMDELHNEILELDEKVDDLRADT

ISSQIELAVLLSNEGIINSEDEHLLALERKLKKMLGPSAVEIGNGCFETKHKCNQTCL

DRIAAGTFDAGEFSLPTFDSLNITAASLNDDGLDNHTILLYYSTAASSLAVTLMIAIF

VVYMVSRDNVSCSICL"

ORIGIN

1 atgaaggcaa taattgtact actcatggta gtaacatcca atgcagatcg aatctgcact

61 gggataacat cgtcaaactc accacatgtc gtcaaaactg ctactcaagg ggaggtcaat

121 gtgaccggtg taataccact gacaacaaca cccaataaat ctcattttgc aaatctcaaa

181 ggaacagaaa ccagggggaa actatgccca aaatgcctaa actgcacaga tctggatgta

241 gccttgggca gaccaaaatg cacagggaaa ataccctctg caagggtttc aatactccat

301 gaagtcagac ctgttacatc tgggtgcttt cctataatgc atgatagaac aaaaattaga

361 cagctgccta accttctccg aggatacgaa catgtcaggt tatcaactca aaacgttatc

421 aatacagaag atgcaccagg aggaccctac gaaattggaa cctcagggtc ttgcctcaac

481 attaccaatg gaaaaggatt cttcgcaaca atggcttggg ccgtcccaaa aaacaaaaca

541 gcaacaaatc cattaacaat agaagtacca tacatttgta cagaagaaga agaccaaatt

601 accgtttggg ggttccactc tgacgacgag acccaaatgg caaggctcta tggggattca

661 aagccccaga agttcacctc atctgccaac ggagtgacca cacattacgt ctcacagatt

721 ggtggcttcc caaatcaaac agaagacgga ggactaccac aaagtggcag aattgttgtt

781 gattacatgg tgcaaaaatc tggaaaaaca ggaacaatta cctatcaaag aggtatttta

841 ttgcctcaaa aggtgtggtg cgcaagtggc aagagcaagg taataaaagg atccttgccc

901 ttaattggag aagcagattg cctccatgaa aaatacggtg gattaaacaa aagcaagcct

961 tactacacag gggaacatgc aaaggccata ggaaattgcc caatatgggt gaaaacaccc

1021 ttgaagctgg ccaatggaac caaatataga cctcctgcaa aactattaaa ggaaagaggt

1081 ttcttcggag ccattgctgg tttcttagag ggaggatggg aaggaatgat tgcaggttgg

1141 cacggataca catcccatgg ggcacatgga gtagcagtgg cagctgacct caagagcact

1201 caagaggcca taaacaagat aacaaaaaat ctcaactctt tgagtgagct ggaagtaaag

1261 aatcttcaaa gactggccag tgccatggat gaactccaca acgaaatact agaactagat

1321 gagaaagtgg atgatctcag agctgataca ataagctcac aaatagaact cgcagtcctg

1381 ctttccaatg aaggaataat aaacagtgaa gatgaacatc tcttggcgct tgaaagaaag

1441 ctgaagaaaa tgctgggccc ctctgctgta gagataggga atggatgctt tgaaaccaaa

1501 cacaagtgca accagacctg tctcgacaga atagctgctg gtacctttga tgcaggagaa

1561 ttttctctcc ccacctttga ttcactgaat attactgctg catctttaaa tgacgatgga

1621 ttggacaatc atactatact gctttactac tcaactgctg cctccagttt ggctgtaaca

1681 ctgatgatag ctatctttgt tgtttatatg gtctccagag acaatgtttc ttgctccatt

1741 tgtctataa

//

LOCUS OR145879 1749 bp cRNA linear VRL 20-JUN-2023

DEFINITION Influenza B virus (B/Beijing/2319/2022) segment 4 hemagglutinin

(HA) gene, complete cds.

ACCESSION OR145879

VERSION OR145879

KEYWORDS .

SOURCE Influenza B virus

ORGANISM Influenza B virus

Viruses; Riboviria; Orthornavirae; Negarnaviricota;

Polyploviricotina; Insthoviricetes; Articulavirales;

Orthomyxoviridae; Betainfluenzavirus; Betainfluenzavirus

influenzae.

REFERENCE 1 (bases 1 to 1749)

AUTHORS Wang,Y.

TITLE Direct Submission

JOURNAL Submitted (20-JUN-2023) Department of Infectious Diseases, Peking

University People's Hospital, Xizhimen South Street, Beijing

100044, China

COMMENT ##Assembly-Data-START##

Sequencing Technology :: Sanger dideoxy sequencing

##Assembly-Data-END##

FEATURES Location/Qualifiers

source 1..1749

/organism="Influenza B virus"

/mol_type="viral cRNA"

/strain="B/Beijing/2319/2022"

/isolate="2319"

/isolation_source="nasal swab"

/host="Homo sapiens"

/db_xref="taxon:11520"

/segment="4"

/country="China: Beijing"

/collection_date="30-Aug-2022"

gene 1..1749

/gene="HA"

CDS 1..1749

/gene="HA"

/function="receptor binding and fusion protein"

/codon_start=1

/product="hemagglutinin"

/protein_id="WJE87850"

/translation="MKAIIVLLMVVTSNADRICTGITSSNSPHVVKTATQGEVNVTGV

IPLTTTPTKSHFANLKGTETRGKLCPKCLNCTDLDVALGRPKCTGKIPSARVSILHEV

RPVTSGCFPIMHDRTKIRQLPNLLRGYEHVRLSTQNVINTEDAPGGPYEIGTSGSCLN

ITNGKGFFATMAWAVPKNKTATNPLTIEVPYICTEEEDQITVWGFHSDDETQMARLYG

DSKPQKFTSSANGVTTHYVSQIGGFPNQTEDGGLPQSGRIVVDYMVQKSGKTGTITYQ

RGILLPQKVWCASGKSKVIKGSLPLIGEADCLHEKYGGLNKSKPYYTGEHAKAIGNCP

IWVKTPLKLANGTKYRPPAKLLKERGFFGAIAGFLEGGWEGMIAGWHGYTSHGAHGVA

VAADLKSTQEAINKITKNLNSLSELEVKNLQRLSGAMDELHNEILELDEKVDDLRADT

ISSQIELAVLLSNEGIINSEDEHLLALERKLKKMLGPSAVEIGNGCFETKHKCNQTCL

DRIAAGTFDAGEFSLPTFDSLNITAASLNDDGLDNHTILLYYSTAASSLAVTLMIAIF

VVYMVSRDNVSCSICL"

ORIGIN

1 atgaaggcaa taattgtact actcatggta gtaacatcca atgcagatcg aatctgcact

61 gggataacat cgtcaaactc accacatgtc gtcaaaactg ctactcaagg ggaggtcaat

121 gtgaccggtg taataccact gacaacaaca cccaccaaat ctcattttgc aaatctcaaa

181 ggaacagaaa ccagggggaa actatgccca aaatgcctaa actgcacaga tctggatgta

241 gccttgggca gaccaaaatg cacagggaaa ataccctctg caagggtttc aatactccat

301 gaagtcagac ctgttacatc tgggtgcttt cctataatgc atgatagaac aaaaattaga

361 cagctgccta accttctccg aggatacgaa catgtcaggt tatcaactca aaacgttatc

421 aatacagaag atgcaccagg aggaccctac gaaattggaa cctcagggtc ttgcctcaac

481 attaccaatg gaaaaggatt cttcgcaaca atggcttggg ccgtcccaaa aaacaaaaca

541 gcaacaaatc cattaacaat agaagtacca tacatttgta cagaagaaga agaccaaatt

601 accgtttggg ggttccactc tgacgacgag acccaaatgg caaggctcta tggggattca

661 aagccccaga agttcacctc atctgccaac ggagtgacca cacattacgt ctcacagatt

721 ggtggcttcc caaatcaaac agaagacgga ggactaccac aaagtggcag aattgttgtt

781 gattacatgg tgcaaaaatc tggaaaaaca ggaacaatta cctatcaaag aggtatttta

841 ttgcctcaaa aggtgtggtg cgcaagtggc aagagcaagg taataaaagg atccttgccc

901 ttaattggag aagcagattg cctccatgaa aaatacggtg gattaaacaa aagcaagcct

961 tactacacag gggaacatgc aaaggccata ggaaattgcc caatatgggt gaaaacaccc

1021 ttgaagctgg ccaatggaac caaatataga cctcctgcaa aactattaaa ggaaagaggt

1081 ttcttcggag ccattgctgg tttcttagag ggaggatggg aaggaatgat tgcaggttgg

1141 cacggataca catcccatgg ggcacatgga gtagcagtgg cagctgacct taagagcact

1201 caagaggcca taaacaagat aacaaaaaat ctcaactctt tgagtgagct ggaagtaaag

1261 aatcttcaaa gactaagcgg tgccatggat gaactccaca acgaaatact agaactagat

1321 gagaaagtgg atgatctcag agctgataca ataagctcac aaatagaact cgcagtcctg

1381 ctttccaatg aaggaataat aaacagtgaa gatgaacatc tcttggcgct tgaaagaaag

1441 ctgaagaaaa tgctgggccc ctctgctgta gagataggga atggatgctt tgaaaccaaa

1501 cacaagtgca accagacctg tctcgacaga atagctgctg gtacctttga tgcaggagaa

1561 ttttctctcc ccacctttga ttcactgaat attactgctg catctttaaa tgacgatgga

1621 ttggacaatc atactatact gctttactac tcaactgctg cctccagttt ggctgtaaca

1681 ctgatgatag ctatctttgt tgtttatatg gtctccagag acaatgtttc ttgctccatt

1741 tgtctataa

//

LOCUS OR145880 1749 bp cRNA linear VRL 20-JUN-2023

DEFINITION Influenza B virus (B/Beijing/2320/2022) segment 4 hemagglutinin

(HA) gene, complete cds.

ACCESSION OR145880

VERSION OR145880

KEYWORDS .

SOURCE Influenza B virus

ORGANISM Influenza B virus

Viruses; Riboviria; Orthornavirae; Negarnaviricota;

Polyploviricotina; Insthoviricetes; Articulavirales;

Orthomyxoviridae; Betainfluenzavirus; Betainfluenzavirus

influenzae.

REFERENCE 1 (bases 1 to 1749)

AUTHORS Wang,Y.

TITLE Direct Submission

JOURNAL Submitted (20-JUN-2023) Department of Infectious Diseases, Peking

University People's Hospital, Xizhimen South Street, Beijing

100044, China

COMMENT ##Assembly-Data-START##

Sequencing Technology :: Sanger dideoxy sequencing

##Assembly-Data-END##

FEATURES Location/Qualifiers

source 1..1749

/organism="Influenza B virus"

/mol_type="viral cRNA"

/strain="B/Beijing/2320/2022"

/isolate="2320"

/isolation_source="nasal swab"

/host="Homo sapiens"

/db_xref="taxon:11520"

/segment="4"

/country="China: Beijing"

/collection_date="30-Aug-2022"

gene 1..1749

/gene="HA"

CDS 1..1749

/gene="HA"

/function="receptor binding and fusion protein"

/codon_start=1

/product="hemagglutinin"

/protein_id="WJE87851"

/translation="MKAIIVLLMVVTSNADRICTGITSSNSPHVVKTATQGEVNVTGV

IPLTTTPTKSHFANLKGTETRGKLCPKCLNCTDLDVALGRPKCTGKIPSARVSILHEV

RPVTSGCFPIMHDRTKIRQLPNLLRGYEHVRLSTQNVINTEDAPGGPYEIGTSGSCLN

ITNGKGFFATMAWAVPKNKTATNPLTIEVPYICTEEEDQITVWGFHSDDETQMARLYG

DSKPQKFTSSANGVTTHYVSQIGGFPNQTEDGGLPQSGRIVVDYMVQKSGKTGTITYQ

RGILLPQKVWCASGKSKVIKGSLPLIGEADCLHEKYGGLNKSKPYYTGEHAKAIGNCP

IWVKTPLKLANGTKYRPPAKLLKERGFFGAIAGFLEGGWEGMIAGWHGYTSHGAHGVA

VAADLKSTQEAINKITKNLNSLSELEVKNLQRLSGAMDELHNEILELDEKVDDLRADT

ISSQIELAVLLSNEGIINSEDEHLLALERKLKKMLGPSAVEIGNGCFETKHKCNQTCL

DRIAAGTFDAGEFSLPTFDSLNITAASLNDDGLDNHTILLYYSTAASSLAVTLMIAIF

VVYMVSRDNVSCSICL"

ORIGIN

1 atgaaggcaa taattgtact actcatggta gtaacatcca atgcagatcg aatctgcact

61 gggataacat cgtcaaactc accacatgtc gtcaaaactg ctactcaagg ggaggtcaat

121 gtgaccggtg taataccact gacaacaaca cccaccaaat ctcattttgc aaatctcaaa

181 ggaacagaaa ccagggggaa actatgccca aaatgcctaa actgcacaga tctggatgta

241 gccttgggca gaccaaaatg cacagggaaa ataccctctg caagggtttc aatactccat

301 gaagtcagac ctgttacatc tgggtgcttt cctataatgc atgatagaac aaaaattaga

361 cagctgccta accttctccg aggatacgaa catgtcaggt tatcaactca aaacgttatc

421 aatacagaag atgcaccagg aggaccctac gaaattggaa cctcagggtc ttgcctcaac

481 attaccaatg gaaaaggatt cttcgcaaca atggcttggg ccgtcccaaa aaacaaaaca

541 gcaacaaatc cattaacaat agaagtacca tacatttgta cagaagaaga agaccaaatt

601 accgtttggg ggttccactc tgacgacgag acccaaatgg caaggctcta tggggattca

661 aagccccaga agttcacctc atctgccaac ggagtgacca cacattacgt ctcacagatt

721 ggtggcttcc caaatcaaac agaagacgga ggactaccac aaagtggcag aattgttgtt

781 gattacatgg tgcaaaaatc tggaaaaaca ggaacaatta cctatcaaag aggtatttta

841 ttgcctcaaa aggtgtggtg cgcaagtggc aagagcaagg taataaaagg atccttgccc

901 ttaattggag aagcagattg cctccatgaa aaatacggtg gattaaacaa aagcaagcct

961 tactacacag gggaacatgc aaaggccata ggaaattgcc caatatgggt gaaaacaccc

1021 ttgaagctgg ccaatggaac caaatataga cctcctgcaa aactattaaa ggaaagaggt

1081 ttcttcggag ccattgctgg tttcttagag ggaggatggg aaggaatgat tgcaggttgg

1141 cacggataca catcccatgg ggcacatgga gtagcagtgg cagctgacct taagagcact

1201 caagaggcca taaacaagat aacaaaaaat ctcaactctt tgagtgagct ggaagtaaag

1261 aatcttcaaa gactaagcgg tgccatggat gaactccaca acgaaatact agaactagat

1321 gagaaagtgg atgatctcag agctgataca ataagctcac aaatagaact cgcagtcctg

1381 ctttccaatg aaggaataat aaacagtgaa gatgaacatc tcttggcgct tgaaagaaag

1441 ctgaagaaaa tgctgggccc ctctgctgta gagataggga atggatgctt tgaaaccaaa

1501 cacaagtgca accagacctg tctcgacaga atagctgctg gtacctttga tgcaggagaa

1561 ttttctctcc ccacctttga ttcactgaat attactgctg catctttaaa tgacgatgga

1621 ttggacaatc atactatact gctttactac tcaactgctg cctccagttt ggctgtaaca

1681 ctgatgatag ctatctttgt tgtttatatg gtctccagag acaatgtttc ttgctccatt

1741 tgtctataa

//

LOCUS OR145881 1749 bp cRNA linear VRL 20-JUN-2023

DEFINITION Influenza B virus (B/Beijing/2321/2022) segment 4 hemagglutinin

(HA) gene, complete cds.

ACCESSION OR145881

VERSION OR145881

KEYWORDS .

SOURCE Influenza B virus

ORGANISM Influenza B virus

Viruses; Riboviria; Orthornavirae; Negarnaviricota;

Polyploviricotina; Insthoviricetes; Articulavirales;

Orthomyxoviridae; Betainfluenzavirus; Betainfluenzavirus

influenzae.

REFERENCE 1 (bases 1 to 1749)

AUTHORS Wang,Y.

TITLE Direct Submission

JOURNAL Submitted (20-JUN-2023) Department of Infectious Diseases, Peking

University People's Hospital, Xizhimen South Street, Beijing

100044, China

COMMENT ##Assembly-Data-START##

Sequencing Technology :: Sanger dideoxy sequencing

##Assembly-Data-END##

FEATURES Location/Qualifiers

source 1..1749

/organism="Influenza B virus"

/mol_type="viral cRNA"

/strain="B/Beijing/2321/2022"

/isolate="2321"

/isolation_source="nasal swab"

/host="Homo sapiens"

/db_xref="taxon:11520"

/segment="4"

/country="China: Beijing"

/collection_date="30-Aug-2022"

gene 1..1749

/gene="HA"

CDS 1..1749

/gene="HA"

/function="receptor binding and fusion protein"

/codon_start=1

/product="hemagglutinin"

/protein_id="WJE87852"

/translation="MKAIIVLLMVVTSNADRICTGITSSNSPHVVKTATQGEVNVTGV

IPLTTTPTKSHFANLKGTETRGKLCPKCLNCTDLDVALGRPKCTGKIPSARVSILHEV

RPVTSGCFPIMHDRTKIRQLPNLLRGYEHVRLSTQNVINTEDAPGGPYEIGTSGSCLN

ITNGKGFFATMAWAVPKNKTATNPLTIEVPYICTEEEDQITVWGFHSDDETQMARLYG

DSKPQKFTSSANGVTTHYVSQIGGFPNQTEDGGLPQSGRIVVDYMVQKSGKTGTITYQ

RGILLPQKVWCASGKSKVIKGSLPLIGEADCLHEKYGGLNKSKPYYTGEHAKAIGNCP

IWVKTPLKLANGTKYRPPAKLLKERGFFGAIAGFLEGGWEGMIAGWHGYTSHGAHGVA

VAADLKSTQEAINKITKNLNSLSELEVKNLQRLSGAMDELHNEILELDEKVDDLRADT

ISSQIELAVLLSNEGIINSEDEHLLALERKLKKMLGPSAVEIGNGCFETKHKCNQTCL

DRIAAGTFDAGEFSLPTFDSLNITAAYLNDDGLDNHTILLYYSTAASSLAVTLMIAIF

VVYMVSRDNVSCSICL"

ORIGIN

1 atgaaggcaa taattgtact actcatggta gtaacatcca atgcagatcg aatctgcact

61 gggataacat cgtcaaactc accacatgtc gtcaaaactg ctactcaagg ggaggtcaat

121 gtgaccggtg taataccact gacaacaaca cccaccaaat ctcattttgc aaatctcaaa

181 ggaacagaaa ccagggggaa actatgccca aaatgcctaa actgcacaga tctggatgta

241 gccttgggca gaccaaaatg cacagggaaa ataccctctg caagggtttc aatactccat

301 gaagtcagac ctgttacatc tgggtgcttt cctataatgc atgatagaac aaaaattaga

361 cagctgccta accttctccg aggatacgaa catgtcaggt tatcaactca aaacgttatc

421 aatacagaag atgcaccagg aggaccctac gaaattggaa cctcagggtc ttgcctcaac

481 attaccaatg gaaaaggatt cttcgcaaca atggcttggg ccgtcccaaa aaacaaaaca

541 gcaacaaatc cattaacaat agaagtacca tacatttgta cagaagaaga agaccaaatt

601 accgtttggg ggttccactc tgacgacgag acccaaatgg caaggctcta tggggattca

661 aagccccaga agttcacctc atctgccaac ggagtgacca cacattacgt ctcacagatt

721 ggtggcttcc caaatcaaac agaagacgga ggactaccac aaagtggcag aattgttgtt

781 gattacatgg tgcaaaaatc tggaaaaaca ggaacaatta cctatcaaag aggtatttta

841 ttgcctcaaa aggtgtggtg cgcaagtggc aagagcaagg taataaaagg atccttgccc

901 ttaattggag aagcagattg cctccatgaa aaatacggtg gattaaacaa aagcaagcct

961 tactacacag gggaacatgc aaaggccata ggaaattgcc caatatgggt gaaaacaccc

1021 ttgaagctgg ccaatggaac caaatataga cctcctgcaa aactattaaa ggaaagaggt

1081 ttcttcggag ccattgctgg tttcttagag ggaggatggg aaggaatgat tgcaggttgg

1141 cacggataca catcccatgg ggcacatgga gtagcagtgg cagctgacct taagagcact

1201 caagaggcca taaacaagat aacaaaaaat ctcaactctt tgagtgagct agaagtaaag

1261 aatcttcaaa gactaagcgg tgccatggat gaactccaca acgaaatact agaactagat

1321 gagaaagtgg atgatctcag agctgataca ataagctcac aaatagaact cgcagtcctg

1381 ctttccaatg aaggaataat aaacagtgaa gatgaacatc tcttggcgct tgaaagaaag

1441 ctgaagaaaa tgctgggccc ctctgctgta gagataggga atggatgctt tgaaaccaaa

1501 cacaagtgca accagacctg tctcgacaga atagctgctg gtacctttga tgcaggagaa

1561 ttttctctcc ccacctttga ttcactgaat attactgctg catatttaaa tgacgatgga

1621 ttggacaatc atactatact gctttactac tcaactgctg cctccagttt ggctgtaaca

1681 ctgatgatag ctatctttgt tgtttatatg gtctccagag acaatgtttc ttgctccatt

1741 tgtctataa

//

LOCUS OR145882 1749 bp cRNA linear VRL 20-JUN-2023

DEFINITION Influenza B virus (B/Beijing/2322/2022) segment 4 hemagglutinin

(HA) gene, complete cds.

ACCESSION OR145882

VERSION OR145882

KEYWORDS .

SOURCE Influenza B virus

ORGANISM Influenza B virus

Viruses; Riboviria; Orthornavirae; Negarnaviricota;

Polyploviricotina; Insthoviricetes; Articulavirales;

Orthomyxoviridae; Betainfluenzavirus; Betainfluenzavirus

influenzae.

REFERENCE 1 (bases 1 to 1749)

AUTHORS Wang,Y.

TITLE Direct Submission

JOURNAL Submitted (20-JUN-2023) Department of Infectious Diseases, Peking

University People's Hospital, Xizhimen South Street, Beijing

100044, China

COMMENT ##Assembly-Data-START##

Sequencing Technology :: Sanger dideoxy sequencing

##Assembly-Data-END##

FEATURES Location/Qualifiers

source 1..1749

/organism="Influenza B virus"

/mol_type="viral cRNA"

/strain="B/Beijing/2322/2022"

/isolate="2322"

/isolation_source="nasal swab"

/host="Homo sapiens"

/db_xref="taxon:11520"

/segment="4"

/country="China: Beijing"

/collection_date="30-Aug-2022"

gene 1..1749

/gene="HA"

CDS 1..1749

/gene="HA"

/function="receptor binding and fusion protein"

/codon_start=1

/product="hemagglutinin"

/protein_id="WJE87853"

/translation="MKAIIVLLMVVTSNADRICTGITSSNSPHVVKTATQGEVNVTGV

IPLTTTPTKSHFANLKGTETRGKLCPKCLNCTDLDVALGRPKCTGKIPSARVSILHEV

RPVTSGCFPIMHDRTKIRQLPNLLRGYEHVRLSTQNVINTEDAPGGPYEIGTSGSCLN

ITNGKGFFATMAWAVPKNKTATNPLTIEVPYICTEEEDQITVWGFHSDDETQMARLYG

DSKPQKFTSSGNGVTTHYVSQIGGFPNQTEDGGLPQSGRIVVDYMVQKSGKTGTITYQ

RGILLPQKVWCASGKSKVIKGSLPLIGEADCLHEKYGGLNKSKPYYTGEHAKAIGNCP

IWVKTPLKLANGTKYRPPAKLLKERGFFGAIAGFLEGGWEGMIAGWHGYTSHGAHGVA

VAADLKSTQEAINKITKNLNSLSELEVKNLQRLSGAMDELHNEILELDEKVDDLRADT

ISSQIELAVLLSNEGIINSEDEHLLALERKLKKMLGPSAVEIGNGCFETKHKCNQTCL

DRIAAGTFDAGEFSLPTFDSLNITAASLNNDGLDNHTILLYYSTAASSLAVTLMIAIF

VVYMVSRDNVSCSICL"

ORIGIN

1 atgaaggcaa taattgtact actcatggta gtaacatcca atgcagatcg aatctgcact

61 gggataacat cgtcaaactc accacatgtc gtcaaaactg ctactcaagg ggaggtcaat

121 gtgaccggtg taataccact gacaacaaca cccaccaaat ctcattttgc aaatctcaaa

181 ggaacagaaa ccagggggaa actatgccca aaatgcctaa actgcacaga tctggatgta

241 gccttgggca gaccaaaatg cacagggaaa ataccctctg caagggtttc aatactccat

301 gaagtcagac ctgttacatc tgggtgcttt cctataatgc atgatagaac aaaaattaga

361 cagctgccta accttctccg aggatacgaa catgtcaggt tatcaactca aaacgttatc

421 aatacagaag atgcaccagg aggaccctac gaaattggaa cctcagggtc ttgcctcaac

481 attaccaatg gaaaaggatt cttcgcaaca atggcttggg ccgtcccaaa aaacaaaaca

541 gcaacaaatc cattaacaat agaagtacca tacatttgta cagaagaaga agaccaaatt

601 accgtttggg ggttccactc tgacgacgag acccaaatgg caaggctcta tggggattca

661 aagccccaga agttcacctc atctggcaac ggagtgacca cacattacgt ctcacagatt

721 ggtggcttcc caaatcaaac agaagacgga ggactaccac aaagtggcag aattgttgtt

781 gattacatgg tgcaaaaatc tggaaaaaca ggaacaatta cctatcaaag aggtatttta

841 ttgcctcaaa aggtgtggtg cgcaagtggc aagagcaagg taataaaagg atccttgccc

901 ttaattggag aagcagattg cctccatgaa aaatacggtg gattaaacaa aagcaagcct

961 tactacacag gggaacatgc aaaggccata ggaaattgcc caatatgggt gaaaacaccc

1021 ttgaagctgg caaatggaac caaatataga cctcctgcaa aactattaaa ggaaagaggt

1081 ttcttcggag ccattgctgg tttcttagag ggaggatggg aaggaatgat tgcaggctgg

1141 cacggataca catcccatgg ggcacatgga gtagcagtgg cagctgacct taagagcact

1201 caagaggcca taaacaagat aacaaaaaat ctcaactctt tgagtgagct ggaagtaaag

1261 aatcttcaaa gactaagcgg tgccatggat gaacttcaca acgaaatact agaactagat

1321 gagaaagtgg atgatctcag agctgataca ataagctcac aaatagaact cgcagtcctg

1381 ctttccaatg aaggaataat aaacagtgaa gatgaacatc tcttggcgct tgaaagaaag

1441 ctgaagaaaa tgctgggccc ctctgctgta gagataggga atggatgctt tgaaaccaaa

1501 cacaagtgca accagacctg tctcgacaga atagctgctg gtacctttga tgcaggagaa

1561 ttttctctcc ccacctttga ttcactgaat attactgctg catctttaaa taacgatgga

1621 ttggacaatc atactatact gctttactac tcaactgctg cctccagttt ggctgtaaca

1681 ctgatgatag ctatctttgt tgtttatatg gtctccagag acaatgtttc ttgctccatt

1741 tgtctataa

//

LOCUS OR145883 1749 bp cRNA linear VRL 20-JUN-2023

DEFINITION Influenza B virus (B/Beijing/2324/2022) segment 4 hemagglutinin

(HA) gene, complete cds.

ACCESSION OR145883

VERSION OR145883

KEYWORDS .

SOURCE Influenza B virus

ORGANISM Influenza B virus

Viruses; Riboviria; Orthornavirae; Negarnaviricota;

Polyploviricotina; Insthoviricetes; Articulavirales;

Orthomyxoviridae; Betainfluenzavirus; Betainfluenzavirus

influenzae.

REFERENCE 1 (bases 1 to 1749)

AUTHORS Wang,Y.

TITLE Direct Submission

JOURNAL Submitted (20-JUN-2023) Department of Infectious Diseases, Peking

University People's Hospital, Xizhimen South Street, Beijing

100044, China

COMMENT ##Assembly-Data-START##

Sequencing Technology :: Sanger dideoxy sequencing

##Assembly-Data-END##

FEATURES Location/Qualifiers

source 1..1749

/organism="Influenza B virus"

/mol_type="viral cRNA"

/strain="B/Beijing/2324/2022"

/isolate="2324"

/isolation_source="nasal swab"

/host="Homo sapiens"

/db_xref="taxon:11520"

/segment="4"

/country="China: Beijing"

/collection_date="30-Aug-2022"

gene 1..1749

/gene="HA"

CDS 1..1749

/gene="HA"

/function="receptor binding and fusion protein"

/codon_start=1

/product="hemagglutinin"

/protein_id="WJE87854"

/translation="MKAIIVLLMVVTSNADRICTGITSSNSPHVVKTATQGEVNVTGV

IPLTTTPTKSHFANLKGTETRGKLCPKCLNCTDLDVALGRPKCTGKIPSARVSILHEV

RPVTSGCFPIMHDRTKIRQLPNLLRGYEHVRLSTQNVINTEDAPGGPYEIGTSGSCLN

ITNGKGFFTTMAWAVPKNKTATNPLTIEVPYICTEEEDQITVWGFHSDDETQMARLYG

DSKPQKFTSSANGVTTHYVSQIGGFPNQTEDGGLPQSGRIVVDYMVQKSGKTGTITYQ

RGILLPQKVWCASGKSKVIKGSLPLIGEADCLHEKYGGLNKSKPYYTGEHAKAIGNCP

IWVKTPLKLANGTKYRPPAKLLKERGFFGAIAGFLEGGWEGMIAGWHGYTSHGAHGVA

VAADLKSTQEAINKITKNLNSLSELEVKNLQRLRSAMDELHNEILELDEKVDDLRADT

ISSQIELAVLLSNEGIINSEDEHLLALERKLKKMLGPSAVEIGNGCFETKHKCNQTCL

DRIAAGTFDAGEFSLPTFDSLNITAASLNDDGLDNHTILLYYSTAASSLAVTLMIAIF

VVYMVSRDNVSCSICL"

ORIGIN

1 atgaaggcaa taattgtact actcatggta gtaacatcca atgcagatcg aatctgcact

61 gggataacat cgtcaaactc accacatgtc gtcaaaactg ctactcaagg ggaggtcaat

121 gtgaccggtg taataccact gacaacaaca cccaccaaat ctcattttgc aaatctcaaa

181 ggaacagaaa ccagggggaa actatgccca aaatgcctaa actgcacaga tctggatgta

241 gccttgggca gaccaaaatg cacagggaaa ataccctctg caagggtttc aatactccat

301 gaagtcagac ctgttacatc tgggtgcttt cctataatgc atgatagaac aaaaattaga

361 cagctgccta accttctccg aggatacgaa catgtcaggt tatcaactca aaacgttatc

421 aatacagaag atgcaccagg aggaccctac gaaattggaa cctcagggtc ttgcctcaac

481 attaccaatg gaaaaggatt cttcacaaca atggcttggg ccgtcccaaa aaacaaaaca

541 gcaacaaatc cattaacaat agaagtacca tacatttgta cagaagaaga agaccaaatt

601 accgtttggg ggttccactc tgacgacgag acccaaatgg caaggctcta tggggattca

661 aagccccaga agttcacctc atctgccaac ggagtgacca cacattacgt ctcacagatt

721 ggtggcttcc caaatcaaac agaagacgga ggactaccac aaagtggcag aattgttgtt

781 gattacatgg tgcaaaaatc tggaaaaaca ggaacaatta cctatcaaag aggtatttta

841 ttgcctcaaa aggtgtggtg cgcaagtggc aagagcaagg taataaaagg atccttgccc

901 ttaattggag aagcagattg cctccatgaa aaatacggtg gattaaacaa aagcaagcct

961 tactacacag gggaacatgc aaaggccata gggaattgcc caatatgggt gaaaacaccc

1021 ttgaagctgg ccaatggaac caaatataga cctcctgcaa aactattaaa ggaaagaggt

1081 ttcttcggag ccattgctgg tttcttagag ggaggatggg aaggaatgat tgcaggttgg

1141 cacggataca catcccatgg ggcacatgga gtagcagtgg cagctgacct taagagcact

1201 caagaggcca taaacaagat aacaaaaaat ctcaactctt tgagtgagct ggaagtaaag

1261 aatcttcaaa gactacgcag tgccatggat gaactccaca acgaaatact agaactagat

1321 gagaaagtgg atgatctcag agctgataca ataagctcac aaatagaact cgcagtcctg

1381 ctttccaatg aaggaataat aaacagtgaa gatgaacatc tcttggcgct tgaaagaaag

1441 ctgaagaaaa tgctgggccc ctctgctgta gagataggga atggatgctt tgaaaccaaa

1501 cacaagtgca accagacctg tctcgacaga atagctgctg gtacctttga tgcaggagaa

1561 ttttctctcc ccacctttga ttcactgaat attactgctg catctttaaa tgacgatgga

1621 ttggacaatc atactatact gctttactac tcaactgctg cctccagttt ggctgtaaca

1681 ctgatgatag ctatctttgt tgtttatatg gtctccagag acaatgtttc ttgctccatt

1741 tgtctataa

//

LOCUS OR145884 1749 bp cRNA linear VRL 20-JUN-2023

DEFINITION Influenza B virus (B/Beijing/2325/2022) segment 4 hemagglutinin

(HA) gene, complete cds.

ACCESSION OR145884

VERSION OR145884

KEYWORDS .

SOURCE Influenza B virus

ORGANISM Influenza B virus

Viruses; Riboviria; Orthornavirae; Negarnaviricota;

Polyploviricotina; Insthoviricetes; Articulavirales;

Orthomyxoviridae; Betainfluenzavirus; Betainfluenzavirus

influenzae.

REFERENCE 1 (bases 1 to 1749)

AUTHORS Wang,Y.

TITLE Direct Submission

JOURNAL Submitted (20-JUN-2023) Department of Infectious Diseases, Peking

University People's Hospital, Xizhimen South Street, Beijing

100044, China

COMMENT ##Assembly-Data-START##

Sequencing Technology :: Sanger dideoxy sequencing

##Assembly-Data-END##

FEATURES Location/Qualifiers

source 1..1749

/organism="Influenza B virus"

/mol_type="viral cRNA"

/strain="B/Beijing/2325/2022"

/isolate="2325"

/isolation_source="nasal swab"

/host="Homo sapiens"

/db_xref="taxon:11520"

/segment="4"

/country="China: Beijing"

/collection_date="30-Aug-2022"

gene 1..1749

/gene="HA"

CDS 1..1749

/gene="HA"

/function="receptor binding and fusion protein"

/codon_start=1

/product="hemagglutinin"

/protein_id="WJE87855"

/translation="MKAIIVLLMVVTSNADRICTGITSSNSPHVVKTATQGEVNVTGV

IPLTTTPTKSHFANLKGTETRGKLCPKCLNCTDLDVALGRPKCTGKIPSAKVSILHEV

RPVTSGCFPMMHDRTKIRQLPNLLRGYEHVRLSTHNVINAEDAPGGPYEIGTSGSCPN

ITNGKGFFATMAWAVPKNKTATNPLTIEVPYICTEEEDQITVWGFHSDDETQMAKLYG

DSKPQKFTSSANGMTTHYVSQIGGFPNQTEDGGLQQSGRIVVDYMVQKSGKTGTITYQ

RGILLPQKVWCASGKSKVIKGSLPLVGEADCLHEKYGGLNKSKPYYTGEHAKAIGNCP

IWVKTPLKLANGTKYRPPAKLLKERGFFGAIAGFLEGGWEGMIAGWHGYTSHGAHGVA

VAADLKSTQEAINKITKNLNSLSELEVKNLQRLRSAMDELHNEILELDEKVDDLRADT

ISSQIELAVLLSNEGIINSEDEHLLALERKLKKMLGPSAVEIGNGCFETKHKCNQTCL

DRIAAGTFDAGEFSLPTFDSLNITAASLNDDGLDNHTILLYYSTAASSLAVTLMIAIF

VVYMVSRDNVSCSICL"

ORIGIN

1 atgaaggcaa taattgtact actcatggta gtaacatcca atgcagatcg aatctgcact

61 gggataacat cgtcaaactc accacatgtc gtcaaaactg ctactcaagg ggaggtcaat

121 gtgaccggtg taataccact gacaacaaca cccaccaaat ctcattttgc aaatctcaaa

181 ggaacagaaa ccagggggaa actatgccca aaatgcctca actgcacaga tctggatgta

241 gccttgggca gaccaaaatg cacagggaaa ataccctctg caaaggtttc aatactccat

301 gaagtcagac ctgttacatc tgggtgcttt cccatgatgc atgatagaac aaaaattaga

361 cagctgccta accttctccg aggatacgaa catgtcagat tatcaactca caacgttatc

421 aatgcagaag atgcaccagg aggaccctac gaaattggaa cctcagggtc ttgccctaac

481 attaccaatg gaaaaggatt cttcgcaaca atggcttggg ccgtcccaaa aaacaaaaca

541 gcaacaaatc cattaacaat agaagtacca tacatttgta cagaagaaga agaccaaatt

601 accgtttggg ggttccactc tgacgacgag acccaaatgg caaagctcta tggggactca

661 aagccccaga agttcacctc atctgccaac ggaatgacca cacattacgt ttcacagatt

721 ggtggcttcc caaatcaaac agaagacgga ggactacaac aaagtggcag aattgttgtt

781 gattacatgg tgcaaaaatc tggaaaaaca ggaacaatta cctatcaaag aggtatttta

841 ttgccccaaa aggtgtggtg cgcaagtggc aagagcaagg taataaaagg atccttgccc

901 ttagttggag aagcagattg cctccatgaa aaatacggtg gattaaacaa aagcaagcct

961 tactacacag gggaacatgc aaaggccata ggaaattgcc caatatgggt gaaaacaccc

1021 ttgaagctgg ccaatggaac caaatataga cctcctgcaa aactattaaa ggaaagaggt

1081 ttcttcggag ccattgctgg tttcttagag ggaggatggg aaggaatgat tgcaggttgg

1141 cacggataca catcccatgg ggcacatgga gtagcggtgg cagctgacct taagagcact

1201 caagaggcca taaacaagat aacaaaaaat ctcaactctt tgagtgagct ggaagtaaag

1261 aatcttcaaa gactacgcag tgccatggat gaactccata acgaaatact agaactagat

1321 gagaaagtgg atgatctcag agctgataca ataagctcac aaatagaact cgcagtcctg

1381 ctttccaatg aaggaataat aaacagtgaa gatgaacatc tcttggcgct tgaaagaaag

1441 ctgaagaaaa tgctgggccc ctctgctgta gagataggga atggatgctt tgaaaccaaa

1501 cacaagtgca accagacctg tctcgacaga atagctgctg gtacctttga tgcaggagaa

1561 ttttctctcc ccacctttga ttcactgaat attactgctg catctttaaa tgacgatgga

1621 ttggacaatc atactatact gctttactac tcaactgctg cctccagttt ggctgtaaca

1681 ctgatgatag ctatctttgt tgtttatatg gtctccagag acaatgtttc ttgctccatt

1741 tgtctataa

//

LOCUS OR145885 1749 bp cRNA linear VRL 20-JUN-2023

DEFINITION Influenza B virus (B/Beijing/2326/2022) segment 4 hemagglutinin

(HA) gene, complete cds.

ACCESSION OR145885

VERSION OR145885

KEYWORDS .

SOURCE Influenza B virus

ORGANISM Influenza B virus

Viruses; Riboviria; Orthornavirae; Negarnaviricota;

Polyploviricotina; Insthoviricetes; Articulavirales;

Orthomyxoviridae; Betainfluenzavirus; Betainfluenzavirus

influenzae.

REFERENCE 1 (bases 1 to 1749)

AUTHORS Wang,Y.

TITLE Direct Submission

JOURNAL Submitted (20-JUN-2023) Department of Infectious Diseases, Peking

University People's Hospital, Xizhimen South Street, Beijing

100044, China

COMMENT ##Assembly-Data-START##

Sequencing Technology :: Sanger dideoxy sequencing

##Assembly-Data-END##

FEATURES Location/Qualifiers

source 1..1749

/organism="Influenza B virus"

/mol_type="viral cRNA"

/strain="B/Beijing/2326/2022"

/isolate="2326"

/isolation_source="nasal swab"

/host="Homo sapiens"

/db_xref="taxon:11520"

/segment="4"

/country="China: Beijing"

/collection_date="30-Aug-2022"

gene 1..1749

/gene="HA"

CDS 1..1749

/gene="HA"

/function="receptor binding and fusion protein"

/codon_start=1

/product="hemagglutinin"

/protein_id="WJE87856"

/translation="MKAIIVLLMVVTSNADRICTGITSSNSPHVVKTATQGEVNVTGV

IPLTTTPTKSHFANLKGTETRGKLCPKCLNCTDLDVALGRPKCTGKIPSARVSILHEV

RPVTSGCFPIMHDRTKIRQLPNLLRGYEHVRLSTQNVINTEDAPGGPYEIGTSGSCLN

ITNGKGFFATMAWAVPKNKTATNPLTIEVPYICTEEEDQITVWGFHSDDETQMARLYG

DSKPQKFTSSANGVTTHYVSQIGGFPNQTEDGGLPQSGRIVVDYMVQKSGKTGTITYQ

RGILLPQKVWCASGKSKVIKGSLPLIGEADCLHEKYGGLNKSKPYYTGEHAKAIGNCP

IWVKTPLKLANGTKYRPPAKLLKERGFFGAIAGFLEGGWEGMIAGWHGYTSHGAHGVA

VAADLKSTQEAINKITKNLNSLSELEVKNLQRLSGAMDELHNEILELDEKVDDLRADT

ISSQIELAVLLSNEGIINSEDEHLLALERKLKKMLGPSAVEIGNGCFETKHKCNQTCL

DRIAAGTFDAGEFSLPTFDSLNITAASLNDDGLDNHTILLYYSTAASSLAVTLMIAIF

VVYMVSRDNVSCSICL"

ORIGIN

1 atgaaggcaa taattgtact actcatggta gtaacatcca atgcagatcg aatctgcact

61 gggataacat cgtcaaactc accacatgtc gtcaaaactg ctactcaagg ggaggtcaat

121 gtgaccggtg taataccact gacaacaaca cccaccaaat ctcattttgc aaatctcaaa

181 ggaacagaaa ccagggggaa actatgccca aaatgcctaa actgcacaga tctggatgta

241 gccttgggca gaccaaaatg cacagggaaa ataccctctg caagggtttc aatactccat

301 gaagtcagac ctgttacatc tgggtgcttt cctataatgc atgatagaac aaaaattaga

361 cagctgccta accttctccg aggatacgaa catgtcaggt tatcaactca aaacgttatc

421 aatacagaag atgcaccagg aggaccctac gaaattggaa cctcagggtc ttgcctcaac

481 attaccaatg gaaaaggatt cttcgcaaca atggcttggg ccgtcccaaa aaacaaaaca

541 gcaacaaatc cattaacaat agaagtacca tacatttgta cagaagaaga agaccaaatt

601 accgtttggg ggttccactc tgacgacgag acccaaatgg caaggctcta tggggattca

661 aagccccaga agttcacctc atctgccaac ggagtgacca cacattacgt ctcacagatt

721 ggtggcttcc caaatcaaac agaagacgga ggactaccac aaagtggcag aattgttgtt

781 gattacatgg tgcaaaaatc tggaaaaaca ggaacaatta cctatcaaag aggtatttta

841 ttgcctcaaa aggtgtggtg cgcaagtggc aagagcaagg taataaaagg atccttgccc

901 ttaattggag aagcagattg cctccatgaa aaatacggtg gattaaacaa aagcaagcct

961 tactacacag gggaacatgc aaaggccata ggaaattgcc caatatgggt gaaaacaccc

1021 ttgaagctgg ccaatggaac caaatataga cctcctgcaa aactattaaa ggaaagaggt

1081 ttcttcggag ccattgctgg tttcttagag ggaggatggg aaggaatgat tgcaggttgg

1141 cacggataca catcccatgg ggcacatgga gtagcagtgg cagctgacct taagagcact

1201 caagaggcca taaacaagat aacaaaaaat ctcaactctt tgagtgagct ggaagtaaag

1261 aatcttcaaa gactaagcgg tgccatggat gaactccaca acgaaatact agaactagat

1321 gagaaagtgg atgatctcag agctgataca ataagctcac aaatagaact cgcagtcctg

1381 ctttccaatg aaggaataat aaacagtgaa gatgaacatc tcttggcgct tgaaagaaag

1441 ctgaagaaaa tgctgggccc ctctgctgta gagataggga atggatgctt tgaaaccaaa

1501 cacaagtgca accagacctg tctcgacaga atagctgctg gtacctttga tgcaggagaa

1561 ttttctctcc ccacctttga ttcactgaat attactgctg catctttaaa tgacgatgga

1621 ttggacaatc atactatact gctttactac tcaactgctg cctccagttt ggctgtaaca

1681 ctgatgatag ctatctttgt tgtttatatg gtctccagag acaatgtttc ttgctccatt

1741 tgtctataa

//

LOCUS OR145886 1749 bp cRNA linear VRL 20-JUN-2023

DEFINITION Influenza B virus (B/Beijing/2327/2022) segment 4 hemagglutinin

(HA) gene, complete cds.

ACCESSION OR145886

VERSION OR145886

KEYWORDS .

SOURCE Influenza B virus

ORGANISM Influenza B virus

Viruses; Riboviria; Orthornavirae; Negarnaviricota;

Polyploviricotina; Insthoviricetes; Articulavirales;

Orthomyxoviridae; Betainfluenzavirus; Betainfluenzavirus

influenzae.

REFERENCE 1 (bases 1 to 1749)

AUTHORS Wang,Y.

TITLE Direct Submission

JOURNAL Submitted (20-JUN-2023) Department of Infectious Diseases, Peking

University People's Hospital, Xizhimen South Street, Beijing

100044, China

COMMENT ##Assembly-Data-START##

Sequencing Technology :: Sanger dideoxy sequencing

##Assembly-Data-END##

FEATURES Location/Qualifiers

source 1..1749

/organism="Influenza B virus"

/mol_type="viral cRNA"

/strain="B/Beijing/2327/2022"

/isolate="2327"

/isolation_source="nasal swab"

/host="Homo sapiens"

/db_xref="taxon:11520"

/segment="4"

/country="China: Beijing"

/collection_date="30-Aug-2022"

gene 1..1749

/gene="HA"

CDS 1..1749

/gene="HA"

/function="receptor binding and fusion protein"

/codon_start=1

/product="hemagglutinin"

/protein_id="WJE87857"

/translation="MKAIIVLLMVVTSNADRICTGITSSNSPHVVKTATQGEVNVTGV

IPLTTTPTKSHFANLKGTETRGKLCPKCLNCTDLDVALGRPKCTGKIPSARVSILHEV

RPVTSGCFPIMHDRTKIRQLPNLLRGYEHVRLSTQNVINTEDAPGGPYEIGTSGSCLN

ITNGKGFFATMAWAVPKNKTATNPLTIEVPYICTEEEDQITVWGFHSDDETQMARLYG

DSKPQKFTSSANGVTTHYVSQIGGFPNQTEDGGLPQSGRIVVDYMVQKSGKTGTITYQ

RGILLPQKVWCASGKSKVIKGSLPLIGEADCLHEKYGGLNKSKPYYTGEHAKAIGNCP

IWVKTPLKLANGTKYRPPAKLLKERGFFGAIAGFLEGGWEGMIAGWHGYTSHGAHGVA

VAADLKSTQEAINKITKNLNSLSELEVKNLQRLSGAMDELHNEILELDEKVDDLRADT

ISSQIELAVLLSNEGIINSEDEHLLALERKLKKMLGPSAVEIGNGCFETKHKCNQTCL

DRIAAGTFDAGEFSLPTFDSLNITAASLNDDGLDNHTILLYYSTAASSLAVTLMIAIF

VVYMVSRDNVSCSICL"

ORIGIN

1 atgaaggcaa taattgtact actcatggta gtaacatcca atgcagatcg aatctgcact

61 gggataacat cgtcaaactc accacatgtc gtcaaaactg ctactcaagg ggaggtcaat

121 gtgaccggtg taataccact gacaacaaca cccaccaaat ctcattttgc aaatctcaaa

181 ggaacagaaa ccagggggaa actatgccca aaatgcctaa actgcacaga tctggatgta

241 gccttgggca gaccaaaatg cacagggaaa ataccctctg caagggtttc aatactccat

301 gaagtcagac ctgttacatc tgggtgcttt cctataatgc atgatagaac aaaaattaga

361 cagctgccta accttctccg aggatacgaa catgtcaggt tatcaactca aaacgttatc

421 aatacagaag atgcaccagg aggaccctac gaaattggaa cctcagggtc ttgcctcaac

481 attaccaatg gaaaaggatt cttcgcaaca atggcttggg ccgtcccaaa aaacaaaaca

541 gcaacaaatc cattaacaat agaagtacca tacatttgta cagaagaaga agaccaaatt

601 accgtttggg ggttccactc tgacgacgag acccaaatgg caaggctcta tggggattca

661 aagccccaga agttcacctc atctgccaac ggagtgacca cacattacgt ctcacagatt

721 ggtggcttcc caaatcaaac agaagacgga ggactaccac aaagtggcag aattgttgtt

781 gattacatgg tgcaaaaatc tggaaaaaca ggaacaatta cctatcaaag aggtatttta

841 ttgcctcaaa aggtgtggtg cgcaagtggc aagagcaagg taataaaagg atccttgccc

901 ttaattggag aagcagattg cctccatgaa aaatacggtg gattaaacaa aagcaagcct

961 tactacacag gggaacatgc aaaggccata ggaaattgcc caatatgggt gaaaacaccc

1021 ttgaagctgg ccaatggaac caaatataga cctcctgcaa aactattaaa ggaaagaggt

1081 ttcttcggag ccattgctgg tttcttagag ggaggatggg aaggaatgat tgcaggttgg

1141 cacggataca catcccatgg ggcacatgga gtagcagtgg cagctgacct taagagcact

1201 caagaggcca taaacaagat aacaaaaaat ctcaactctt tgagtgagct ggaagtaaag

1261 aatcttcaaa gactaagcgg tgccatggat gaactccaca acgaaatact agaactagat

1321 gagaaagtgg atgatctcag agctgataca ataagctcac aaatagaact cgcagtcctg

1381 ctttccaatg aaggaataat aaacagtgaa gatgaacatc tcttggcgct tgaaagaaag

1441 ctgaagaaaa tgctgggccc ctctgctgta gagataggga atggatgctt tgaaaccaaa

1501 cacaagtgca accagacctg tctcgacaga atagctgctg gtacctttga tgcaggagaa

1561 ttttctctcc ccacttttga ttcactgaat attactgctg catctttaaa tgacgatgga

1621 ttggacaatc atactatact gctttactac tcaactgctg cctccagttt ggctgtaaca

1681 ctgatgatag ctatctttgt tgtttatatg gtctccagag acaatgtttc ttgctccatt

1741 tgtctataa

//

LOCUS OR145887 1749 bp cRNA linear VRL 20-JUN-2023

DEFINITION Influenza B virus (B/Beijing/2328/2022) segment 4 hemagglutinin

(HA) gene, complete cds.

ACCESSION OR145887

VERSION OR145887

KEYWORDS .

SOURCE Influenza B virus

ORGANISM Influenza B virus

Viruses; Riboviria; Orthornavirae; Negarnaviricota;

Polyploviricotina; Insthoviricetes; Articulavirales;

Orthomyxoviridae; Betainfluenzavirus; Betainfluenzavirus

influenzae.

REFERENCE 1 (bases 1 to 1749)

AUTHORS Wang,Y.

TITLE Direct Submission

JOURNAL Submitted (20-JUN-2023) Department of Infectious Diseases, Peking

University People's Hospital, Xizhimen South Street, Beijing

100044, China

COMMENT ##Assembly-Data-START##

Sequencing Technology :: Sanger dideoxy sequencing

##Assembly-Data-END##

FEATURES Location/Qualifiers

source 1..1749

/organism="Influenza B virus"

/mol_type="viral cRNA"

/strain="B/Beijing/2328/2022"

/isolate="2328"

/isolation_source="nasal swab"

/host="Homo sapiens"

/db_xref="taxon:11520"

/segment="4"

/country="China: Beijing"

/collection_date="30-Aug-2022"

gene 1..1749

/gene="HA"

CDS 1..1749

/gene="HA"

/function="receptor binding and fusion protein"

/codon_start=1

/product="hemagglutinin"

/protein_id="WJE87858"

/translation="MKAIIVLLMVVTSNADRICTGITSSNSPHVVKTATQGEVNVTGV

IPLTTTPTKSHFANLKGTETRGKLCPKCLNCTDLDVALGRPKCTGKIPSARVSILHEV

RPVTSGCFPIMHDRTKIRQLPNLLRGYEHVRLSTQNVINTEDAPGGPYEIGTSGSCLN

ITNGKGFFVTMAWAVPKNKTATNPLTIEVPYICTEEEDQITVWGFHSDDETQMARLYG

DSKPQKFTSSANGVTTHYVSQIGGFPNQTEDGGLPQSGRIVVDYMVQKSGKTGTITYQ

RGILLPQKVWCASGKSKVIKGSLPLIGEADCLHEKYGGLNKSKPYYTGEHAKAIGNCP

IWVKTPLKLANGTKYRPPAKLLKERGFFGAIAGFLEGGWEGMIAGWHGYTSHGAHGVA

VAADLKSTQEAINKITKNLNSLSELEVKNLQRLSGAMDELHNEILELDEKVDDLRADT

ISSQIELAVLLSNEGIINSEDEHLLALERKLKKMLGPSAVEIGNGCFETKHKCNQTCL

DRIAAGTFDAGEFSLPTFDSLNITAASLNDDGLDNHTILLYYSTAASSLAVTLMIAIF

VVYMVSRDNVSCSICL"

ORIGIN

1 atgaaggcaa taattgtact actcatggta gtaacatcca atgcagatcg aatctgcact

61 gggataacat cgtcaaactc accacatgtc gtcaaaactg ctactcaagg ggaggtcaat

121 gtgaccggtg taataccact gacaacaaca cccaccaaat ctcattttgc aaatctcaaa

181 ggaacagaaa ccagggggaa actatgccca aaatgcctaa actgcacaga tctggatgta

241 gccttgggca gaccaaaatg cacagggaaa ataccctctg caagggtttc aatactccat

301 gaagtcagac ctgttacatc tgggtgcttt cctataatgc atgatagaac aaaaattaga

361 cagctgccta accttctccg aggatacgaa catgtcaggt tatcaactca aaacgttatc

421 aatacagaag atgcaccagg aggaccctac gaaattggaa cctcagggtc ttgcctcaac

481 attaccaatg gaaaaggatt cttcgtaaca atggcttggg ccgtcccaaa aaacaaaaca

541 gcaacaaatc cattaacaat agaagtacca tacatttgta cagaagaaga agaccaaatt

601 accgtttggg ggttccactc tgacgacgag acccaaatgg caaggctcta tggggattca

661 aagccccaga agttcacctc atctgccaac ggagtgacca cacattacgt ctcacagatt

721 ggtggcttcc caaatcaaac agaagacgga ggactaccac aaagtggcag aattgttgtt

781 gattacatgg tgcaaaaatc tggaaaaaca ggaacaatta cctatcaaag aggtatttta

841 ttgcctcaaa aggtgtggtg cgcaagtggc aagagcaagg taataaaagg atccttgccc

901 ttaattggag aagcagattg cctccatgaa aaatacggtg gattaaacaa aagcaagcct

961 tactacacag gggaacatgc aaaggccata ggaaattgcc caatatgggt gaaaacaccc

1021 ttgaagctgg ccaatggaac caaatataga cctcctgcaa aattattaaa ggaaagaggt

1081 ttcttcggag ccattgctgg tttcttagag ggaggatggg aaggaatgat tgcaggttgg

1141 cacggataca catcccatgg ggcacatgga gtagcagtgg cagctgacct taagagcact

1201 caagaggcta taaacaagat aacaaaaaat ctcaactctt tgagtgagct ggaagtaaag

1261 aatcttcaaa gactaagcgg tgccatggat gaacttcaca acgaaatact agaactagat

1321 gagaaagtgg atgatctcag agctgataca ataagctcac aaatagaact cgcagtcctg

1381 ctttccaatg aaggaataat aaacagtgaa gatgaacatc tcttggcgct tgaaagaaag

1441 ctgaagaaaa tgctgggccc ctctgctgta gagataggga atggatgctt tgaaaccaaa

1501 cacaagtgca accagacctg tctcgacaga atagctgctg gtacctttga tgcaggagaa

1561 ttttctctcc ccacctttga ttcactgaat attactgctg catctttaaa tgacgatgga

1621 ttggacaatc atactatact gctttactac tcaactgctg cctccagttt ggctgtaaca

1681 ctgatgatag ctatctttgt tgtttatatg gtctccagag acaatgtttc ttgctccatt

1741 tgtctataa

//

LOCUS OR145888 1749 bp cRNA linear VRL 20-JUN-2023

DEFINITION Influenza B virus (B/Beijing/2329/2022) segment 4 hemagglutinin

(HA) gene, complete cds.

ACCESSION OR145888

VERSION OR145888

KEYWORDS .

SOURCE Influenza B virus

ORGANISM Influenza B virus

Viruses; Riboviria; Orthornavirae; Negarnaviricota;

Polyploviricotina; Insthoviricetes; Articulavirales;

Orthomyxoviridae; Betainfluenzavirus; Betainfluenzavirus

influenzae.

REFERENCE 1 (bases 1 to 1749)

AUTHORS Wang,Y.

TITLE Direct Submission

JOURNAL Submitted (20-JUN-2023) Department of Infectious Diseases, Peking

University People's Hospital, Xizhimen South Street, Beijing

100044, China

COMMENT ##Assembly-Data-START##

Sequencing Technology :: Sanger dideoxy sequencing

##Assembly-Data-END##

FEATURES Location/Qualifiers

source 1..1749

/organism="Influenza B virus"

/mol_type="viral cRNA"

/strain="B/Beijing/2329/2022"

/isolate="2329"

/isolation_source="nasal swab"

/host="Homo sapiens"

/db_xref="taxon:11520"

/segment="4"

/country="China: Beijing"

/collection_date="30-Aug-2022"

gene 1..1749

/gene="HA"

CDS 1..1749

/gene="HA"

/function="receptor binding and fusion protein"

/codon_start=1

/product="hemagglutinin"

/protein_id="WJE87859"

/translation="MKAIIVLLMVVTSNADRICTGITSSNSPHVVKTATQGEVNVTGV

IPLTTTPTKSHFANLKGTETRGKLCPKCLNCTDLDVALGRPKCTGKIPSARVSILHEV

RPVTSGCFPIMHDRTKIRQLPNLLRGYEHVRLSTQNVINTEDAPGGPYEIGTSGSCLN

ITNGKGFFATMAWAVPKNKTATNPLTIEVPYICTEEEDQITVWGFHSDDETQMARLYG

DSKPQKFTSSANGVTTHYVSQIGGFPNQTEDGGLPQSGRIVVDYMVQKSGKTGTITYQ

RGILLPQKVWCASGKSKVIKGSLPLIGEADCLHEKYGGLNKSKPYYTGEHAKAIGNCP

IWVKTPLKLANGTKYRPPAKLLKERGFFGAIAGFLEGGWEGMIAGWHGYTSHGAHGVA

VAADLKSTQEAINKITKNLNSLSELEVKNLQRLSGAMDELHNEILELDEKVDDLRADT

ISSQIELAVLLSNEGIINSEDEHLLALERKLKKMLGPSAVEIGNGCFETKHKCNQTCL

DRIAAGTFDAGEFSLPTFDSLNITAASLNDDGLDNHTILLYYSTAASSLAVTLMIAIF

VVYMVSRDNVSCSICL"

ORIGIN

1 atgaaggcaa taattgtact actcatggta gtaacatcca atgcagatcg aatctgcact

61 gggataacat cgtcaaactc accacatgtc gtcaaaactg ctactcaagg ggaggtcaat

121 gtgaccggtg taataccact gacaacaaca cccaccaaat ctcattttgc aaatctcaaa

181 ggaacagaaa ccagggggaa actatgccca aaatgcctaa actgcacaga tctggatgta

241 gccttgggca gaccaaaatg cacagggaaa ataccctctg caagggtttc aatactccat

301 gaagtcagac ctgttacatc tgggtgcttt cctataatgc atgatagaac aaaaattaga

361 cagctgccta accttctccg aggatacgaa catgtcaggt tatcaactca aaacgttatc

421 aatacagaag atgcaccagg aggaccctac gaaattggaa cctcagggtc ttgcctcaac

481 attaccaatg gaaaaggatt cttcgcaaca atggcctggg ccgtcccaaa aaacaaaaca

541 gcaacaaatc cattaacaat agaagtacca tacatttgta cagaagaaga agaccaaatt

601 accgtttggg ggttccactc tgacgacgag acccaaatgg caaggctcta tggggattca

661 aagccccaga agttcacctc atctgccaac ggagtgacca cacattacgt ctcacagatt

721 ggtggcttcc caaatcaaac agaagacgga ggactaccac aaagtggcag aattgttgtt

781 gattacatgg tgcaaaaatc tggaaaaaca ggaacaatta cctatcaaag aggtatttta

841 ttgcctcaaa aggtgtggtg cgcaagtggc aagagcaagg taataaaagg atccttgccc

901 ttaattggag aagcagattg ccttcatgaa aaatacggtg gattaaacaa aagcaagcct

961 tactacacag gggaacatgc aaaggccata ggaaattgcc caatatgggt gaaaacaccc

1021 ttgaagctgg ccaatggaac caaatataga cctcctgcaa aactattaaa ggaaagaggt

1081 ttcttcggag ccattgctgg tttcttagag ggaggatggg aaggaatgat tgcaggttgg

1141 cacggataca catcccatgg ggcacatgga gtagcagtgg cagctgacct taagagcact

1201 caagaggcca taaacaagat aacaaaaaat ctcaactctt tgagtgagct ggaagtaaag

1261 aatcttcaaa gactaagcgg tgccatggat gaactccaca acgaaatact agaactagat

1321 gagaaagtgg atgatctcag agctgataca ataagctcac aaatagaact cgcagtcctg

1381 ctttccaatg aaggaataat aaacagtgaa gatgaacatc tcttggcgct tgaaagaaag

1441 ctgaagaaaa tgctgggccc ctctgctgta gagataggga atggatgctt tgaaaccaaa

1501 cacaagtgca accagacctg tctcgacaga atagctgctg gtacctttga tgcaggagaa

1561 ttttctctcc ccacctttga ttcactgaat attactgctg catctttaaa tgacgatgga

1621 ttggacaatc atactatact gctttactac tcaactgctg cctccagttt ggctgtaaca

1681 ctgatgatag ctatctttgt tgtttatatg gtctccagag acaatgtttc ttgctccatt

1741 tgtctataa

//

LOCUS OR145889 1749 bp cRNA linear VRL 20-JUN-2023

DEFINITION Influenza B virus (B/Beijing/2330/2022) segment 4 hemagglutinin

(HA) gene, complete cds.

ACCESSION OR145889

VERSION OR145889

KEYWORDS .

SOURCE Influenza B virus

ORGANISM Influenza B virus

Viruses; Riboviria; Orthornavirae; Negarnaviricota;

Polyploviricotina; Insthoviricetes; Articulavirales;

Orthomyxoviridae; Betainfluenzavirus; Betainfluenzavirus

influenzae.

REFERENCE 1 (bases 1 to 1749)

AUTHORS Wang,Y.

TITLE Direct Submission

JOURNAL Submitted (20-JUN-2023) Department of Infectious Diseases, Peking

University People's Hospital, Xizhimen South Street, Beijing

100044, China

COMMENT ##Assembly-Data-START##

Sequencing Technology :: Sanger dideoxy sequencing

##Assembly-Data-END##

FEATURES Location/Qualifiers

source 1..1749

/organism="Influenza B virus"

/mol_type="viral cRNA"

/strain="B/Beijing/2330/2022"

/isolate="2330"

/isolation_source="nasal swab"

/host="Homo sapiens"

/db_xref="taxon:11520"

/segment="4"

/country="China: Beijing"

/collection_date="30-Aug-2022"

gene 1..1749

/gene="HA"

CDS 1..1749

/gene="HA"

/function="receptor binding and fusion protein"

/codon_start=1

/product="hemagglutinin"

/protein_id="WJE87860"

/translation="MKAIIVLLMVVTSNADRICTGITSSNSPHVVKTATQGEVNVTGV

IPLTTTPTKSHFANLKGTETRGKLCPKCLNCTDLDVALGRPKCTGKIPSARVSILHEV

RPVTSGCFPIMHDRTKIRQLPNLLRGYEHVRLSTQNVINTEDAPGGPYEIGTSGSCLN

ITNGKGFFVTMAWAVPKNKTATNPLTIEVPYICTEEEDQITVWGFHSDDETQMARLYG

DSKPQKFTSSANGVTTHYVSQIGGFPNQTEDGGLPQSGRIVVDYMVQKSGKTGTITYQ

RGILLPQKVWCASGKSKVIKGSLPLIGEADCLHEKYGGLNKSKPYYTGEHAKAIGNCP

IWVKTPLKLANGTKYRPPAKLLKERGFFGAIAGFLEGGWEGMIAGWHGYTSHGAHGVA

VAADLKSTQEAINKITKNLNSLSELEVKNLQRLSGAMDELHNEILELDEKVDDLRADT

ISSQIELAVLLSNEGIINSEDEHLLALERKLKKMLGPSAVEIGNGCFETKHKCNQTCL

DRIAAGTFDAGEFSLPTFDSLNITAASLNDDGLDNHTILLYYSTAASSLAVTLMIAIF

VVYMVSRDNVSCSICL"

ORIGIN

1 atgaaggcaa taattgtact actcatggta gtaacatcca atgcagatcg aatctgcact

61 gggataacat cgtcaaactc accacatgtc gtcaaaactg ctactcaagg ggaggtcaat

121 gtgaccggtg taataccact gacaacaaca cccaccaaat ctcattttgc aaatctcaaa

181 ggaacagaaa ccagggggaa actatgccca aaatgcctaa actgcacaga tctggatgta

241 gccttgggca gaccaaaatg cacagggaaa ataccctctg caagggtttc aatactccat

301 gaagtcagac ctgttacatc tgggtgcttt cctataatgc atgatagaac aaaaattaga

361 cagctgccta accttctccg aggatacgaa catgtcaggt tatcaactca aaacgttatc

421 aatacagaag atgcaccagg aggaccctac gaaattggaa cctcagggtc ttgcctcaac

481 attaccaatg gaaaaggatt cttcgtaaca atggcttggg ccgtcccaaa aaacaaaaca

541 gcaacaaatc cattaacaat agaagtacca tacatttgta cagaagaaga agaccaaatt

601 accgtttggg ggttccactc tgacgacgag acccaaatgg caaggctcta tggggattca

661 aagccccaga agttcacctc atctgccaac ggagtgacca cacattacgt ctcacagatt

721 ggtggctttc caaatcaaac agaagacgga ggactaccac aaagtggcag aattgttgtt

781 gattacatgg tgcaaaaatc tggaaaaaca ggaacaatta cctatcaaag aggtatttta

841 ttgcctcaaa aggtgtggtg cgcaagtggc aagagcaagg taataaaagg atccttgccc

901 ttaattggag aagcagattg cctccatgaa aaatacggtg gattaaacaa aagcaagcct

961 tactacacag gggaacatgc aaaggccata ggaaattgcc caatatgggt gaaaacaccc

1021 ttgaagctgg ccaatggaac caaatataga cctcctgcaa aactattaaa ggaaagaggt

1081 ttcttcggag ccattgctgg tttcttagag ggaggatggg aaggaatgat tgcaggttgg

1141 cacggataca catcccatgg ggcacatgga gtagcagtgg cagctgacct taagagcact

1201 caagaggcca taaacaagat aacaaaaaat ctcaactctt tgagtgagct ggaagtaaag

1261 aatcttcaaa gactaagcgg tgccatggat gaactccaca acgaaatact agaactagat

1321 gagaaagtgg atgatctcag agctgataca ataagctcac aaatagaact cgcagtcctg

1381 ctttccaatg aaggaataat aaacagtgaa gatgaacatc tcttggcgct tgaaagaaag

1441 ctgaagaaaa tgctgggccc ctctgctgta gagataggga atggatgctt tgaaaccaaa

1501 cacaagtgca accagacctg tctcgacaga atagctgctg gtacctttga tgcaggagaa

1561 ttttctctcc ccacctttga ttcactgaat attactgctg catctttaaa tgacgatgga

1621 ttggacaatc atactatact gctttactac tcaactgctg cctccagttt ggctgtaaca

1681 ctgatgatag ctatctttgt tgtttatatg gtctccagag acaatgtttc ttgctccatt

1741 tgtctataa

//

LOCUS OR145890 1749 bp cRNA linear VRL 20-JUN-2023

DEFINITION Influenza B virus (B/Beijing/2331/2022) segment 4 hemagglutinin

(HA) gene, complete cds.

ACCESSION OR145890

VERSION OR145890

KEYWORDS .

SOURCE Influenza B virus

ORGANISM Influenza B virus

Viruses; Riboviria; Orthornavirae; Negarnaviricota;

Polyploviricotina; Insthoviricetes; Articulavirales;

Orthomyxoviridae; Betainfluenzavirus; Betainfluenzavirus

influenzae.

REFERENCE 1 (bases 1 to 1749)

AUTHORS Wang,Y.

TITLE Direct Submission

JOURNAL Submitted (20-JUN-2023) Department of Infectious Diseases, Peking

University People's Hospital, Xizhimen South Street, Beijing

100044, China

COMMENT ##Assembly-Data-START##

Sequencing Technology :: Sanger dideoxy sequencing

##Assembly-Data-END##

FEATURES Location/Qualifiers

source 1..1749

/organism="Influenza B virus"

/mol_type="viral cRNA"

/strain="B/Beijing/2331/2022"

/isolate="2331"

/isolation_source="nasal swab"

/host="Homo sapiens"

/db_xref="taxon:11520"

/segment="4"

/country="China: Beijing"

/collection_date="30-Aug-2022"

gene 1..1749

/gene="HA"

CDS 1..1749

/gene="HA"

/function="receptor binding and fusion protein"

/codon_start=1

/product="hemagglutinin"

/protein_id="WJE87861"

/translation="MKAIIVLLMVVTSNADRICTGITSSNSPHVVKTATQGEVNVTGV

IPLTTTPTKSHFANLKGTETRGKLCPKCLNCTDLDVALGRPKCTGKIPSARVSILHEV

RPVTSGCFPIMHDRTKIRQLPNLLRGYEHVRLSTQNVINTGDAPGGPYEIGTSGSCLN

ITNGKGFFVTMAWAVPKNKTATNPLTIEVPYICTEEEDQITVWGFHSDDETQMARLYG

DSKPQKFTSSANGVTTHYVSQIGGFPNQTEDGGLPQSGRIVVDYMVQKSGKTGTITYQ

RGILLPQKVWCASGKSKVIKGSLPLIGEADCLHEKYGGLNKSKPYYTGEHAKAIGNCP

IWVKTPLKLANGTKYRPPAKLLKERGFFGAIAGFLEGGWEGMIAGWHGYTSHGAHGVA

VAADLKSTQEAINKITKNLNSLSELEVKNLQRLSGAMDELHNEILELDEKVDDLRADT

ISSQIELAVLLSNEGIINSEDEHLLALERKLKKMLGPSAVEIGNGCFETKHKCNQTCL

DRIAAGTFDAGEFSLPTFDSLNITAASLNDDGLDNHTILLYYSTAASSLAVTLMIAIF

VVYMVSRDNVSCSICL"

ORIGIN

1 atgaaggcaa taattgtact actcatggta gtaacatcca atgcagatcg aatctgcact

61 gggataacat cgtcaaactc accacatgtc gtcaaaactg ctactcaagg ggaggtcaat

121 gtgaccggtg taataccact gacaacaaca cccaccaaat ctcattttgc aaatctcaaa

181 ggaacagaaa ccagggggaa actatgccca aaatgcctaa actgcacaga tctggatgta

241 gccttgggca gaccaaaatg cacagggaaa ataccctctg caagggtttc aatactccat

301 gaagtcagac ctgttacatc tgggtgcttt cctataatgc atgatagaac aaaaattaga

361 cagctgccta accttctccg aggatacgaa catgtcaggt tatcaactca aaacgttatc

421 aatacaggag atgcaccagg aggaccctac gaaattggaa cctcagggtc ttgcctcaac

481 attaccaatg gaaaaggatt cttcgtaaca atggcttggg ccgtcccaaa aaacaaaaca

541 gcaacaaatc cattaacaat agaagtacca tacatttgta cagaagaaga agaccaaatt

601 accgtttggg ggttccactc tgacgacgag acccaaatgg caaggctcta tggggattca

661 aagccccaga agttcacctc atctgccaac ggagtgacca cacattacgt ctcacagatt

721 ggtggcttcc caaatcaaac agaagacgga ggactaccac aaagtggcag aattgttgtt

781 gattacatgg tgcaaaaatc tggaaaaaca ggaacaatta cctatcaaag aggtatttta

841 ttgcctcaaa aggtgtggtg cgcaagtggc aagagcaagg taataaaagg atccttgccc

901 ttaattggag aagcagattg cctccatgaa aaatacggtg gattaaacaa aagcaagcct

961 tactacacag gggaacatgc aaaggccata ggaaattgcc caatatgggt gaaaacaccc

1021 ttgaagctgg ccaatggaac caaatataga cctcctgcaa aattattaaa ggaaagaggt

1081 ttcttcggag ccattgctgg tttcttagag ggaggatggg aaggaatgat tgcaggttgg

1141 cacggataca catcccatgg ggcacatgga gtagcagtgg cagctgacct taagagcact

1201 caagaggcta taaacaagat aacaaaaaat ctcaactctt tgagtgagct ggaagtaaag

1261 aatcttcaaa gactaagcgg tgcaatggat gaactccata acgaaatact agaactagat

1321 gagaaagtgg atgatctcag agctgataca ataagctcac aaatagaact cgcagtcctg

1381 ctttccaatg aaggaataat aaacagtgaa gatgaacatc tcttggcgct tgaaagaaag

1441 ctgaagaaaa tgctgggccc ctctgctgta gagataggga atggatgctt tgaaaccaaa

1501 cacaagtgca accagacctg tctcgacaga atagctgctg gtacctttga tgcaggagaa

1561 ttttctctcc ccacctttga ttcactgaat attactgctg catctttaaa tgacgatgga

1621 ttggacaatc atactatact gctttactac tcaactgctg cctccagttt ggctgtaaca

1681 ctgatgatag ctatctttgt tgtttatatg gtctccagag acaatgtttc ttgctccatt

1741 tgtctataa

//

LOCUS OR145891 1749 bp cRNA linear VRL 20-JUN-2023

DEFINITION Influenza B virus (B/Beijing/2332/2022) segment 4 hemagglutinin

(HA) gene, complete cds.

ACCESSION OR145891

VERSION OR145891

KEYWORDS .

SOURCE Influenza B virus

ORGANISM Influenza B virus

Viruses; Riboviria; Orthornavirae; Negarnaviricota;

Polyploviricotina; Insthoviricetes; Articulavirales;

Orthomyxoviridae; Betainfluenzavirus; Betainfluenzavirus

influenzae.

REFERENCE 1 (bases 1 to 1749)

AUTHORS Wang,Y.

TITLE Direct Submission

JOURNAL Submitted (20-JUN-2023) Department of Infectious Diseases, Peking

University People's Hospital, Xizhimen South Street, Beijing

100044, China

COMMENT ##Assembly-Data-START##

Sequencing Technology :: Sanger dideoxy sequencing

##Assembly-Data-END##

FEATURES Location/Qualifiers

source 1..1749

/organism="Influenza B virus"

/mol_type="viral cRNA"

/strain="B/Beijing/2332/2022"

/isolate="2332"

/isolation_source="nasal swab"

/host="Homo sapiens"

/db_xref="taxon:11520"

/segment="4"

/country="China: Beijing"

/collection_date="30-Aug-2022"

gene 1..1749

/gene="HA"

CDS 1..1749

/gene="HA"

/function="receptor binding and fusion protein"

/codon_start=1

/product="hemagglutinin"

/protein_id="WJE87862"

/translation="MKAIIVLLMVVTSNADRICTGITSSNSPHVVKTATQGEVNVTGV

IPLTTTPTKSHFANLKGTETRGKLCPKCLNCTDLDVALGRPKCTGKIPSAKVSILHEV

RPVTSGCFPMMHDRTKIRQLPNLLRGYEHVRLSTHNVINAEDAPGGPYEIGTSGSCPN

ITNGKGFFATMAWAVPKNKTATNPLTIEVPYICTEEEDQITVWGFHSDDETQMAKLYG

DSKPQKFTSSANGMTTHYVSQIGGFPNQTEDGGLQQSGRIVVDYMVQKSGKTGTITYQ

RGILLPQKVWCASGKSKVIKGSLPLVGEADCLHEKYGGLNKSKPYYTGEHAKAIGNCP

IWVKTPLKLANGTKYRPPAKLLKERGFFGAIAGFLEGGWEGMIAGWHGYTSHGAHGVA

VAADLKSTQEAINKITKNLNSLSELEVKNLQRLSGAMDELQNEILELDEKVDDLRADT

ISSQIELAVLLSNEGIINSEDEHLLALERKLKKMLGPSAVEIGNGCFETKHKCNQTCL

DRIAAGTFDAGEFSLPTFDSLNITAASLNDDGLDNHTILLYYSTAASSLAVTLMIAIF

VVYMVSRDNVSCSICL"

ORIGIN

1 atgaaggcaa taattgtact actcatggta gtaacatcca atgcagatcg aatctgcact

61 gggataacat cgtcaaactc accacatgtc gtcaaaactg ctactcaagg ggaggtcaat

121 gtgaccggtg taataccact gacaacaaca cccaccaaat ctcattttgc aaatctcaaa

181 ggaacagaaa ccagggggaa actatgccca aaatgcctca actgcacaga tctggatgta

241 gccttgggca gaccaaaatg cacagggaaa ataccctctg caaaggtttc aatactccat

301 gaagtcagac ctgttacatc tgggtgcttt cccatgatgc atgatagaac aaaaattaga

361 cagctgccta accttctccg aggatacgaa catgtcagat tatcaactca caacgttatc

421 aatgcagaag atgcaccagg aggaccctac gaaattggaa cctcagggtc ttgccctaac

481 attaccaatg gaaaaggatt cttcgcaaca atggcttggg ccgtcccaaa aaacaaaaca

541 gcaacaaatc cattaacaat agaagtacca tacatttgta cagaagaaga agaccaaatt

601 accgtttggg ggttccactc tgacgacgag acccaaatgg caaagctcta tggggactca

661 aagccccaga agttcacctc atctgccaac ggaatgacca cacattacgt ttcacagatt

721 ggtggcttcc caaatcaaac agaagacgga ggactacaac aaagtggcag aattgttgtt

781 gattacatgg tgcaaaaatc tggaaaaaca ggaacaatta cctatcaaag aggtatttta

841 ttgccccaaa aggtgtggtg cgcaagtggc aagagcaagg taataaaagg atccttgccc

901 ttagttggag aagcagattg cctccatgaa aaatacggtg gattaaacaa aagcaagcct

961 tactacacag gggaacatgc aaaggccata ggaaattgcc caatatgggt gaaaacaccc

1021 ttgaagctgg ccaatggaac caaatataga cctcctgcaa aactattaaa ggaaagaggt

1081 ttcttcggag ccattgctgg tttcttagag ggaggatggg aaggaatgat tgcaggttgg

1141 cacggataca catcccatgg ggcacatgga gtagcggtgg cagctgacct taagagcact

1201 caagaggcca taaacaagat aacaaaaaat ctcaactctt tgagtgagct ggaagtaaag

1261 aatcttcaaa gactaagcgg tgccatggat gaactccaaa acgaaatact agaactagat

1321 gagaaagtgg atgatctcag agctgataca ataagctcac aaatagaact cgcagtcctg

1381 ctttccaatg aaggaataat aaacagtgaa gatgaacatc tcttggcgct tgaaagaaag

1441 ctgaagaaaa tgctgggccc ctctgctgta gagataggga atggatgctt tgagaccaaa

1501 cacaagtgca accagacctg tctcgacaga atagctgctg gtacctttga tgcaggagaa

1561 ttttctctcc ccacttttga ttcactgaat attactgctg catctttaaa tgacgatgga

1621 ttggacaatc atactatact gctttactac tcaactgctg cctccagttt ggctgtaaca

1681 ctgatgatag ctatctttgt tgtttatatg gtctccagag acaatgtttc ttgctccatt

1741 tgtctataa

//

LOCUS OR145892 1749 bp cRNA linear VRL 20-JUN-2023

DEFINITION Influenza B virus (B/Beijing/2333/2022) segment 4 hemagglutinin

(HA) gene, complete cds.

ACCESSION OR145892

VERSION OR145892

KEYWORDS .

SOURCE Influenza B virus

ORGANISM Influenza B virus

Viruses; Riboviria; Orthornavirae; Negarnaviricota;

Polyploviricotina; Insthoviricetes; Articulavirales;

Orthomyxoviridae; Betainfluenzavirus; Betainfluenzavirus

influenzae.

REFERENCE 1 (bases 1 to 1749)

AUTHORS Wang,Y.

TITLE Direct Submission

JOURNAL Submitted (20-JUN-2023) Department of Infectious Diseases, Peking

University People's Hospital, Xizhimen South Street, Beijing

100044, China

COMMENT ##Assembly-Data-START##

Sequencing Technology :: Sanger dideoxy sequencing

##Assembly-Data-END##

FEATURES Location/Qualifiers

source 1..1749

/organism="Influenza B virus"

/mol_type="viral cRNA"

/strain="B/Beijing/2333/2022"

/isolate="2333"

/isolation_source="nasal swab"

/host="Homo sapiens"

/db_xref="taxon:11520"

/segment="4"

/country="China: Beijing"

/collection_date="30-Aug-2022"

gene 1..1749

/gene="HA"

CDS 1..1749

/gene="HA"

/function="receptor binding and fusion protein"

/codon_start=1

/product="hemagglutinin"

/protein_id="WJE87863"

/translation="MKAIIVLLMVVTSNADRICTGITSSNSPHVVKTATQGEVNVTGV

IPLTTTPTKSHFANLKGTETRGNLCPKCLNCTDLDVALGRPKCTGKIPSARVSILHEV

RPVTSGCFPIMHDRTKIRQLPNLLRGYEHVRLSTQNVINTEDAPGGPYEIGTSGSCLN

ITNGKGFFATMAWAVPKNKTATNPLTIEVPYICTEEEDQITVWGFHSDDETQMAKLYG

DSKPQKFTSSANGVTTHYVSQIGGFPNQTEDGGLPQSGRIVVDYMVQKSGKTGTITYQ

RGILLPQKVWCASGKSKVIKGSLPLIGEADCLHEKYGGLNKSKPYYTGEHAKAIGNCP

IWVKTPLKLANGTKYRPPAKLLKERGFFGAIAGFLEGGWEGMIAGWHGYTSHGAHGVA

VAADLKSTQEAINKITKNLNSLSELEVKNLQRLSGAMDELHNEILELDEKVDDLRADT

ISSQIELAVLLSNEGIINSEDEHLLALERKLKKMLGPSAVEIGNGCFETKHKCNQTCL

DRIAAGTFDAGEFSLPTFDSLNITAASLNDDGLDNNTILLYYSTAASSLAVTLMIAIF

VVYMVSRDNVSCSICL"

ORIGIN

1 atgaaggcaa taattgtact actcatggta gtaacatcca atgcagatcg aatctgcact

61 gggataacat cgtcaaactc accacatgtc gtcaaaactg ctactcaagg ggaggtcaat

121 gtgaccggtg taataccact gacaacaaca cccaccaaat ctcattttgc aaatcttaaa

181 ggaacagaaa ccagggggaa cctatgccca aaatgtctaa actgcacaga tctggatgta

241 gccttgggca gaccaaaatg cacagggaaa ataccctctg caagggtttc aatactccat

301 gaagtcagac ctgttacatc tgggtgcttt cctataatgc atgatagaac aaaaattaga

361 cagctgccta accttctccg aggatacgaa catgtcaggt tatcaactca aaacgttatc

421 aatacagaag atgcaccagg aggaccctac gaaattggaa cctcagggtc ttgcctcaac

481 attaccaatg gaaaaggatt cttcgcaaca atggcttggg ccgtcccaaa aaacaaaaca

541 gcaacaaatc cattaacaat agaagtacca tacatttgta cagaagaaga agaccaaatt

601 accgtttggg ggttccactc tgacgacgag acccaaatgg ccaagctcta tggggattca

661 aagccccaga agttcacctc atctgccaac ggagtgacca cacattacgt ctcacagatt

721 ggtggcttcc caaatcaaac agaagacgga ggactaccac aaagtggcag aattgttgtt

781 gattacatgg tgcaaaaatc tggaaaaaca ggaacaatta cctatcaaag aggtatttta

841 ttgcctcaaa aggtgtggtg cgcaagtggc aagagcaagg taataaaagg atccttgccc

901 ttaattggag aagcagattg cctccatgaa aaatacggtg gattaaacaa aagcaagcct

961 tactacacag gggaacatgc aaaggccata ggaaattgcc caatatgggt gaaaacaccc

1021 ttgaagctgg ccaatggaac caaatataga cctcctgcaa aactattaaa ggaaagaggt

1081 ttcttcggag ccattgctgg tttcttagag ggaggatggg aaggaatgat tgcaggttgg

1141 cacggataca catcccatgg ggcacatgga gtagcagtgg cagctgacct taagagcact

1201 caagaggcca taaacaagat aacaaaaaat ctcaactctt tgagtgagct ggaagtaaag

1261 aatcttcaaa gactaagcgg tgccatggat gaactccaca acgaaatact agaactagat

1321 gagaaagtgg atgatctcag agctgataca ataagctcac aaatagaact cgcagtcctg

1381 ctttccaatg aaggaataat aaacagtgag gatgaacatc tcttggcgct tgaaagaaag

1441 ctgaagaaaa tgctgggccc ctctgctgta gagataggga atggatgctt tgaaaccaaa

1501 cacaagtgca accagacctg tctcgacaga atagctgctg gtacctttga tgcaggagaa

1561 ttttctctcc ccacctttga ttcactgaat attactgctg catctttaaa tgacgatgga

1621 ttggacaata atactatact gctttactac tcaactgctg cctccagttt ggctgtaaca

1681 ctgatgatag ctatctttgt tgtttatatg gtctccagag acaatgtttc ttgctccatt

1741 tgtctataa

//

LOCUS OR145893 1749 bp cRNA linear VRL 20-JUN-2023

DEFINITION Influenza B virus (B/Beijing/2334/2022) segment 4 hemagglutinin

(HA) gene, complete cds.

ACCESSION OR145893

VERSION OR145893

KEYWORDS .

SOURCE Influenza B virus

ORGANISM Influenza B virus

Viruses; Riboviria; Orthornavirae; Negarnaviricota;

Polyploviricotina; Insthoviricetes; Articulavirales;

Orthomyxoviridae; Betainfluenzavirus; Betainfluenzavirus

influenzae.

REFERENCE 1 (bases 1 to 1749)

AUTHORS Wang,Y.

TITLE Direct Submission

JOURNAL Submitted (20-JUN-2023) Department of Infectious Diseases, Peking

University People's Hospital, Xizhimen South Street, Beijing

100044, China

COMMENT ##Assembly-Data-START##

Sequencing Technology :: Sanger dideoxy sequencing

##Assembly-Data-END##

FEATURES Location/Qualifiers

source 1..1749

/organism="Influenza B virus"

/mol_type="viral cRNA"

/strain="B/Beijing/2334/2022"

/isolate="2334"

/isolation_source="nasal swab"

/host="Homo sapiens"

/db_xref="taxon:11520"

/segment="4"

/country="China: Beijing"

/collection_date="30-Aug-2022"

gene 1..1749

/gene="HA"

CDS 1..1749

/gene="HA"

/function="receptor binding and fusion protein"

/codon_start=1

/product="hemagglutinin"

/protein_id="WJE87864"

/translation="MKAIIVLLMVVTSNADRICTGITSSNSPHVVKTATQGEVNVTGV

IPLTTTPTKSHFANLKGTETRGKLCPKCLNCTDLDVALGRPKCTGKIPSARVSILHEV

RPVTSGCFPIMHDRTKIRQLPNLLRGYEHVRLSTQNVINTEDAPGGPYEIGTSGSCLN

ITNGKGFFATMAWAVPKNKTATNPLTIEVPYICTEEEDQITVWGFHSDDETQMARLYG

DSKPQKFTSSANGVTTHYVSQIGGFPNQTEDGGLPQSGRIVVDYMVQKSGKTGTITYQ

RGILLPQKVWCASGKSKVIKGSLPLIGEADCLHEKYGGLNKSKPYYTGEHAKAIGNCP

IWVKTPLKLANGTKYRPPARLLKERGFFGAIAGFLEGGWEGMIAGWHGYTSHGAHGVA

VAADLKSTQEAINKITKNLNSLSELEVKNLQRLSGAMDELHNEILELDEKVDDLRADT

ISSQIELAVLLSNEGIINSEDEHLLALERKLKKMLGPSAVEIGNGCFETKHKCNQTCL

DRIAAGTFDAGEFSLPTFDSLNITAASLNDDGLDNHTILLYYSTAASSLAVTLMIAIF

VVYMVSRDNVSCSICL"

ORIGIN

1 atgaaggcaa taattgtact actcatggta gtaacatcca atgcagatcg aatctgcact

61 gggataacat cgtcaaactc accacatgtc gtcaaaactg ctactcaagg ggaggtcaat

121 gtgaccggtg taataccact gacaacaaca cccaccaaat ctcattttgc aaatctcaaa

181 ggaacagaaa ccagggggaa actatgccca aaatgcctaa actgcacaga tctggatgta

241 gccttgggca gaccaaaatg cacagggaaa ataccctctg caagggtttc aatactccat

301 gaagtcagac ctgttacatc tgggtgcttt cctataatgc atgatagaac aaaaattaga

361 cagctgccta accttctccg aggatacgaa catgtcaggt tatcaactca aaacgttatc

421 aatacagaag atgcaccagg aggaccctac gaaattggaa cctcagggtc ttgcctcaac

481 attaccaatg gaaaaggatt cttcgcaaca atggcttggg ccgtcccaaa aaacaaaaca

541 gcaacaaatc cattaacaat agaagtacca tacatttgta cagaagaaga agaccaaatt

601 accgtttggg ggttccactc tgacgacgag acccaaatgg caaggctcta tggggattca

661 aagccccaga agttcacctc atctgccaac ggagtgacca cacattacgt ctcacagatt

721 ggtggcttcc caaatcaaac agaagacgga ggactaccac aaagtggcag aattgttgtt

781 gattacatgg tgcaaaaatc tggaaaaaca ggaacaatta cctatcaaag aggtatttta

841 ttgcctcaaa aggtgtggtg cgcaagtggc aagagcaaag taataaaagg atccttgccc

901 ttaattggag aagcagattg cctccatgaa aaatacggtg gattaaacaa aagcaagcct

961 tactacacag gggaacatgc aaaggccata ggaaattgcc caatatgggt gaaaacaccc

1021 ttgaagctgg ccaatggaac caaatataga cctcctgcaa gactattaaa ggaaagaggt

1081 ttcttcggag ccattgctgg tttcttagag ggaggatggg aaggaatgat tgcaggttgg

1141 cacggataca catcccatgg ggcacatgga gtagcagtgg cagctgacct taagagcact

1201 caagaggcca taaacaagat aacaaaaaat ctcaactctt tgagtgagct ggaagtaaag

1261 aatcttcaaa gactaagcgg tgccatggat gaactccaca acgaaatact agaactagat

1321 gagaaagtgg atgatctcag agctgataca ataagctcac aaatagaact cgcagtcctg

1381 ctttccaatg aaggaataat aaacagtgaa gatgaacatc tcttggcgct tgaaagaaag

1441 ctgaagaaaa tgctgggccc ctctgctgta gagataggga atggatgctt tgaaaccaaa

1501 cacaagtgca accagacctg tctcgacaga atagctgctg gtacctttga tgcaggagaa

1561 ttttctctcc ccacctttga ttcactgaat attactgctg catctttaaa tgacgatgga

1621 ttggacaatc atactatact gctttactac tcaactgctg cctccagttt ggctgtaaca

1681 ctgatgatag ctatctttgt tgtttatatg gtctccagag acaatgtttc ttgctccatt

1741 tgtctataa

//

LOCUS OR145894 1749 bp cRNA linear VRL 20-JUN-2023

DEFINITION Influenza B virus (B/Beijing/2335/2022) segment 4 hemagglutinin

(HA) gene, complete cds.

ACCESSION OR145894

VERSION OR145894

KEYWORDS .

SOURCE Influenza B virus

ORGANISM Influenza B virus

Viruses; Riboviria; Orthornavirae; Negarnaviricota;

Polyploviricotina; Insthoviricetes; Articulavirales;

Orthomyxoviridae; Betainfluenzavirus; Betainfluenzavirus

influenzae.

REFERENCE 1 (bases 1 to 1749)

AUTHORS Wang,Y.

TITLE Direct Submission

JOURNAL Submitted (20-JUN-2023) Department of Infectious Diseases, Peking

University People's Hospital, Xizhimen South Street, Beijing

100044, China

COMMENT ##Assembly-Data-START##

Sequencing Technology :: Sanger dideoxy sequencing

##Assembly-Data-END##

FEATURES Location/Qualifiers

source 1..1749

/organism="Influenza B virus"

/mol_type="viral cRNA"

/strain="B/Beijing/2335/2022"

/isolate="2335"

/isolation_source="nasal swab"

/host="Homo sapiens"

/db_xref="taxon:11520"

/segment="4"

/country="China: Beijing"

/collection_date="30-Aug-2022"

gene 1..1749

/gene="HA"

CDS 1..1749

/gene="HA"

/function="receptor binding and fusion protein"

/codon_start=1

/product="hemagglutinin"

/protein_id="WJE87865"

/translation="MKAIIVLLMVVTSNADRICTGITSSNSPHVVKTATQGEVNVTGV

IPLTTTPTKSHFANLKGTETRGKLCPKCLNCTDLDVALGRPKCTGKIPSARVSILHEV

RPVTSGCFPIMHDRTKIRQLPNLLRGYEHVRLSTQNVINTEDAPGGPYEIGTSGSCLN

ITNGKGFFVTMAWAVPKNKTATNPLTIEVPYICTEEEDQITVWGFHSDDETQMARLYG

DSKPQKFTSSANGVTTHYVSQIGGFPNQTEDGGLPQSGRIVVDYMVQKSGKTGTITYQ

RGILLPQKVWCASGKSKVIKGSLPLIGEADCLHEKYGGLNKSKPYYTGEHAKAIGNCP

IWVKTPLKLANGTKYRPPAKLLKERGFFGAIAGFLEGGWEGMIAGWHGYTSHGAHGVA

VAADLKSTQEAINKITKNLNSLSELEVKNLQRLGSAMDELHNEILELDEKVDDLRADT

ISSQIELAVLLSNEGIINSEDEHLLALERKLKKMLGPSAVEIGNGCFETKHKCNQTCL

DRIAAGTFDAGEFSLPTFDSLNITAASLNDDGLDNHTILLYYSTAASSLAVTLMIAIF

VVYMVSRDNVSCSICL"

ORIGIN

1 atgaaggcaa taattgtact actcatggta gtaacatcca atgcagatcg aatctgcact

61 gggataacat cgtcaaactc accacatgtc gtcaaaactg ctactcaagg ggaggtcaat

121 gtgaccggtg taataccact gacaacaaca cccaccaaat ctcattttgc aaatctcaaa

181 ggaacagaaa ccagggggaa actatgccca aaatgcctaa actgcacaga tctggatgta

241 gccttgggca gaccaaaatg cacagggaaa ataccctctg caagggtttc aatactccat

301 gaagtcagac ctgttacatc tgggtgcttt cctataatgc atgatagaac aaaaattaga

361 cagctgccta accttctccg aggatacgaa catgtcaggt tatcaactca aaacgttatc

421 aatacagaag atgcaccagg aggaccctac gaaattggaa cctcagggtc ttgcctcaac

481 attaccaatg gaaaaggatt cttcgtaaca atggcttggg ccgtcccaaa aaacaaaaca

541 gcaacaaatc cattaacaat agaagtacca tacatttgta cagaagaaga agaccaaatt

601 accgtttggg ggttccactc tgacgacgag acccaaatgg caaggctcta tggggattca

661 aagccccaga agttcacctc atctgccaac ggagtgacca cacattacgt ctcacagatt

721 ggtggcttcc caaatcaaac agaagacgga ggactaccac aaagtggcag aattgttgtt

781 gattacatgg tgcaaaaatc tggaaaaaca ggaacaatta cctatcaaag aggtatttta

841 ttgcctcaaa aggtgtggtg cgcaagtggc aagagcaagg taataaaagg atccttgccc

901 ttaattggag aagcagattg cctccatgaa aaatacggtg gattaaacaa aagcaagcct

961 tactacacag gggaacatgc aaaggccata ggaaattgcc caatatgggt gaaaacaccc

1021 ttgaagctgg ccaatggaac caaatataga cctcctgcaa aattattaaa ggaaagaggt

1081 ttcttcggag ccattgctgg tttcttagag ggaggatggg aaggaatgat tgcaggttgg

1141 cacggataca catcccatgg ggcacatgga gtagcagtgg cagctgacct taagagcact

1201 caagaggcta taaacaagat aacaaaaaat ctcaactctt tgagtgagct ggaagtaaag

1261 aatcttcaaa gactaggcag tgccatggat gaactccaca acgaaatact agaactagat

1321 gagaaagtgg atgatctcag agctgataca ataagctcac aaatagaact cgcagtcctg

1381 ctttccaatg aaggaataat aaacagtgaa gatgaacatc tcttggcgct tgaaagaaag

1441 ctgaagaaaa tgctgggccc ctctgctgta gagataggga atggatgctt tgaaaccaaa

1501 cacaagtgca accagacctg tctcgacaga atagctgctg gtacctttga tgcaggagaa

1561 ttttctctcc ccacctttga ttcactgaat attactgctg catctttaaa tgacgatgga

1621 ttggacaatc atactatact gctttactac tcaactgctg cctccagttt ggctgtaaca

1681 ctgatgatag ctatctttgt tgtttatatg gtctccagag acaatgtttc ttgctccatt

1741 tgtctataa

//

LOCUS OR145895 1749 bp cRNA linear VRL 20-JUN-2023

DEFINITION Influenza B virus (B/Beijing/2336/2022) segment 4 hemagglutinin

(HA) gene, complete cds.

ACCESSION OR145895

VERSION OR145895

KEYWORDS .

SOURCE Influenza B virus

ORGANISM Influenza B virus

Viruses; Riboviria; Orthornavirae; Negarnaviricota;

Polyploviricotina; Insthoviricetes; Articulavirales;

Orthomyxoviridae; Betainfluenzavirus; Betainfluenzavirus

influenzae.

REFERENCE 1 (bases 1 to 1749)

AUTHORS Wang,Y.

TITLE Direct Submission

JOURNAL Submitted (20-JUN-2023) Department of Infectious Diseases, Peking

University People's Hospital, Xizhimen South Street, Beijing

100044, China

COMMENT ##Assembly-Data-START##

Sequencing Technology :: Sanger dideoxy sequencing

##Assembly-Data-END##

FEATURES Location/Qualifiers

source 1..1749

/organism="Influenza B virus"

/mol_type="viral cRNA"

/strain="B/Beijing/2336/2022"

/isolate="2336"

/isolation_source="nasal swab"

/host="Homo sapiens"

/db_xref="taxon:11520"

/segment="4"

/country="China: Beijing"

/collection_date="30-Aug-2022"

gene 1..1749

/gene="HA"

CDS 1..1749

/gene="HA"

/function="receptor binding and fusion protein"

/codon_start=1

/product="hemagglutinin"

/protein_id="WJE87866"

/translation="MKAIIVLLMVVTSNADRICTGITSSNSPHVVKTATQGEVNVTGV

IPLTTTPTKSHFANLKGTETRGKLCPKCLNCTDLDVALGRPKCTGKIPSARVSILHEV

RPVTSGCFPIMHDRTKIRQLPNLLRGYEHVRLSTQNVINTEDAPGGPYEIGTSGSCLN

ITNEKGFFATMAWAVPKNKTATNPLTIEVPYICTEEEDQITVWGFHSDDETQMARLYG

DSKPQKFTSSANGMTTHYVSQIGGFPNQTEDGGLPQSGRIVVDYMVQKSGKTGTITYQ

RGILLPQKVWCASGKSKVIKGSLPLIGEADCLHEKYGGLNKSKPYYTGEHAKAIGNCP

IWVKTPLKLANGTKYRPPAKLLKERGFFGAIAGFLEGGWEGMIAGWHGYTSHGAHGVA

VAADLKSTQEAINKITKNLNSLSELEVKNLQRLSGAMDELHNEILELDEKVDDLRADT

ISSQIELAVLLSNEGIINSEDEHLLALERKLKKMLGPSAVEIGNGCFETKHKCNQTCL

DRIAAGTFDAGEFSLPTFDSLNITAASLNDDGLDNHTILLYYSTAASSLAVTLMIAIF

VVYMVSRDNVSCSICL"

ORIGIN

1 atgaaggcaa taattgtact actcatggta gtaacatcca atgcagatcg aatctgcact

61 gggataacat cgtcaaactc accacatgtc gtcaaaactg ctactcaagg ggaggtcaat

121 gtgaccggtg taataccact gacaacaaca cccaccaaat ctcattttgc aaatctcaaa

181 ggaacagaaa ccagggggaa actatgccca aaatgcctaa actgcacaga tctggatgta

241 gccttgggca gaccaaaatg cacagggaaa ataccctccg caagggtttc aatactccat

301 gaagtcagac ctgttacatc tgggtgcttt cctataatgc atgatagaac aaaaattaga

361 cagctgccta accttctccg aggatacgaa catgtcaggt tatcaactca aaacgttatc

421 aatacagaag atgcaccagg aggaccctac gaaattggaa cctcagggtc ttgcctcaac

481 attaccaatg aaaaaggatt cttcgcaaca atggcttggg ccgtcccaaa aaacaaaaca

541 gcaacaaatc cattaacaat agaagtacca tacatttgta cagaagaaga agaccaaatt

601 accgtttggg ggttccactc tgacgacgag acccaaatgg caaggctcta tggggattca

661 aagccccaga agttcacctc atctgccaac ggaatgacca cacattacgt ctcacagatt

721 ggtggcttcc caaatcaaac agaagacgga ggactaccac aaagtggcag aattgttgtt

781 gattacatgg tgcaaaaatc tggaaaaaca ggaacaatta cctatcaaag aggtatttta

841 ttgcctcaaa aggtgtggtg cgcaagtggc aagagcaagg taataaaagg atccttgccc

901 ttaattggag aagcagattg ccttcatgaa aaatacggtg gattaaacaa aagcaagcct

961 tactacacag gggaacatgc aaaggccata ggaaattgcc caatatgggt gaaaacaccc

1021 ttgaagctgg ccaatggaac caaatataga cctcctgcaa aactattaaa ggaaagaggt

1081 ttcttcggag ccattgctgg tttcttagag ggaggatggg aaggaatgat tgcaggttgg

1141 cacggataca catcccatgg ggcacatgga gtagcagtgg cagctgacct taagagcact

1201 caagaggcca taaacaagat aacaaaaaat ctcaactctt tgagtgagct ggaagtaaag

1261 aatcttcaaa gactaagcgg tgccatggat gaactccaca acgaaatact agaactagat

1321 gagaaagtgg atgatctcag agctgataca ataagctcac aaatagaact cgcagtcctg

1381 ctttccaatg aaggaataat aaacagtgaa gatgaacatc tcttggcgct tgaaagaaag

1441 ctgaagaaaa tgctgggccc ctctgctgta gagataggga atggatgctt tgaaaccaaa

1501 cacaagtgca accagacctg tctcgacaga atagctgctg gtacctttga tgcaggagaa

1561 ttttctctcc ccacctttga ttcactgaat attactgctg catctttaaa tgacgatgga

1621 ttggacaatc atactatact gctttactac tcaactgctg cctccagttt ggctgtaaca

1681 ctgatgatag ctatctttgt tgtttatatg gtctccagag acaatgtttc ttgctccatt

1741 tgtctataa

//

LOCUS OR145896 1749 bp cRNA linear VRL 20-JUN-2023

DEFINITION Influenza B virus (B/Beijing/2337/2022) segment 4 hemagglutinin

(HA) gene, complete cds.

ACCESSION OR145896

VERSION OR145896

KEYWORDS .

SOURCE Influenza B virus

ORGANISM Influenza B virus

Viruses; Riboviria; Orthornavirae; Negarnaviricota;

Polyploviricotina; Insthoviricetes; Articulavirales;

Orthomyxoviridae; Betainfluenzavirus; Betainfluenzavirus

influenzae.

REFERENCE 1 (bases 1 to 1749)

AUTHORS Wang,Y.

TITLE Direct Submission

JOURNAL Submitted (20-JUN-2023) Department of Infectious Diseases, Peking

University People's Hospital, Xizhimen South Street, Beijing

100044, China

COMMENT ##Assembly-Data-START##

Sequencing Technology :: Sanger dideoxy sequencing

##Assembly-Data-END##

FEATURES Location/Qualifiers

source 1..1749

/organism="Influenza B virus"

/mol_type="viral cRNA"

/strain="B/Beijing/2337/2022"

/isolate="2337"

/isolation_source="nasal swab"

/host="Homo sapiens"

/db_xref="taxon:11520"

/segment="4"

/country="China: Beijing"

/collection_date="30-Aug-2022"

gene 1..1749

/gene="HA"

CDS 1..1749

/gene="HA"

/function="receptor binding and fusion protein"

/codon_start=1

/product="hemagglutinin"

/protein_id="WJE87867"

/translation="MKAIIVLLMVVTSNADRICTGITSSNSPHVVKTATQGEVNVTGV

IPLTTTPTKSHFANLKGTETRGKLCPKCLNCTDLDVALGRPKCTGKIPSARVSILHEV

RPVTSGCFPIMHDRTKIRQLPNLLRGYEHVRLSTHNVINAEDAPGGPYEIGTSGSCPN

ITNGKGFFATMAWAVPKNKTATNPLTIEVPYICTEEEDQITVWGFHSDDKTQMAKLYG

DSKPQKFTSSANGMTTHYVSQIGGFPNQTEDGGLQQSGRIVVDYMVQKSGKTGTITYQ

RGILLPQKVWCASGKSKVIKGSLPLIGEADCLHEKYGGLNKSKPYYTGEHAKAIGNCP

IWVKTPLKLANGTKYRPPAKLLKERGFFGAIAGFLEGGWEGMIAGWHGYTSHGAHGVA

VAADLKSTQEAINKITKNLNSLSELEVKNLQRLSGAMDELHNEILELDEKVDDLRADT

ISSQIELAVLLSNEGIINSEDEHLLALERKLKKMLGPSAVEIGNGCFETKHKCNQTCL

DRIAAGTFDAGEFSLPTFDSLNITAASLNDDGLDNHTILLYYSTAASSLAVTLMIAIF

VVYMVSRDNVSCSICL"

ORIGIN

1 atgaaggcaa taattgtact actcatggta gtaacatcca atgcagatcg aatctgcact

61 gggataacat cgtcaaactc accacatgtc gtcaaaactg ctactcaagg ggaggtcaat

121 gtgaccggtg taataccact gacaacaaca cccaccaaat ctcattttgc aaatctcaaa

181 ggaacagaaa ccagggggaa actatgccca aaatgcctca actgcacaga tctggatgta

241 gccttgggca gaccaaaatg cacagggaaa ataccctctg caagggtttc aatactccat

301 gaagtcagac ctgttacatc tgggtgcttt cctataatgc atgatagaac aaaaattaga

361 cagctgccta accttctccg aggatacgaa catgtcagat tatcaactca caacgttatc

421 aatgcagaag atgcaccagg aggaccctac gaaattggaa cctcagggtc ttgccctaac

481 attaccaatg gaaaaggatt cttcgcaaca atggcttggg ccgtcccaaa aaacaaaaca

541 gcaacaaatc cattaacaat agaagtacca tacatttgta cagaagaaga agaccaaatt

601 accgtttggg ggttccactc tgacgacaag acccaaatgg caaagctcta tggggactca

661 aagccccaga agttcacctc atctgccaac ggaatgacca cacattacgt ttcacagatt

721 ggtggcttcc caaatcaaac agaagacgga ggattacaac aaagtggcag aattgttgtt

781 gattacatgg tgcaaaaatc tggaaaaaca ggaacaatta cctatcaaag aggtatttta

841 ttgcctcaaa aggtgtggtg cgcaagtggc aagagcaagg tgataaaagg atccttgccc

901 ttaattggag aagcagattg cctccatgaa aaatacggtg gattaaacaa aagcaagcct

961 tactacacag gggaacatgc aaaggccata ggaaattgcc caatatgggt gaaaacaccc

1021 ttgaagctgg ccaatggaac caaatataga cctcctgcaa aactattaaa ggaaagaggt

1081 ttcttcggag ccattgctgg tttcttagag ggaggatggg aaggaatgat tgcaggttgg

1141 cacggataca catctcatgg ggcacatgga gtagcggtgg cagctgacct taagagcact

1201 caagaggcca taaacaagat aacaaaaaat ctcaactctt tgagtgagct ggaagtaaag

1261 aatcttcaaa gactaagcgg tgccatggat gaactccaca acgaaatact agaactagat

1321 gagaaagtgg atgatctcag agctgataca ataagctcac aaatagaact cgcagtcctg

1381 ctttccaatg aaggaataat aaacagtgaa gatgaacatc tcttggcgct tgaaagaaag

1441 ctgaagaaaa tgctgggccc ctctgctgta gagataggga atggatgctt tgaaaccaaa

1501 cacaagtgca accagacctg tctcgacaga atagctgctg gtacctttga tgcaggagaa

1561 ttttctctcc ccacctttga ttcactaaat attactgctg catctttaaa tgatgatgga

1621 ttggacaatc atactatact gctttactac tcaactgctg cctccagttt ggctgtaaca

1681 ctgatgatag ctatctttgt tgtttatatg gtctccagag acaatgtttc ttgctccatt

1741 tgtctataa

//

LOCUS OR145897 1749 bp cRNA linear VRL 20-JUN-2023

DEFINITION Influenza B virus (B/Beijing/2338/2022) segment 4 hemagglutinin

(HA) gene, complete cds.

ACCESSION OR145897

VERSION OR145897

KEYWORDS .

SOURCE Influenza B virus

ORGANISM Influenza B virus

Viruses; Riboviria; Orthornavirae; Negarnaviricota;

Polyploviricotina; Insthoviricetes; Articulavirales;

Orthomyxoviridae; Betainfluenzavirus; Betainfluenzavirus

influenzae.

REFERENCE 1 (bases 1 to 1749)

AUTHORS Wang,Y.

TITLE Direct Submission

JOURNAL Submitted (20-JUN-2023) Department of Infectious Diseases, Peking

University People's Hospital, Xizhimen South Street, Beijing

100044, China

COMMENT ##Assembly-Data-START##

Sequencing Technology :: Sanger dideoxy sequencing

##Assembly-Data-END##

FEATURES Location/Qualifiers

source 1..1749

/organism="Influenza B virus"

/mol_type="viral cRNA"

/strain="B/Beijing/2338/2022"

/isolate="2338"

/isolation_source="nasal swab"

/host="Homo sapiens"

/db_xref="taxon:11520"

/segment="4"

/country="China: Beijing"

/collection_date="30-Aug-2022"

gene 1..1749

/gene="HA"

CDS 1..1749

/gene="HA"

/function="receptor binding and fusion protein"

/codon_start=1

/product="hemagglutinin"

/protein_id="WJE87868"

/translation="MKAIIVLLMVVTSNADRICTGITSSNSPHVVKTATQGEVNVTGV

IPLTTTPTKSHFANLKGTETRGKLCPKCLNCTDLDVALGRPKCTGKIPSARVSILHEV

RPVTSGCFPIMHDRTKIRQLPNLLRGYEHVRLSTQNVINTEDAPGGPYEIGTSGSCLN

ITNGKGFFATMAWAVPKNKTATNPLTIEVPYICTEEEDQITVWGFHSDDETQMARLYG

DSKPQKFTSSANGVTTHYVSQIGGFPNQTEDGGLPQSGRIVVDYMVQKSGKTGTITYQ

RGILLPQKVWCASGKSKVIKGSLPLIGEADCLHEKYGGLNKSKPYYTGEHAKAIGNCP

IWVKTPLKLANGTKYRPPAKLLKERGFFGAIAGFLEGGWEGMIAGWHGYTSHGAHGVA

VAADLKSTQEAINKITKNLNSLSELEVKNLQRLRSAMDELHNEILELDEKVDDLRADT

ISSQIELAVLLSNEGIINSEDEHLLALERKLKKMLGPSAVEIGNGCFETKHKCNQTCL

DRIAAGTFDAGEFSLPTFDSLNITAASLNDDGLDNHTILLYYSTAASSLAVTLMIAIF

VVYMVSRDNVSCSICL"

ORIGIN

1 atgaaggcaa taattgtact actcatggta gtaacatcca atgcagatcg aatctgcact

61 gggataacat cgtcaaactc accacatgtc gtcaaaactg ctactcaagg ggaggtcaat

121 gtgaccggtg taataccact gacaacaaca cccaccaaat ctcattttgc aaatctcaaa

181 ggaacagaaa ccagggggaa actatgccca aaatgcctaa actgcacaga tctggatgta

241 gccctgggca gaccaaaatg cacagggaaa ataccctctg caagggtttc aatactccat

301 gaagtcagac ctgttacatc tgggtgcttt cctataatgc atgatagaac aaaaattaga

361 cagctgccta accttctccg aggatacgaa catgtcaggt tatcaactca aaacgttatc

421 aatacagaag atgcaccagg aggaccctac gaaattggaa cctcagggtc ttgcctcaac

481 attaccaatg gaaaaggatt cttcgcaaca atggcttggg ccgtcccaaa aaacaaaaca

541 gcaacaaatc cattaacaat agaagtacca tacatttgta cagaagaaga agaccaaatt

601 accgtttggg ggttccactc tgacgacgag acccaaatgg caaggctcta tggggattca

661 aagccccaaa agttcacctc atctgccaac ggagtgacca cacattacgt ctcacagatt

721 ggtggcttcc caaatcaaac agaagacgga ggactaccac aaagtggcag aattgttgtt

781 gattacatgg tgcaaaaatc tggaaaaaca ggaacaatta cctatcaaag aggtatttta

841 ttgcctcaaa aggtgtggtg cgcaagtggc aagagcaagg taataaaagg atccttgccc

901 ttaattggag aagcagattg cctccatgaa aaatacggtg gattaaacaa aagcaagcct

961 tactacacag gggaacatgc aaaggccata ggaaattgcc caatatgggt gaaaacaccc

1021 ttgaagctgg ccaatggaac caaatataga cctcctgcaa aactattaaa ggaaagaggt

1081 ttcttcggag ccattgctgg tttcttagag ggaggatggg aaggaatgat tgcaggttgg

1141 cacggataca catcccatgg ggcacatgga gtagcagtgg cagctgacct taagagcact

1201 caagaggcca taaacaagat aacaaaaaat ctcaactctt tgagtgagct ggaagtaaag

1261 aatcttcaaa gactaaggag tgccatggat gaactccaca acgaaatact agaactagat

1321 gagaaagtgg atgatctcag agctgataca ataagctcac aaatagaact cgcagtcctg

1381 ctttccaacg aaggaataat aaacagtgag gatgaacatc tcttggcgct tgaaagaaag

1441 ctgaagaaaa tgctgggccc ctctgctgta gagataggga atggatgctt tgaaaccaaa

1501 cacaagtgca accagacctg tctcgacaga atagctgctg gtacctttga tgcaggagaa

1561 ttttctctcc ccacctttga ttcactgaat attactgctg catctttaaa tgacgatgga

1621 ttggacaatc atactatact gctttactac tcaactgctg cctccagttt ggctgtaaca

1681 ctgatgatag ctatctttgt tgtttatatg gtctccagag acaatgtttc ttgctccatt

1741 tgtctataa

//

LOCUS OR145898 1749 bp cRNA linear VRL 20-JUN-2023

DEFINITION Influenza B virus (B/Beijing/2339/2022) segment 4 hemagglutinin

(HA) gene, complete cds.

ACCESSION OR145898

VERSION OR145898

KEYWORDS .

SOURCE Influenza B virus

ORGANISM Influenza B virus

Viruses; Riboviria; Orthornavirae; Negarnaviricota;

Polyploviricotina; Insthoviricetes; Articulavirales;

Orthomyxoviridae; Betainfluenzavirus; Betainfluenzavirus

influenzae.

REFERENCE 1 (bases 1 to 1749)

AUTHORS Wang,Y.

TITLE Direct Submission

JOURNAL Submitted (20-JUN-2023) Department of Infectious Diseases, Peking

University People's Hospital, Xizhimen South Street, Beijing

100044, China

COMMENT ##Assembly-Data-START##

Sequencing Technology :: Sanger dideoxy sequencing

##Assembly-Data-END##

FEATURES Location/Qualifiers

source 1..1749

/organism="Influenza B virus"

/mol_type="viral cRNA"

/strain="B/Beijing/2339/2022"

/isolate="2339"

/isolation_source="nasal swab"

/host="Homo sapiens"

/db_xref="taxon:11520"

/segment="4"

/country="China: Beijing"

/collection_date="30-Aug-2022"

gene 1..1749

/gene="HA"

CDS 1..1749

/gene="HA"

/function="receptor binding and fusion protein"

/codon_start=1

/product="hemagglutinin"

/protein_id="WJE87869"

/translation="MKAIIVLLMVVTSNADRICTGITSSNSPHVVKTATQGEVNVTGV

IPLTTTPTKSHFANLKGTETRGKLCPKCLNCTDLDVALGRPKCTGKIPSARVSILHEV

RPVTSGCFPIMHDRTKIRQLPNLLRGYEHVRLSTQNVINTEDAPGGPYEIGTSGSCLN

ITNGKGFFATMAWAVPKNKTATNPLTIEVPYICTEEEDQITVWGFHSDDETQMARLYG

DSKPQKFTSSANGVTTHYVSQIGGFPNQTEDGGLPQSGRIVVDYMVQKSGKTGTITYQ

RGILLPQKVWCASGKSKVIKGSLPLIGEADCLHEKYGGLNKSKPYYTGEHAKAIGNCP

IWVKTPLKLANGTKYRPPAKLLKERGFFGAIAGFLEGGWEGMIAGWHGYTSHGAHGVA

VAADLKSTQEAINKITKNLNSLSELEVKNLQRLASAMDELHNEILELDEKVDDLRADT

ISSQIELAVLLSNEGIINSEDEHLLALERKLKKMLGPSAVEIGNGCFETKHKCNQTCL

DRIAAGTFDAGEFSLPTFDSLNITAASLNDDGLDNHTILLYYSTAASSLAVTLMIAIF

VVYMVSRDNVSCSICL"

ORIGIN

1 atgaaggcaa taattgtact actcatggta gtaacatcca atgcagatcg aatctgcact

61 gggataacat cgtcaaactc accacatgtc gtcaaaactg ctactcaagg ggaggtcaat

121 gtgaccggtg taataccact gacaacaaca cccaccaaat ctcattttgc aaatctcaaa

181 ggaacagaaa ccagggggaa actatgccca aaatgcctaa actgcacaga tctggatgta

241 gccttgggca gaccaaaatg cacagggaaa ataccctctg caagggtttc aatactccat

301 gaagtcagac ctgttacatc tgggtgcttt cctataatgc atgatagaac aaaaattaga

361 cagctgccta accttctccg aggatacgaa catgtcaggt tatcaactca aaacgttatc

421 aatacagaag atgcaccagg aggaccctac gaaattggaa cctcagggtc ttgcctcaac

481 attaccaatg gaaaaggatt cttcgcaaca atggcttggg ccgtcccaaa aaacaaaaca

541 gcaacaaatc cattaacaat agaagtacca tacatttgta cagaagaaga agaccaaatt

601 accgtttggg ggttccactc tgacgacgag acccaaatgg caaggctcta tggggattca

661 aagccccaga agttcacctc atctgccaac ggagtgacca cacattacgt ctcacagatt

721 ggtggcttcc caaatcaaac agaagacgga ggactaccac aaagtggcag aattgttgtt

781 gattacatgg tgcaaaaatc tggaaaaaca ggaacaatta cctatcaaag aggtatttta

841 ttgcctcaaa aggtgtggtg cgcaagtggc aagagcaagg taataaaagg atccttgccc

901 ttaattggag aagcagattg cctccatgaa aaatacggtg gattaaacaa aagcaagcct

961 tactacacag gggaacatgc aaaggccata ggaaattgcc caatatgggt gaaaacaccc

1021 ttgaagctgg ccaatggaac caaatataga cctcctgcaa aactattaaa ggaaagaggt

1081 ttcttcggag ccattgctgg tttcttagag ggaggatggg aaggaatgat tgcaggttgg

1141 cacggataca catcccatgg ggcacatgga gtagcagtgg cagctgacct taagagcact

1201 caagaggcca taaacaagat aacaaaaaat ctcaactctt tgagtgagct ggaagtaaag

1261 aatcttcaaa gactggccag tgccatggat gaactccaca acgaaatact agaactagat

1321 gagaaagtgg atgatctcag agccgataca ataagctcac aaatagaact cgcagtcctg

1381 ctttccaatg aaggaataat aaacagtgaa gatgaacatc tcttggcgct tgaaagaaag

1441 ctgaagaaaa tgctgggccc ctctgctgta gagataggga atggatgctt tgaaaccaaa

1501 cacaagtgca accagacctg tctcgacaga atagctgctg gtacctttga tgcaggagaa

1561 ttttctctcc ccacctttga ttcactgaat attactgctg catctttaaa tgacgatgga

1621 ttggacaatc atactatact gctttactac tcaactgctg cctccagttt ggctgtaaca

1681 ctgatgatag ctatctttgt tgtttatatg gtctccagag acaatgtttc ttgctccatt

1741 tgtctataa

//

LOCUS OR145899 1749 bp cRNA linear VRL 20-JUN-2023

DEFINITION Influenza B virus (B/Beijing/2340/2022) segment 4 hemagglutinin

(HA) gene, complete cds.

ACCESSION OR145899

VERSION OR145899

KEYWORDS .

SOURCE Influenza B virus

ORGANISM Influenza B virus

Viruses; Riboviria; Orthornavirae; Negarnaviricota;

Polyploviricotina; Insthoviricetes; Articulavirales;

Orthomyxoviridae; Betainfluenzavirus; Betainfluenzavirus

influenzae.

REFERENCE 1 (bases 1 to 1749)

AUTHORS Wang,Y.

TITLE Direct Submission

JOURNAL Submitted (20-JUN-2023) Department of Infectious Diseases, Peking

University People's Hospital, Xizhimen South Street, Beijing

100044, China

COMMENT ##Assembly-Data-START##

Sequencing Technology :: Sanger dideoxy sequencing

##Assembly-Data-END##

FEATURES Location/Qualifiers

source 1..1749

/organism="Influenza B virus"

/mol_type="viral cRNA"

/strain="B/Beijing/2340/2022"

/isolate="2340"

/isolation_source="nasal swab"

/host="Homo sapiens"

/db_xref="taxon:11520"

/segment="4"

/country="China: Beijing"

/collection_date="30-Aug-2022"

gene 1..1749

/gene="HA"

CDS 1..1749

/gene="HA"

/function="receptor binding and fusion protein"

/codon_start=1

/product="hemagglutinin"

/protein_id="WJE87870"

/translation="MKAIIVLLMVVTSNADRICTGITSSNSPHVVKTATQGEVNVTGV

IPLTTTPTKSHFANLKGTETRGKLCPKCLNCTDLDVALGRPKCTGKIPSARVSILHEV

RPVTSGCFPIMHDRTKIRQLPNLLRGYEHVRLSTQNVINTEDAPGGPYEIGTSGSCLN

ITNGKGFFATMAWAVPKNQTATNPLTIEVPYICTEEEDQITVWGFHSDDETQMAKLYG

DSKPQKFTSSANGVTTHYVSQIGGFPNQTEDGGLPQSGRIVVDYMVQKSGKTGTITYQ

RGILLPQKVWCASGKSKVIKGSLPLIGEADCLHEKYGGLNKSKPYYTGEHAKAIGNCP

IWVKTPLKLANGTKYRPPAKLLKERGFFGAIAGFLEGGWEGMIAGWHGYTSHGAHGVA

VAADLKSTQEAINKITKNLNSLSELEVKNLQRLASAMDELHNEILELDEKVDDLRADT

ISSQIELAVLLSNEGIINSEDEHLLALERKLKKMLGPSAVEIGNGCFETKHKCNQTCL

DRIAAGTFDAGEFSLPTFDSLNITAASLNDDGLDNHTILLYYSTAASSLAVTLMIAIF

VVYMVSRDNVSCSICL"

ORIGIN

1 atgaaggcaa taattgtact actcatggta gtaacatcca atgcagatcg aatctgcact

61 gggataacat cgtcaaactc accacatgtc gtcaaaactg ctactcaagg ggaggtcaat

121 gtgaccggtg taataccact gacaacaaca cccaccaaat ctcattttgc aaatctcaaa

181 ggaacagaaa ccagggggaa actatgccca aaatgcctaa actgcacaga tctggatgta

241 gccttgggca gaccaaaatg cacagggaaa ataccctctg caagggtttc aatactccat

301 gaagtcagac ctgttacatc tgggtgcttt cctataatgc atgatagaac aaaaattaga

361 cagctgccta accttctccg aggatacgaa catgtcaggt tatcaactca aaacgttatc

421 aatacagaag atgcaccagg aggaccctac gaaattggaa cctcagggtc ttgcctcaac

481 attaccaatg gaaaaggatt cttcgcaaca atggcttggg ccgtcccaaa aaaccaaaca

541 gcaacaaatc cattaacaat agaagtacca tacatttgta cagaagaaga agaccaaatt

601 accgtttggg ggttccactc tgacgacgag acccaaatgg ccaagctcta tggggattca

661 aagccccaga agttcacctc atctgccaac ggagtgacca cacattacgt ctcacagatt

721 ggtggcttcc caaatcaaac agaagacgga ggactaccac aaagtggcag aattgttgtt

781 gattacatgg tgcaaaaatc tggaaaaaca ggaacaatta cctatcaaag aggtatttta

841 ttgcctcaaa aggtgtggtg cgcaagtggc aagagcaagg taataaaagg atccttgccc

901 ttaattggag aagcagattg cctccatgaa aaatacggtg gattaaacaa aagcaagcct

961 tactacacag gggaacatgc aaaggccata ggaaattgcc caatatgggt gaaaacaccc

1021 ttgaagctgg ccaatggaac caaatataga cctcctgcaa aattattaaa ggaaagaggt

1081 ttcttcggag ccattgctgg tttcttagag ggaggatggg aaggaatgat tgcaggttgg

1141 cacggataca catcccatgg ggcacatgga gtagcagtgg cagctgacct taagagcact

1201 caagaggcca taaacaagat aacaaaaaat ctcaactctt tgagtgagct ggaagtaaag

1261 aatcttcaaa gactggccag tgccatggat gaactccata acgaaatact agaactagat

1321 gagaaagtgg atgatctcag agctgataca ataagctcac aaatagaact cgcagtcctg

1381 ctttccaatg aaggaataat aaacagtgaa gatgaacatc tcttggcgct tgaaagaaag

1441 ctgaagaaaa tgctgggccc ctctgctgta gagataggga atggatgctt tgaaaccaaa

1501 cacaagtgca accagacctg tctcgacaga atagctgctg gtacctttga tgcaggagaa

1561 ttttctctcc ccacctttga ttcactgaat attactgctg catctttaaa tgacgatgga

1621 ttggacaatc atactatact gctttactac tcaactgctg cctccagttt ggctgtaaca

1681 ctgatgatag ctatctttgt tgtttatatg gtctccagag acaatgtttc ttgctccatt

1741 tgtctataa

//

LOCUS OR145900 1749 bp cRNA linear VRL 20-JUN-2023

DEFINITION Influenza B virus (B/Beijing/2341/2022) segment 4 hemagglutinin

(HA) gene, complete cds.

ACCESSION OR145900

VERSION OR145900

KEYWORDS .

SOURCE Influenza B virus

ORGANISM Influenza B virus

Viruses; Riboviria; Orthornavirae; Negarnaviricota;

Polyploviricotina; Insthoviricetes; Articulavirales;

Orthomyxoviridae; Betainfluenzavirus; Betainfluenzavirus

influenzae.

REFERENCE 1 (bases 1 to 1749)

AUTHORS Wang,Y.

TITLE Direct Submission

JOURNAL Submitted (20-JUN-2023) Department of Infectious Diseases, Peking

University People's Hospital, Xizhimen South Street, Beijing

100044, China

COMMENT ##Assembly-Data-START##

Sequencing Technology :: Sanger dideoxy sequencing

##Assembly-Data-END##

FEATURES Location/Qualifiers

source 1..1749

/organism="Influenza B virus"

/mol_type="viral cRNA"

/strain="B/Beijing/2341/2022"

/isolate="2341"

/isolation_source="nasal swab"

/host="Homo sapiens"

/db_xref="taxon:11520"

/segment="4"

/country="China: Beijing"

/collection_date="30-Aug-2022"

gene 1..1749

/gene="HA"

CDS 1..1749

/gene="HA"

/function="receptor binding and fusion protein"

/codon_start=1

/product="hemagglutinin"

/protein_id="WJE87871"

/translation="MKAIIVLLMVVTSNADRICTGITSSNSPHVVKTATQGEVNVTGV

IPLTTTPTKSHFANLKGTETRGKLCPKCLNCTDLDVALGRPKCTGKIPSARVSILHEV

RPVTSGCFPIMHDRTKIRQLPNLLRGYEHVRLSTQNVINTEDAPGGPYEIGTSGSCLN

ITNGKGFFATMAWAVPKNKTTTNPLTIEVPYICTEEEDQITVWGFHSDDETQMARLYG

DSKPQKFTSSANGVTTHYVSQIGGFPNQTEDGGLPQSGRIVVDYMVQKSGKTGTITYQ

RGILLPQKVWCASGKSKVIKGSLPLIGEADCLHEKYGGLNKSKPYYTGEHAKAIGNCP

IWVKTPLKLANGTKYRPPAKLLKERGFFGAIAGFLEGGWEGMIAGWHGYTSHGAHGVA

VAADLKSTQEAINKITKNLNSLSELEVKNLQRLSGAMDELHNEILELDEKVDDLRADT

ISSQIELAVLLSNEGIINSEDEHLLALERKLKKMLGPSAVEIGNGCFETKHKCNQTCL

DRIAAGTFDAGEFSLPTFDSLNITAASLNDDGLDNHTILLYYSTAASSLAVTLMIAIF

VVYMVSRDNVSCSICL"

ORIGIN

1 atgaaggcaa taattgtact actcatggta gtaacatcca atgcagatcg aatctgcact

61 gggataacat cgtcaaactc accacatgtc gtcaaaactg ctactcaagg ggaggtcaat

121 gtgaccggtg taataccact gacaacaaca cccaccaaat ctcattttgc aaatctcaaa

181 ggaacagaaa ccagggggaa actatgccca aaatgcctaa actgcacaga tctggatgta

241 gccttgggca gaccaaaatg cacagggaaa ataccctctg caagggtttc aatactccat

301 gaagtcagac ctgttacatc tgggtgcttt cctataatgc atgatagaac aaaaattaga

361 cagctgccta accttctccg aggatacgaa catgtcaggt tatcaactca aaacgttatc

421 aatacagaag atgcaccagg aggaccctac gaaattggaa cctcagggtc ttgcctcaac

481 attaccaatg gaaaaggatt cttcgcaaca atggcttggg ccgtcccaaa aaacaaaaca

541 acaacaaatc cattaacaat agaagtacca tacatttgta cagaagaaga agaccaaatt

601 accgtttggg ggttccactc tgacgacgag acccaaatgg caaggctcta tggggattca

661 aagccccaga agttcacctc atctgccaac ggagtgacca cacattacgt ctcacagatt

721 ggtggcttcc caaatcaaac agaagacgga ggactaccac aaagtggcag aattgttgtt

781 gattacatgg tgcaaaaatc tggaaaaaca ggaacaatta cctatcaaag aggtatttta

841 ttgcctcaaa aggtgtggtg cgcaagtggc aagagcaagg taataaaagg atccttgccc

901 ttaattggag aagcagattg ccttcatgaa aaatacggtg gattaaacaa aagcaagcct

961 tactacacag gggaacatgc aaaggccata ggaaattgcc caatatgggt gaaaacaccc

1021 ttgaagctgg ccaatggaac caaatataga cctcctgcaa aactattaaa ggaaagaggt

1081 ttcttcggag ccattgctgg tttcttagag ggaggatggg aaggaatgat tgcaggttgg

1141 cacggataca catcccatgg ggcacatgga gtagcagtgg cagctgacct taagagcact

1201 caagaggcca taaacaagat aacaaaaaat ctcaactctt tgagtgagct ggaagtaaag

1261 aatcttcaaa gactaagcgg tgccatggat gaactccaca acgaaatact agaactagat

1321 gagaaagtgg atgatctcag agctgataca ataagctcac aaatagaact cgcagtcttg

1381 ctttccaatg aaggaataat aaacagtgaa gatgaacatc tcttggcgct tgaaagaaag

1441 ctgaagaaaa tgctgggccc ctctgctgta gagataggga atggatgctt tgaaaccaaa

1501 cacaagtgca accagacctg tctcgacaga atagctgctg gtacctttga tgcaggagaa

1561 ttttctctcc ctacctttga ttcactgaat attactgctg catctttaaa tgacgatgga

1621 ttggacaatc atactatact gctttactac tcaactgctg cctccagttt ggctgtaaca

1681 ctgatgatag ctatctttgt tgtttatatg gtctccagag acaatgtttc ttgctccatt

1741 tgtctataa

//

LOCUS OR145901 1749 bp cRNA linear VRL 20-JUN-2023

DEFINITION Influenza B virus (B/Beijing/2342/2022) segment 4 hemagglutinin

(HA) gene, complete cds.

ACCESSION OR145901

VERSION OR145901

KEYWORDS .

SOURCE Influenza B virus

ORGANISM Influenza B virus

Viruses; Riboviria; Orthornavirae; Negarnaviricota;

Polyploviricotina; Insthoviricetes; Articulavirales;

Orthomyxoviridae; Betainfluenzavirus; Betainfluenzavirus

influenzae.

REFERENCE 1 (bases 1 to 1749)

AUTHORS Wang,Y.

TITLE Direct Submission

JOURNAL Submitted (20-JUN-2023) Department of Infectious Diseases, Peking

University People's Hospital, Xizhimen South Street, Beijing

100044, China

COMMENT ##Assembly-Data-START##

Sequencing Technology :: Sanger dideoxy sequencing

##Assembly-Data-END##

FEATURES Location/Qualifiers

source 1..1749

/organism="Influenza B virus"

/mol_type="viral cRNA"

/strain="B/Beijing/2342/2022"

/isolate="2342"

/isolation_source="nasal swab"

/host="Homo sapiens"

/db_xref="taxon:11520"

/segment="4"

/country="China: Beijing"

/collection_date="30-Aug-2022"

gene 1..1749

/gene="HA"

CDS 1..1749

/gene="HA"

/function="receptor binding and fusion protein"

/codon_start=1

/product="hemagglutinin"

/protein_id="WJE87872"

/translation="MKAIIVLLMVVTSNADRICTGITSSNSPHVVKTATQGEVNVTGV

IPLTTTPTKSHFANLKGTETRGKLCPKCLNCTDLDVALGRPKCTGKIPSARVSILHEV

RPVTSGCFPIMHDRTKIRQLPNLLRGYEHVRLSTQNVINTEDAPGGPYEIGTSGSCLN

ITNGKGFFATMAWAVPKNKTATNPLTIEVPYICTEEEDQITVWGFHSDDETQMARLYG

DSKPQKFTSSANGVTTHYVSQIGGFPNQTEDGGLPQSGRIVVDYMVQKSGKTGTITYQ

RGILLPQKVWCASGKSKVIKGSLPLIGEADCLHEKYGGLNKSKPYYTGEHAKAIGNCP

IWVKTPLKLANGTKYRPPAKLLKERGFFGAIAGFLEGGWEGMIAGWHGYTSHGAHGVA

VAADLKSTQEAINKITKNLNSLSELEVKNLQRLSSAMDELHNEILELDEKVDDLRADT

ISSQIELAVLLSNEGIINSEDEHLLALERKLKKMLGPSAVEIGNGCFETKHKCNQTCL

DRIAAGTFDAGEFSLPTFDSLNITAASLNDDGLDNHTILLYYSTAASSLAVTLMIAIF

VVYMVSRDNVSCSICL"

ORIGIN

1 atgaaggcaa taattgtact actcatggta gtaacatcca atgcagatcg aatctgcact

61 gggataacat cgtcaaactc accacatgtc gtcaaaactg ctactcaagg ggaggtcaat

121 gtgaccggtg taataccact gacaacaaca cccactaaat ctcattttgc aaatctcaaa

181 ggaacagaaa ccagggggaa actatgccca aaatgcctaa actgcacaga tctggatgta

241 gccttgggca gaccaaaatg cacagggaaa ataccctctg caagggtttc aatactccat

301 gaagtcagac ctgttacatc tgggtgcttt cctataatgc atgatagaac aaaaattaga

361 cagctgccta accttctccg aggatacgaa catgtcaggt tatcaactca aaacgttatc

421 aatacagaag atgcaccagg aggaccctac gaaattggaa cctcagggtc ttgcctcaac

481 attaccaatg gaaaaggatt cttcgcaaca atggcttggg ccgtcccaaa aaacaaaaca

541 gcaacaaatc cattaacaat agaagtacca tacatttgta cagaagaaga agaccaaatt

601 accgtttggg ggttccactc tgacgacgag acccaaatgg caaggctcta tggggattca

661 aagccccaga agttcacctc atctgccaac ggagtgacca cacattacgt ctcacagatt

721 ggtggcttcc caaatcaaac agaagacgga ggactaccac aaagtggcag aattgttgtt

781 gattacatgg tgcaaaaatc tggaaaaaca ggaacaatta cctatcaaag aggtatttta

841 ttgcctcaaa aggtgtggtg cgcaagtggc aagagcaagg taataaaagg atccttgccc

901 ttaattggag aagcagattg cctccatgaa aaatacggtg gattaaacaa aagcaagcct

961 tactacacag gagaacatgc aaaggccata ggaaattgcc caatatgggt gaaaacaccc

1021 ttgaagctgg ccaatggaac caaatataga cctcctgcaa aactattaaa ggaaagaggt

1081 ttcttcggag ccattgctgg tttcttagag ggaggatggg aaggaatgat tgcaggttgg

1141 cacggataca catcccatgg ggcacatgga gtagcagtgg cagctgacct taagagcact

1201 caagaggcca taaacaagat aacaaaaaat ctcaactctt tgagtgagct ggaagtaaag

1261 aatcttcaaa gactaagcag tgccatggat gaactccaca acgaaatact agaactagat

1321 gagaaagtgg atgatctcag agctgataca ataagctcac aaatagaact cgcagtcctg

1381 ctttccaatg aaggaataat aaacagtgaa gatgaacatc tcttggcgct tgaaagaaag

1441 ctgaagaaaa tgctgggccc ctctgctgta gagataggga atggatgctt tgaaaccaaa

1501 cacaagtgca accagacctg tctcgacaga atagctgctg gtacctttga tgcaggagaa

1561 ttttctctcc ccacctttga ttcactgaat attactgctg catctttaaa tgacgatgga

1621 ttggacaatc atactatact gctttactac tcaactgctg cctccagttt ggccgtaaca

1681 ctgatgatag ctatctttgt tgtttatatg gtctccagag acaatgtttc ttgctccatt

1741 tgtctataa

//

LOCUS OR145902 1749 bp cRNA linear VRL 20-JUN-2023

DEFINITION Influenza B virus (B/Beijing/2343/2022) segment 4 hemagglutinin

(HA) gene, complete cds.

ACCESSION OR145902

VERSION OR145902

KEYWORDS .

SOURCE Influenza B virus

ORGANISM Influenza B virus

Viruses; Riboviria; Orthornavirae; Negarnaviricota;

Polyploviricotina; Insthoviricetes; Articulavirales;

Orthomyxoviridae; Betainfluenzavirus; Betainfluenzavirus

influenzae.

REFERENCE 1 (bases 1 to 1749)

AUTHORS Wang,Y.

TITLE Direct Submission

JOURNAL Submitted (20-JUN-2023) Department of Infectious Diseases, Peking

University People's Hospital, Xizhimen South Street, Beijing

100044, China

COMMENT ##Assembly-Data-START##

Sequencing Technology :: Sanger dideoxy sequencing

##Assembly-Data-END##

FEATURES Location/Qualifiers

source 1..1749

/organism="Influenza B virus"

/mol_type="viral cRNA"

/strain="B/Beijing/2343/2022"

/isolate="2343"

/isolation_source="nasal swab"

/host="Homo sapiens"

/db_xref="taxon:11520"

/segment="4"

/country="China: Beijing"

/collection_date="30-Aug-2022"

gene 1..1749

/gene="HA"

CDS 1..1749

/gene="HA"

/function="receptor binding and fusion protein"

/codon_start=1

/product="hemagglutinin"

/protein_id="WJE87873"

/translation="MKAIIVLLMVVTSNADRICTGITSSNSPHVVKTATQGEVNVTGV

IPLTTTPTKSHFANLKGTETRGKLCPKCLNCTDLDVALGRPKCTGKIPSARVSILHEV

RPVTSGCFPIMHDRTKIRQLPNLLRGYEHVRLSTQNVINTEDAPGGPYEIGTSGSCLN

ITNGKGFFATMAWAVPKNKTATNPLTIEVPYICTEEEDQITVWGFHSDDETQMARLYG

DSKPQKFTSSANGVTTHYVSQIGGFPNQTEDGGLPQSGRIVVDYMVQKSGKTGTITYQ

RGILLPQKVWCASGKSKVIKGSLPLIGEADCLHEKYGGLNKSKPYYTGEHAKAIGNCP

IWVKTPLKLANGTKYRPPAKLLKERGFFGAIAGFLEGGWEGMIAGWHGYTSHGAHGVA

VAADLKSTQEAINKITKNLNSLSELEVKNLQRLSGAMDELHNEILELDEKVDDLRADT

ISSQIELAVLLSNEGIINSEDEHLLALERKLKKMLGPSAVEIGNGCFETKHKCNQTCL

DRIAAGTFDAGEFSLPTFDSLNITAASLNDDGLDNHTILLYYSTAASSLAVTLMIAIF

VVYMVSRDNVSCSICL"

ORIGIN

1 atgaaggcaa taattgtact actcatggta gtaacatcca atgcagatcg aatctgcact

61 gggataacat cgtcaaactc accacatgtc gtcaaaactg ctactcaagg ggaggtcaat

121 gtgaccggtg taataccact gacaacaaca cccaccaaat ctcattttgc aaatctcaaa

181 ggaacagaaa ccagggggaa actatgccca aaatgcctaa actgcacaga tctggatgta

241 gccttgggca gaccaaaatg cacagggaaa ataccctctg caagggtttc aatactccat

301 gaagtcagac ctgttacatc tgggtgcttt cctataatgc atgatagaac aaaaattaga

361 cagctgccta accttctccg aggatacgaa catgtcaggt tatcaactca aaacgttatc

421 aatacagaag atgcaccagg aggaccctac gaaattggaa cctcagggtc ttgcctcaac

481 attaccaatg gaaaaggatt cttcgcaaca atggcttggg ccgtcccaaa aaacaaaaca

541 gcaacaaatc cattaacaat agaagtacca tacatttgta cagaagaaga agaccaaatt

601 accgtttggg ggttccactc tgacgacgag acccaaatgg caagactcta tggggattca

661 aagccccaga agttcacctc atctgccaac ggagtgacca cacattacgt ctcacagatt

721 ggtggcttcc caaatcaaac agaagacgga ggactaccac aaagtggcag aattgttgtt

781 gattacatgg tgcaaaaatc tggaaaaaca ggaacaatta cctatcaaag aggtatttta

841 ttgcctcaaa aggtgtggtg cgcaagtggc aagagcaagg taataaaagg atccttgccc

901 ttaattggag aagcagattg cctccatgaa aaatacggtg gattaaacaa aagcaagcct

961 tactacacag gggaacatgc aaaggccata ggaaattgcc caatatgggt gaaaacaccc

1021 ttgaagctgg ccaatggaac caaatataga cctcctgcaa aactattaaa ggaaagaggt

1081 ttcttcggag ccattgctgg tttcttagag ggaggatggg aaggaatgat tgcaggttgg

1141 cacggataca catcccatgg ggcacatgga gtagcagtgg cagctgacct taagagcact

1201 caagaggcca taaacaagat aacaaaaaat ctcaactctt tgagtgagct ggaagtaaag

1261 aatcttcaaa gactaagcgg tgccatggat gaactccaca acgaaatact agaactagat

1321 gagaaagtgg atgatctcag agctgataca ataagctcac aaatagaact cgcagtcctg

1381 ctttccaatg aaggaataat aaacagtgaa gatgaacatc tcttggcgct tgaaagaaag

1441 ctgaagaaaa tgctgggccc ctctgctgta gagataggga atggatgctt tgaaaccaaa

1501 cacaagtgca accagacctg tctcgacaga atagctgctg gtacctttga tgcaggagaa

1561 ttttctctcc ccacctttga ttcactgaat attactgctg catctttaaa tgacgatgga

1621 ttggacaatc atactatact gctttactac tcaactgctg cctccagttt ggctgtaaca

1681 ctgatgatag ctatctttgt tgtttatatg gtctccagag acaatgtttc ttgctccatt

1741 tgtctataa

//

LOCUS OR145903 1749 bp cRNA linear VRL 20-JUN-2023

DEFINITION Influenza B virus (B/Beijing/2344/2022) segment 4 hemagglutinin

(HA) gene, complete cds.

ACCESSION OR145903

VERSION OR145903

KEYWORDS .

SOURCE Influenza B virus

ORGANISM Influenza B virus

Viruses; Riboviria; Orthornavirae; Negarnaviricota;

Polyploviricotina; Insthoviricetes; Articulavirales;

Orthomyxoviridae; Betainfluenzavirus; Betainfluenzavirus

influenzae.

REFERENCE 1 (bases 1 to 1749)

AUTHORS Wang,Y.

TITLE Direct Submission

JOURNAL Submitted (20-JUN-2023) Department of Infectious Diseases, Peking

University People's Hospital, Xizhimen South Street, Beijing

100044, China

COMMENT ##Assembly-Data-START##

Sequencing Technology :: Sanger dideoxy sequencing

##Assembly-Data-END##

FEATURES Location/Qualifiers

source 1..1749

/organism="Influenza B virus"

/mol_type="viral cRNA"

/strain="B/Beijing/2344/2022"

/isolate="2344"

/isolation_source="nasal swab"

/host="Homo sapiens"

/db_xref="taxon:11520"

/segment="4"

/country="China: Beijing"

/collection_date="30-Aug-2022"

gene 1..1749

/gene="HA"

CDS 1..1749

/gene="HA"

/function="receptor binding and fusion protein"

/codon_start=1

/product="hemagglutinin"

/protein_id="WJE87874"

/translation="MKAIIVLLMVVTSNADRICTGITSSNSPHVVKTATQGEVNVTGV

IPLTTTPTKSHFANLKGTETRGKLCPKCLNCTDLDVALGRPKCTGKIPSARVSILHEV

RPVTSGCFPIMHDRTKIRQLPNLLRGYEHVRLSTQNVINTEDAPGGPYEIGTSGSCLN

ITNGKGFFATMAWAVPKNQTTTNPLTIEVPYICTEEEDQITVWGFHSDDETQMARLYG

DSKPQKFTSSANGVTTHYVSQIGGFPNQTEDGGLPQSGRIVVDYMVQKSGKTGTITYQ

RGILLPQKVWCASGKSKVIKGSLPLIGEADCLHEKYGGLNKSKPYYTGEHAKAIGNCP

IWVKTPLKLANGTKYRPPAKLLKERGFFGAIAGFLEGGWEGMIAGWHGYTSHGAHGVA

VAADLKSTQEAINKITKNLNSLSELEVKNLQRLSGAMDELHNEILELDEKVDDLRADT

ISSQIELAVLLSNEGIINSEDEHLLALERKLKKMLGPSAVEIGNGCFETKHKCNQTCL

DRIAAGTFDAGEFSLPTFDSLNITAASLNDDGLDNHTILLYYSTAASSLAVTLMIAIF

VVYMVSRDNVSCSICL"

ORIGIN

1 atgaaggcaa taattgtact actcatggta gtaacatcca atgcagatcg aatctgcact

61 gggataacat cgtcaaactc accacatgtc gtcaaaactg ctactcaagg ggaggtcaat

121 gtgaccggtg taataccact gacaacaaca cccaccaaat ctcattttgc aaatctcaaa

181 ggaacagaaa ccagggggaa actatgccca aaatgcctaa actgcacaga tctggatgta

241 gccttgggca gaccaaaatg cacagggaaa ataccctctg caagggtttc aatactccat

301 gaagtcagac ctgttacatc tgggtgcttt cctataatgc atgatagaac aaaaattaga

361 cagctgccta accttctccg aggatacgaa catgtcaggt tatcaactca aaacgttatc

421 aatacagaag atgcaccagg aggaccctac gaaattggaa cctcagggtc ttgcctcaac

481 attaccaatg gaaaaggatt cttcgcaaca atggcttggg ccgtcccaaa aaaccaaaca

541 acaacaaatc cattaacaat agaagtacca tacatttgta cagaagaaga agaccaaatt

601 accgtttggg ggttccactc tgacgacgag acccaaatgg caaggctcta tggggattca

661 aagccccaga agttcacctc atctgccaac ggagtgacca cacattacgt ctcacagatt

721 ggtggcttcc caaatcaaac agaagacgga ggactaccac aaagtggcag aattgttgtt

781 gattacatgg tgcaaaaatc tggaaaaaca ggaacaatta cctatcaaag aggtatttta

841 ttgcctcaaa aggtgtggtg cgcaagtggc aagagcaagg taataaaagg atccttgccc

901 ttaattggag aagcagattg ccttcatgaa aaatacggtg gattaaacaa aagcaagcct

961 tactacacag gggaacatgc aaaggccata ggaaattgcc caatatgggt gaaaacaccc

1021 ttgaagctgg ccaatggaac caaatataga cctcctgcaa aactattaaa ggaaagaggt

1081 ttcttcggag ccattgctgg tttcttagag ggaggatggg aaggaatgat tgcaggttgg

1141 cacggataca catcccatgg ggcacatgga gtagcagtgg cagctgacct taagagcact

1201 caagaggcca taaacaagat aacaaaaaat ctcaactctt tgagtgagct ggaagtaaag

1261 aatcttcaaa gactaagcgg tgccatggat gaactccata acgaaatact agaactagat

1321 gagaaagtgg atgatctcag agctgataca ataagctcac aaatagaact cgcagtcctg

1381 ctttccaatg aaggaataat aaacagtgaa gatgaacatc tcttggcgct tgaaagaaag

1441 ctgaagaaaa tgctgggccc ctctgctgta gagataggga atggatgctt tgaaaccaaa

1501 cacaagtgca accagacctg tctcgacaga atagctgctg gtacctttga tgcaggagaa

1561 ttttctctcc ccacctttga ttcactgaat attactgctg catctttaaa tgacgatgga

1621 ttggacaatc atactatact gctttactac tcaactgctg cctccagttt ggctgtaaca

1681 ctgatgatag ctatctttgt tgtttatatg gtctccagag acaatgtttc ttgctccatt

1741 tgtctataa

//
